# Practical synthetic strategies towards lipophilic 6-iodotetrahydroquinolines and -dihydroquinolines

**DOI:** 10.3762/bjoc.12.174

**Published:** 2016-08-16

**Authors:** David R Chisholm, Garr-Layy Zhou, Ehmke Pohl, Roy Valentine, Andrew Whiting

**Affiliations:** 1 Centre for Sustainable Chemical Processes, Department of Chemistry, Durham University, South Road, Durham, DH1 3LE, UK; 2 Biophysical Sciences Institute, Durham University, South Road, Durham, DH1 3LE, UK; 3 High Force Research Limited, Bowburn North Industrial Estate, Bowburn, Durham, DH6 5PF, UK

**Keywords:** cyclisation, dihydroquinoline, elimination, reduction, tetrahydroquinoline

## Abstract

The synthesis of novel tetrahydroquinolines (THQ) and dihydroquinolines (DHQ) are reported using three practical, scalable synthetic approaches to access highly lipophilic analogues bearing a 6-iodo substituent, each with a different means of cyclisation. A versatile and stable quinolin-2-one intermediate was identified, which could be reduced to the corresponding THQ with borane reagents, or to the DHQ with diisobutylaluminium hydride via a novel elimination that is more favourable at higher temperatures. Coupling these strongly electron-donating scaffolds to electron-accepting moieties caused the resulting structures to exhibit strong fluorescence.

## Introduction

Tetrahydroquinolines (THQ) and dihydroquinolines (DHQ) are heterocyclic scaffolds that are ubiquitous in natural products, therapeutics, fluorophores and dyes [1]. Both are structures of great versatility, and their physical and chemical properties can be finely tuned using synthetic chemistry. Methods for their synthesis have been well studied, and range from the classic Skraup–Doebner–von Miller syntheses, to catalytic and asymmetric methods [2–4].

**Figure 1:**
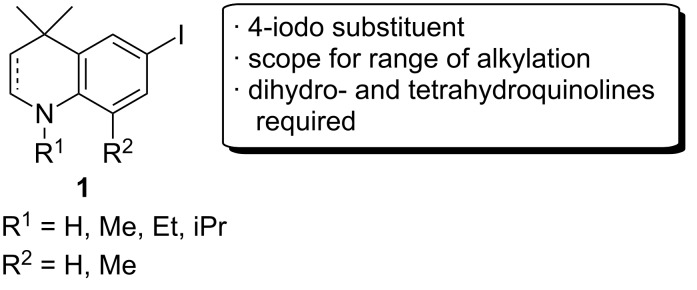
Tetrahydroquinoline (THQ) and dihydroquinoline (DHQ) scaffolds to be synthesised.

We required a straightforward synthesis of THQs and DHQs for the synthesis of a library of biocompatible fluorophores with the potential to be used in fluorescence microscopy applications. The desired intermediates were to be highly lipophilic, derivatisable and easy to synthesise on both small and multigram scales. A scaffold of general type **1** (Figure 1) was chosen as a synthetic target that could be *N*-alkylated with bulky alkyl groups for increased lipophilicity. An iodo substituent in the *para*-position relative to nitrogen (the 6-position) was required for further functionalisation by typical cross-coupling reactions.

THQ scaffolds similar to **1** are known in the literature, and have been typically prepared by two approaches. The first involves the alkylation of the starting aniline with the inexpensive 3,3-dimethylallyl bromide/chloride, followed by cyclisation mediated by Lewis acids, or under acidic conditions [5–6]. A second, analogous approach involves an initial acylation with the commercially available 3,3-dimethylacryloyl chloride, followed by Friedel–Crafts cyclisations that also use Lewis acids [7]. The resultant quinolin-2-one is then reduced using strong hydride reducing agents such as LiAlH_4_ [8]. Similar THQs have also been prepared by a reductive Beckmann rearrangement of an oxime using diisobutylaluminium hydride (DIBAL) [9]. In contrast to this variety, the corresponding 1,4-DHQ of scaffold **1** has not been synthesised to our knowledge. Similar DHQs are typically prepared by methods such as ring-closing metathesis, or by controlled Birch-type reductions of the corresponding quinoline [10–12].

Very few instances of iodine-containing THQs or DHQs exist in the literature [13]. An initial investigation with the corresponding bromides indicated that these were unreactive towards many cross-coupling methodologies, presumably due to the highly electron-rich arene [14]. Reactions such as the Sonogashira coupling, for example, often required high catalyst loadings, phosphine additives and strong heating or microwave conditions in order to effect coupling with most acetylenes. Accordingly, it was anticipated that the aryl iodides would be more versatile due to their greater propensity to undergo oxidative additions.

Therefore, in this paper we report herein the development of facile, scalable and practical syntheses for 6-iodo-THQ and 6-iodo-DHQ scaffolds that can be employed in a variety of cross-coupling and derivatisation reactions. Three synthetic routes are described, each of which involves a different means of cyclisation to form the heterocyclic structure. We also describe a novel formation of the 1,4-DHQ of scaffold **1**; a reaction that appears to be favoured both by the use of hydride reducing agents that produce stable tetrahedral intermediates and the high temperature collapse of these intermediates.

## Results and Discussion

The aim of this investigation was to synthesise THQs and DHQs of the general scaffold **1** for the use in a variety of cross-coupling reactions, thus necessitating the presence of a halogen or pseudohalogen in the *para*-position relative to nitrogen. Most of the relevant literature focuses on the synthesis of the corresponding aryl bromides or aryl chlorides, but initial investigation with 6-bromo-THQs indicated that these were remarkably unreactive in cross-coupling reactions [14], and a reliable and scalable synthesis of the likely more reactive iodides was, therefore, sought.

**Scheme 1:**
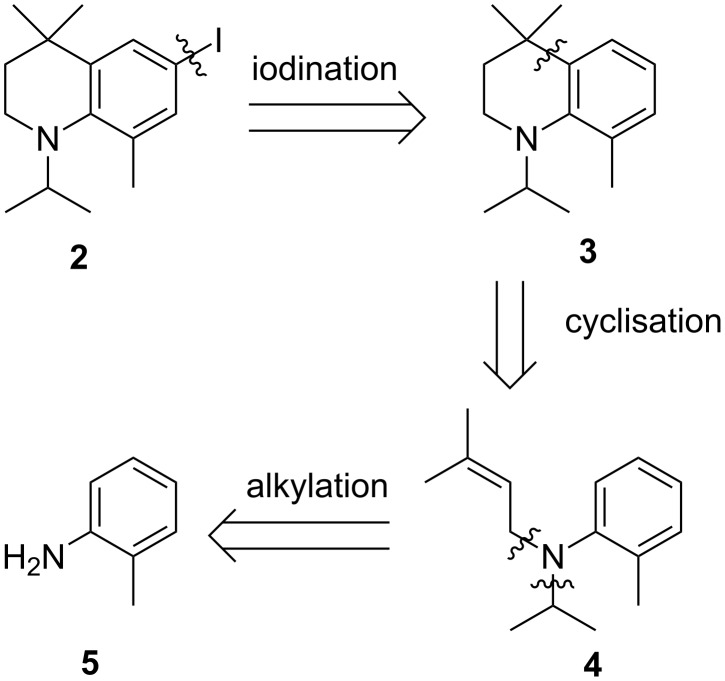
Proposed retrosynthesis scheme to access *N*-isopropyl-THQ **2**.

We first considered the retrosynthetic analysis shown in Scheme 1, which requires cyclisation from the alkylated *N*-allylaniline **4**. The use of polyphosphoric acid (PPA) in cyclisation reactions is well known in the literature, and has been used to form a large variety of ring structures [15]. Iodination of **3** could then be realised by utilising the rich iodination literature available [16–17].

**Scheme 2:**
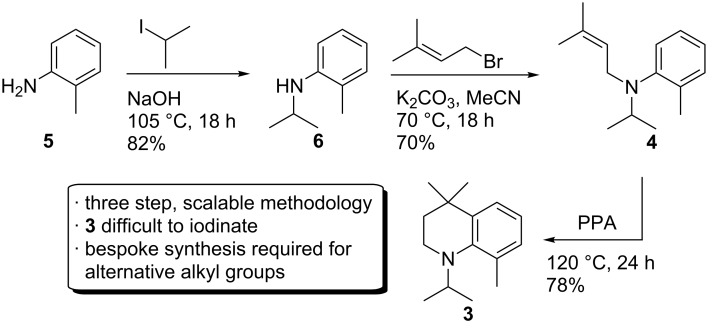
Synthesis of THQ **3** by initial *N*-alkylations, followed by PPA-mediated cyclisation.

Isopropylation of the inexpensive *o*-toluidine (**5**) can be conducted by simply adding NaOH and 2-iodopropane to a refluxing solution of the aniline (Scheme 2). While this straightforward procedure was well suited to larger scale, some bis-alkylation was difficult to avoid. However, the desired product **6** can be isolated using basic alumina chromatography. Reductive amination with acetone, mediated by AcOH and NaOAc was also pursued as an alternative strategy [18]. While being much easier to purify due to the elimination of the bis-alkylation product, the yield of this approach was much more variable at larger scales.

Further *N*-alkylation with 3,3-dimethylallyl bromide was conducted using K_2_CO_3_. The resulting alkylated compound **4** was easily isolated by chromatography in good yield, however, the reaction was found to be difficult to force to completion, presumably due to steric hindrance. Cyclisation of **4** to the THQ **3** was realised by heating a PPA mixture of **4** to 120 °C.

**Scheme 3:**
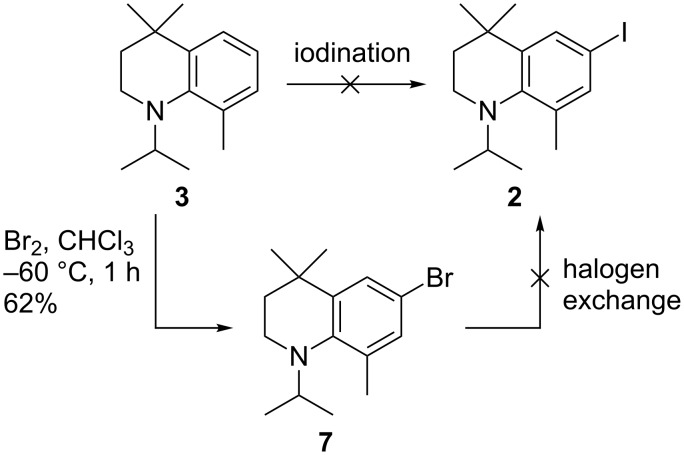
Bromination of **3** and attempted halogen exchange of the intermediate **7**.

Iodination of **3** using a variety of reagents including I_2_/HgO [19], ICl/AcOH, *N*-iodosuccinimide/TFA [20], KI/KIO_3_/AcOH [21], and *N*-chlorosuccinimide/NaI/AcOH [22], all gave poor yields (0–30%) according to GC–MS analysis, and the iodinated product **2** was difficult to isolate by chromatography. In light of these results, bromination was conducted with the aim of synthesising the corresponding aryl iodide **2** by halogen exchange (Scheme 3).

After careful optimisation of the reaction conditions, the bromide **7** was exclusively obtained by addition of 0.95 equivalents of bromine to a solution of **3** in chloroform maintained at −60 °C. However, halogen exchange under literature conditions was sluggish and even after 3 days only around 30% of the starting material had converted [23].

**Scheme 4:**
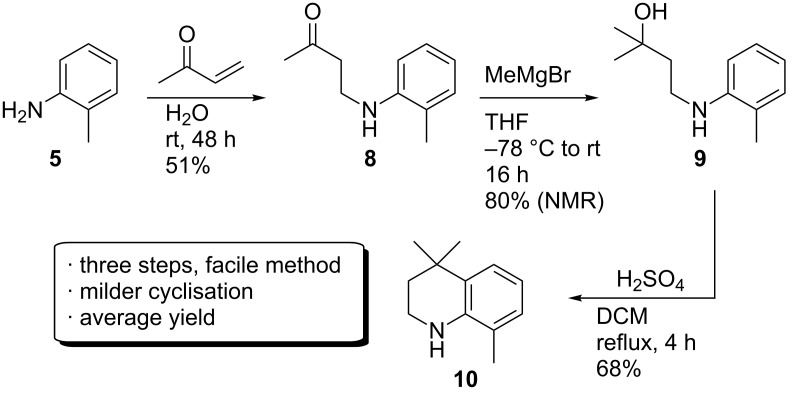
Synthesis of THQ **10**, by initial aza-Michael addition, followed by formation of the tertiary alcohol **9**, which was then cyclised with H_2_SO_4_.

It was theorised that the low reactivity of **3** towards electrophilic aromatic iodination was caused by distortion of the THQ ring structure due to the need to minimise steric interactions between the *N*-iPr group and the neighbouring methyl group. This may result in poorer orbital overlap between the nitrogen lone pair and the aromatic π-system, thus reducing the sp^2^ character of the nitrogen, and therefore lowering the reactivity of the system towards electrophilic aromatic substitution. An alternative, analogous synthesis was accordingly devised, in which the unsubstituted THQ **10** was targeted, as outlined in Scheme 4 [24].

Reports of aza-Michael additions occurring in water indicated that the desired **8** could be synthesised under mild conditions, and we therefore decided to adapt these published conditions to larger scale work [25–26]. Initial attempts involving a 1:1 molar mixture of *o*-toluidine (**5**) and methyl vinyl ketone (MVK) indicated that only around 60% of the starting aniline had converted, particularly at larger scales (>1 g). Two equivalents of MVK were required to effect full conversion, however, under these conditions the bis-adduct was formed in around 6–10% and this was difficult to remove by chromatography or distillation. Using one equivalent lowered the yield, but minimised bis-adduct formation, which allowed facile purification by short path distillation on larger scales. Compound **8** was functionalised to the tertiary alcohol **9** by a Grignard reaction with MeMgBr, which could be directly cyclised without purification to **10** by heating a DCM solution with a nominal amount of concentrated sulphuric acid. This procedure was much more straightforward when compared to the previous PPA reaction, and required a much less laborious work-up.

The increased efficiency of this reaction can be attributed to improved orbital overlap. Protonation of the hydroxy group of **9** under the reaction conditions likely leads to the corresponding tertiary carbocation under equilibrium. The nitrogen lone pair can then assist the electrophilic cyclisation reaction, augmented by improved orbital overlap with the aromatic π-system, which in the case of **9** would be improved over **4** due to the lack of a second alkyl substituent and diminished steric repulsion that likely causes rotation of the nitrogen lone pair out of conjugation with the aromatic ring.

While the increased availability of the nitrogen lone pair appears to assist cyclisation in this system, iodination of **10** was still relatively low yielding, particularly with acidic iodination methods (presumably due to protonation and resultant deactivation of the nitrogen). Slightly higher yields were obtained with pyridine–iodine [16], however, the conversion was also low and isolation of the iodinated product was often difficult.

Despite the relative ease, the overall yield of this second route was considered too low to be a reliable source of THQs of the general scaffold **1**. However, we were encouraged by the simplicity and ease of the H_2_SO_4_-mediated cyclisation reaction, and a third synthetic route was therefore devised that would employ this methodology.

A common approach in the literature towards similar THQs involves an initial acylation of the starting aniline using 3,3-dimethylacryloyl chloride, followed by a high temperature cyclisation employing Lewis acids such as AlCl_3_ [7,27]. However, initial experiments indicated that under these cyclisation conditions, significant degradation and de-iodination occurred with iodinated intermediates. It was anticipated that the milder H_2_SO_4_ conditions could affect the cyclisation without these side reactions. To probe this, commercially available 4-iodo-2-methylaniline (**11**) was employed as the starting material in order to circumvent the low-yielding iodination step. This starting material is also readily synthesised from *o*-toluidine (**5**) using a pyridine–iodine iodination [16].

**Scheme 5:**
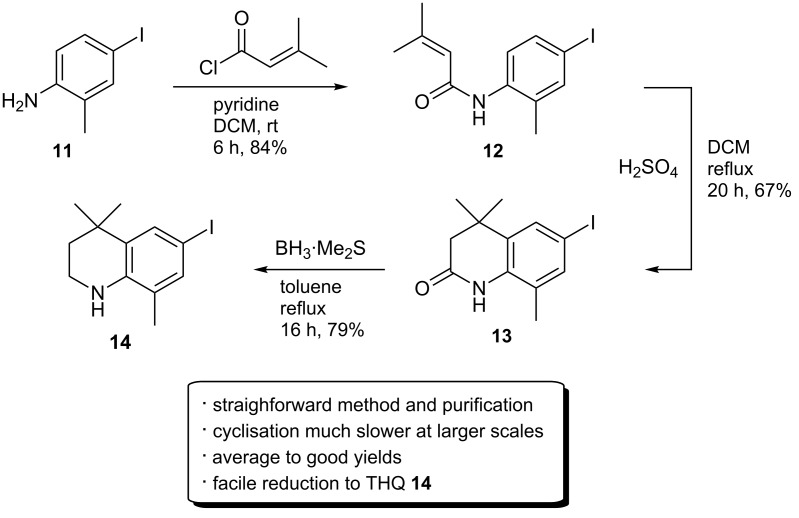
Synthesis of THQ **14** by initial acylation, cyclisation with H_2_SO_4_ and reduction with borane·dimethyl sulphide complex.

Initial acylation of **11** with 3,3-dimethylacryloyl chloride and pyridine provided **12** in good yield (Scheme 5). The acid-catalysed reaction with **12** was predicted to proceed via initial formation of the corresponding tertiary alcohol involving a Markovnikov addition, before cyclisation as with **10**. Indeed, cyclisation product **13** (see Supporting Information File 1 for crystal structure) was produced cleanly in a 67% yield, which was easily separated chromatographically from the remaining starting material. However, the reaction occurred at a significantly slower rate than with **9**; particularly on larger reaction scales where complete conversion of the starting material required long reaction times (>48 h). The significantly slower rate is likely due to the reduced availability of the nitrogen lone pair in **12**. Favourably, however, no signs of de-iodination or degradation were observed under these cyclisation conditions. Cyclisation product **13** was readily reduced to the iodinated THQ **14** (see Supporting Information File 1 for crystal structure) using borane·dimethyl sulphide complex. A slight molar excess of the reducing agent causes de-iodination to give **10** in small quantities.

**Scheme 6:**
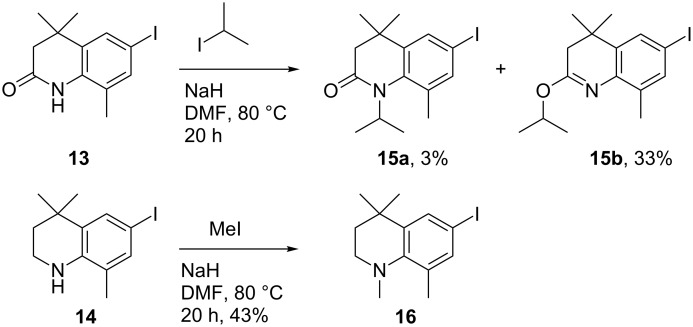
*N*-Alkylation of **13** and **14**.

*N*-Alkylation with highly lipophilic alkyl groups such as an isopropyl was next considered (Scheme 6). The quinolin-2-one **13** was predicted to be a more amenable alkylation partner than the THQ **14** due to the likely lower p*K*_a_ of the amide proton. To assess this, **13** was reacted with NaH and 2-iodopropane in DMF at 80 °C overnight. However, **13** displayed a marked lack of reactivity towards isopropylation, and only around 40% of the starting material **13** was converted. Interestingly, the major product from the reaction was the *O*-iPr imine **15b** as indicated by a low field ^1^H chemical shift of the isopropyl proton (5.39 ppm), and later confirmed by X-ray crystallography (the crystal structure is shown in Supporting Information File 1). The *N*-iPr product **15a** was isolated in only 3% yield. This, therefore, indicates that the electrophile reacts faster with the oxide anion of **13**. Repeating the reaction with 15-crown-5 to limit the effect of the sodium cation did not appreciably effect the product distribution. It would, therefore, appear that steric hindrance from the neighbouring methyl group is in fact the main determinant of the product distribution.

Alkylation of **14** was also marked by a general lack of reactivity; the methylated **16** was isolated in only a 43% yield. Ethylation was possible, albeit in much lower yield and only negligible amounts (1–2%) of the iPr adduct were isolated. Alkylation using silver(I) oxide was also possible, but with similarly low yields.

The *ortho*-methyl group had originally been incorporated as protection to block the *ortho*-amino centre against oxidation [14], and to increase lipophilicity. However, because the methyl group appeared likely to be the cause for the lack of reactivity of **13** and **14** towards alkylation, a synthesis of the corresponding quinolin-2-one derived from 4-iodoaniline (**17**) was pursued in order to increase the ease of alkylation.

The initial acylation of **17** (commercially available, or easily synthesised using literature methods [16]) proceeded well to give **18**, and this could easily be applied to larger scale (>25 g) synthesis. The acid-catalysed cyclisation again was much slower than for the synthesis of **10**, and at large scale in particular; the reaction could take at least 48 hours for completion. An alternative large-scale protocol was, therefore, investigated. In contrast to the high temperature Lewis acid-mediated cyclisations reported in the literature [7], it was found that simply stirring a DCM solution of **18** with 1.5 equivalents of AlCl_3_ at room temperature for 2.5 hours gave the quinolin-2-one **19** in excellent yield after recrystallisation from EtOH (see Supporting Information File 1 for the crystal structure). While facile, the reaction was highly dependent both on the number of equivalents of AlCl_3_ used and the reaction time. Leaving the reaction mixture to stir with 1.5 equivalents of AlCl_3_ for longer periods (>3 h) resulted in minor de-iodination, whereas less than 2 hours gave incomplete cyclisation. Using 2 equivalents of AlCl_3_ causes rapid cyclisation after 1 hour, but the de-iodinated form was also present. Using 1.25 equivalents appeared to inhibit de-iodination completely, but the cyclisation reaction tended to plateau before full completion.

**Scheme 7:**
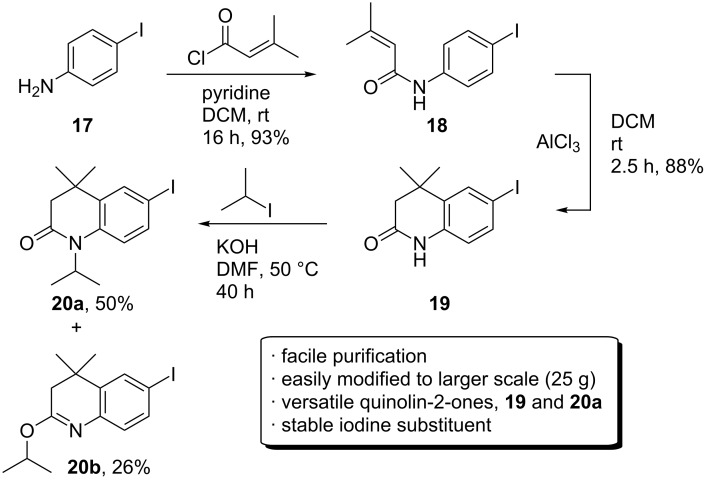
Facile route for the synthesis of **20a**.

Without the *ortho*-methyl, preference for isopropylation switched to *N*-alkylation, providing 50% of **20a** and 26% of the *O*-isopropylimine **20b**; a mixture that could be separated by chromatography. Full conversion of the starting material was difficult to achieve and appeared to plateau at around 75%. However, the reaction was easily conducted and both products isolated on larger scale (30 g). Encouragingly, the iodine substituent was stable throughout the optimised synthesis of the quinolin-2-one intermediate (Scheme 7).

**Scheme 8:**
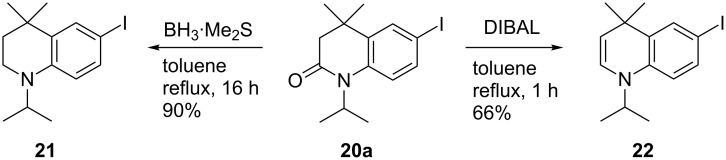
Synthesis of THQ **21** and DHQ **22** using borane·dimethyl sulphide complex or DIBAL, respectively.

**Figure 2:**
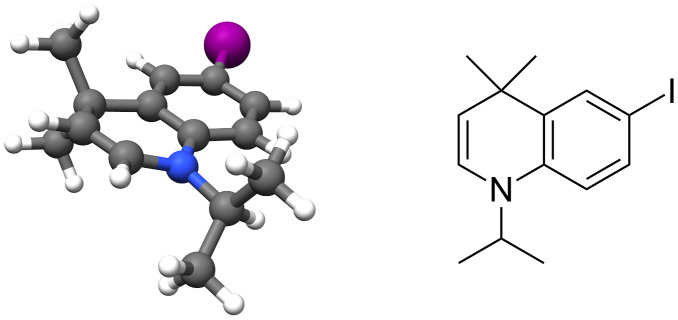
Simulated structure of **22** indicates a flattened quinoline-like structure. Hartree–Fock calculations (3-21G*) were conducted using Spartan’10, and visualised using UCSF Chimera 1.11 [28–29].

Reduction to the THQ **21** was easily conducted with borane·dimethyl sulphide complex as described previously, in excellent yield (Scheme 8). In addition, upon trialling different conditions for the reduction, LiAlH_4_ was found to afford a small amount (ca. 5%) of 1,4-DHQ **22** in addition to **21**. Ab initio simulation of **22** (Figure 2) indicated that the enamine functionality causes a flattening of the dihydroquinoline ring to give a more planar, quinoline-like structure. We were intrigued by the result of these stereoelectronic and conformational effects in terms of the chromophoric properties of the resulting systems and an improved synthesis of **22** was therefore pursued.

In light of its formation by LiAlH_4_, we anticipated that production of **22** would be favoured if the intermediate tetrahedral complex in the reduction reaction was more stable. An elimination reaction could then operate to give **22**, either during aqueous work-up, or in situ collapse of the intermediate complex and subsequent reduction of the resulting iminium ion via elimination. As a Lewis acidic reducing agent, it was anticipated that DIBAL could be better suited to the reduction of the electron-rich amide of **20a**, and DIBAL is also known to produce relatively stable tetrahedral intermediates [30]. Indeed, initial experiments using 1 equivalent of DIBAL in THF at reflux, with an acidic work-up, returned a 35% yield of **22** by ^1^H NMR, along with 15% of **21**, and unreacted starting material.

This result highlighted an apparent lack of reactivity in THF and the reaction was, therefore, monitored in THF, DCM and toluene using in situ FTIR spectroscopy (ReactIR). The amide carbonyl stretch of **20a** was followed by IR during the addition of one equivalent of DIBAL at rt (see Supporting Information File 1 for details). The reactions in DCM and toluene indicated a rapid reduction of the amide carbonyl stretch. In contrast, the reaction in THF displayed a lower initial addition rate, presumably due to competitive coordination of DIBAL by the solvent, and appeared to plateau at a low conversion. A further addition of 1 equivalent of DIBAL did further consume the amide (shown by a further loss of the C=O stretch by IR), however, again, progress appeared to plateau. This indicated that the reaction with the amide group was markedly faster in a non-polar solvent, and further work suggested that the yield of **22** was also increased under such conditions. Addition of DIBAL at lower temperatures (0, −40, and −78 °C) was found to significantly slow the reaction in all solvents.

**Scheme 9:**
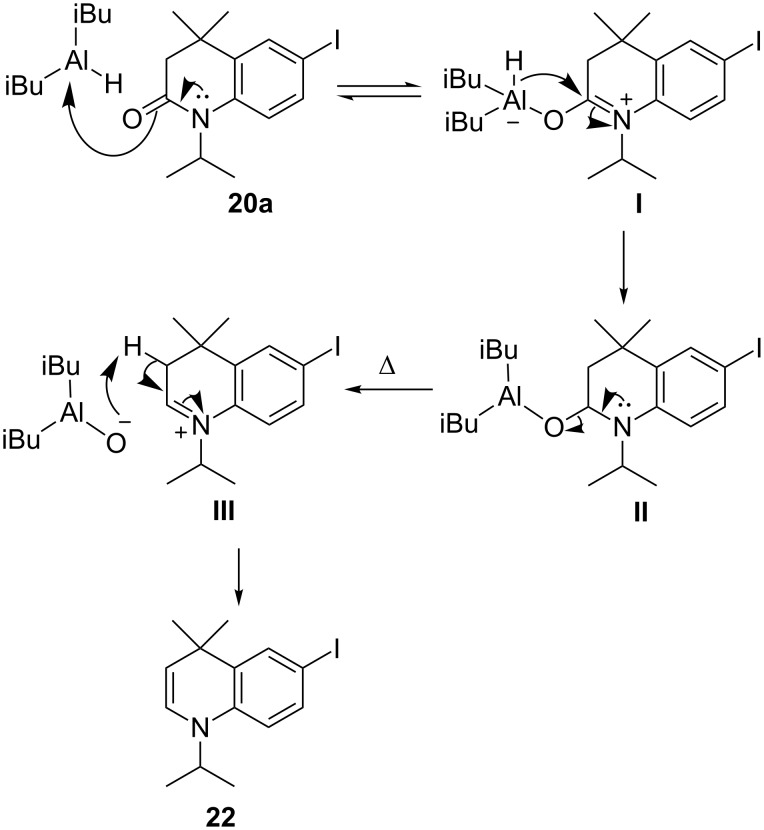
Postulated mechanism for the formation of **22** using DIBAL.

Further investigation also highlighted the interesting role of the temperature of the reaction mixture prior to the addition of DIBAL on the product distribution. Increased NMR yields of DHQ **22** relative to THQ **21** were recorded (as shown in Table 1), as the temperature of the **20a** solution was increased prior to DIBAL addition. This trend indicates that **22** may be formed from in situ fragmentation of the intermediate DIBAL complex followed by an elimination process that is favoured at higher temperature, as postulated in Scheme 9.

**Table 1:** Temperature and solvent effects from the addition of DIBAL to **20a**.^a^

Solvent	Temp. (°C)	**20a** (%)	**21** (%)	**22** (%)

pentane	rt	26	19	55
cyclohexane	60	2	14	79
cyclohexane	81	0	15	85
toluene	111	10	7	83
xylenes	140	13	4	83

^a^One equivalent of DIBAL (1.0 M in cyclohexane) added to preheated solution of **20a** (ca. 0.25 mmol, in 4 mL), stirred rapidly for 1 h, quenched with 20% aq w/v NaOH, extracted in EtOAc, washed with H_2_O and brine, dried (MgSO_4_) and evaporated. Yields of **21** and **22** estimated from ^1^H NMR analysis of the crude mixture.

Chromatographic separation of compounds **21** and **22** on silica resulted in co-elution; however, the two compounds could be separated on neutral alumina using a non-polar eluent. Selectivity for **22** on larger scales was slightly lower, though the DHQ was still isolated in a satisfactory 66% yield on one gram scale. Unlike borane, LiAlH_4_ and similar reagents, DIBAL did not cause de-iodination, even with larger excesses (up to 2 equivalents).

There are few references to this type of reaction in the literature, however, to our knowledge this is the only such reaction reported with an aromatic amide [31]. Organolithium reagents have been used to synthesise the analogous alkylated DHQ compounds [27].

Compounds **21** and **22** were found to undergo slow degradation (over 2–4 weeks), as indicated by the observation that solutions in CDCl_3_ slowly turned pink (**22** in particular). However, these compounds did not require special handling or precautions, and could be further derivatised without issue. In contrast, **20a** was stable indefinitely when stored neat, or in solution at room temperature.

This interesting reaction was conducted with a variety of quinolin-2-ones bearing differing alkyl substituents in order to ascertain whether steric effects promote collapse of the intermediate aluminium complex via fragmentation/elimination, and to assess the generality of this approach. Table 2 highlights the effect of the presence of *N*-alkyl groups, and an aryl group in the benzylic position of the quinolin-2-one on the synthesis of the corresponding DHQs using the DIBAL reaction developed for the synthesis of **22**.

**Table 2:** DIBAL reductions of quinolin-2-ones **23a**–**e** using the optimised method to synthesise **22**.^a^

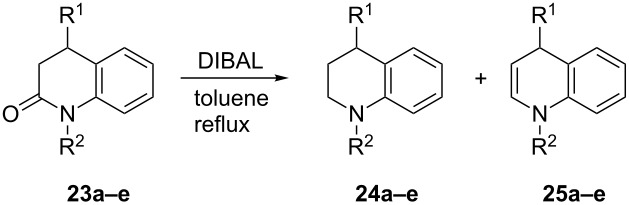					

Quinolin-2-one	R^1^	R^2^	**23** (%)	**24** (%)	**25** (%)

**23a**	Ph	H	0	100	0
**23b**	Ph	Me	0	38	62
**23c**	H	H	12	88	0
**23d**	H	Et	0^b^	63^b^	21^b^
**23e**	H	Bn	0^c^	35^c^	Trace^c^

**^a^**DIBAL (1.0 M in toluene, 1.2 equivalents or 2.0 equivalents for **23a** and **23c**) added to a refluxing solution of quinolin-2-one **23a**–**e** (0.5–3.0 mmol) in toluene stirred rapidly at reflux until TLC analysis indicated completion. Then quenched with 20% aq w/v NaOH, extracted with EtOAc, washed with H_2_O and brine, dried (MgSO_4_) and evaporated. Yields of **23a–e**, **24a–e** and **25a–e** were estimated from ^1^H NMR analysis of the crude mixture (see Supporting Information File 1). ^b^A ring-opened ethylaniline (9%) and another byproduct were also present (7%). ^c^A ring-opened benzylaniline was also present (61%).

The absence of an *N*-alkyl group (**23a** and **23c**) was found to completely disfavour the formation of the corresponding DHQ. Conducting these reactions with 1.2 equivalents of DIBAL resulted in low conversion (30–40%) to the corresponding THQs (**24a** and **24c**), presumably due to competitive deprotonation of the amide proton by DIBAL. When repeated with 2.0 equivalents, **24a** and **24c** were cleanly synthesised in 100% and 88% yields according to ^1^H NMR analysis of the crude mixtures.

Addition of an *N*-alkyl group to supplement the steric effect of the benzylic phenyl (*N*–Me, **23b**) resulted in the reaction favouring the production of the DHQ **25b** (62%) over the THQ **24b** (38%). Retaining the *N*-alkyl group whilst removing the steric effect of this benzylic phenyl group (**23d**) now switched the preference of the reaction towards the THQ **24d** (63%) compared to DHQ **25d** (21%). In contrast to the clean conversions of the other quinolin-2-ones, crude ^1^H NMR analysis of the DIBAL reduction of **23d** indicated a variety of side products, chief among which was the corresponding ring opened *N*–ethylaniline (9%). Indeed, when moving to the *N*–benzylquinolin-2-one **23e**, the major product was that of the ring opened *N-*benzylaniline (61%) and was favoured over the THQ **24e** (3%) and only trace indications of DHQ **25e** were apparent.

While these results indicated that this reaction was not completely general, comparison of the NMR yields began to provide further mechanistic insight. Clearly, Table 2 shows that an *N*-alkyl substituent is required in order to encourage formation of the DHQ. The presence of larger *N*-substituents (**23d**,**e**) did little to improve the yield of the corresponding DHQ over **23b**, thus indicating that the presence of an alkyl group simply retards reduction of the imine to the THQ, such that the competing elimination process can operate.

A clear increase in the yield of the DHQ is also observed upon addition of a steric influence in the benzylic position of the quinolin-2-one (**20a**/**23b**). Given the high temperature, it may be that 1,3-diaxial steric interactions between the benzylic substituent(s) and the intermediary iminium ion are such that a conformation needs to be adopted that both allows and indeed, favours the proposed elimination pathway, with the expelled DIBAL-derived aluminate likely functioning as the base.

**Figure 3:**
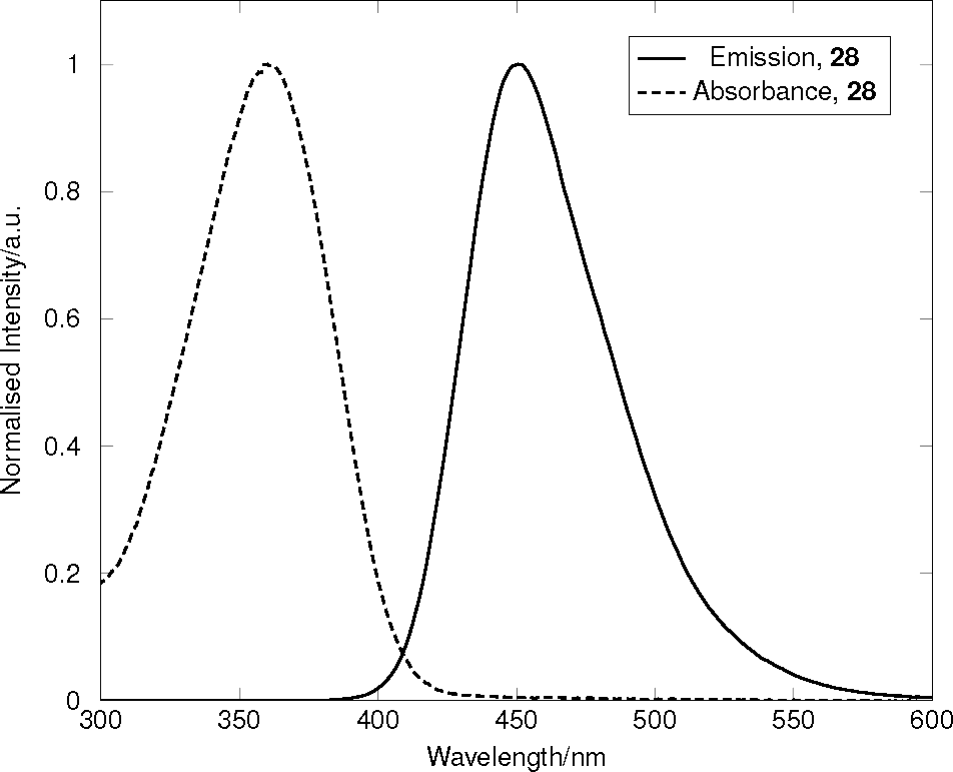
Combined, normalised absorption and emission spectra of **28** in chloroform. Absorption spectrum was recorded at 10 μM. Emission spectrum was recorded at 100 nM, with excitation at 360 nm.

With optimised syntheses of **21** and **22** in hand, the reactivity of their iodo substituents towards cross-coupling reactions was compared to that of the corresponding aryl bromides. Table 3 shows an example where **21** and 6-bromo-THQ **26** (synthesised using the optimised conditions for **21**) were reacted under typical Suzuki conditions with boronic ester **27**. Analysis of the crude ^1^H NMR spectra for the reactions indicated full conversion of the 6-iodo-THQ **21**, but moderate conversion (56%) in the case of the 6-bromo-THQ **26**. This highlights the greater propensity for **21** to undergo oxidative addition, due to the more reactive iodo-substituent. Further analysis indicated that biaryl **28** strongly absorbs light at 360 nm, and when excited at this wavelength exhibited a red-shifted, intense, emission peaking at 450 nm (Figure 3), thus highlighting the chromophoric effect of attaching a conjugated electron acceptor to the strongly electron donating THQ moiety.

**Table 3:** Comparing the reactivity of 6-iodo-THQ **21** and 6-bromo-THQ **26** in a typical Suzuki reaction with boronic ester **27** to give biaryl **28**.

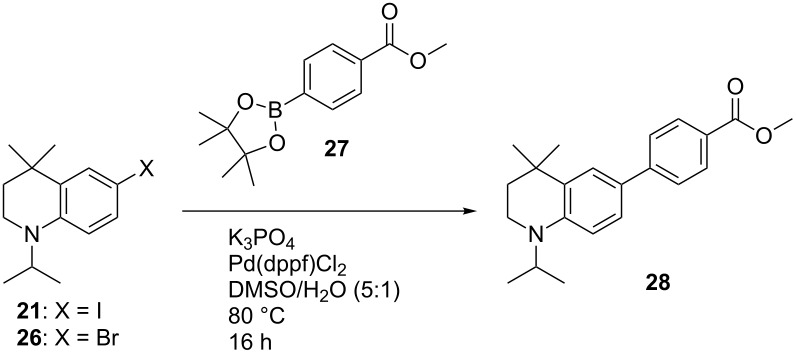			

THQ	Halide	Conversion of starting material^a^	Isolated yield of **28**

**21**	I	100%	68%
**26**	Br	56%	N/A^b^

**^a^**Conversion of the starting THQs **21**/**26** to the coupling product **28** was estimated by comparing the integrals of the CH_2_ adjacent to the nitrogen (the 2-position) in the crude ^1^H NMR spectrum. ^b^Not isolated.

**Scheme 10:**
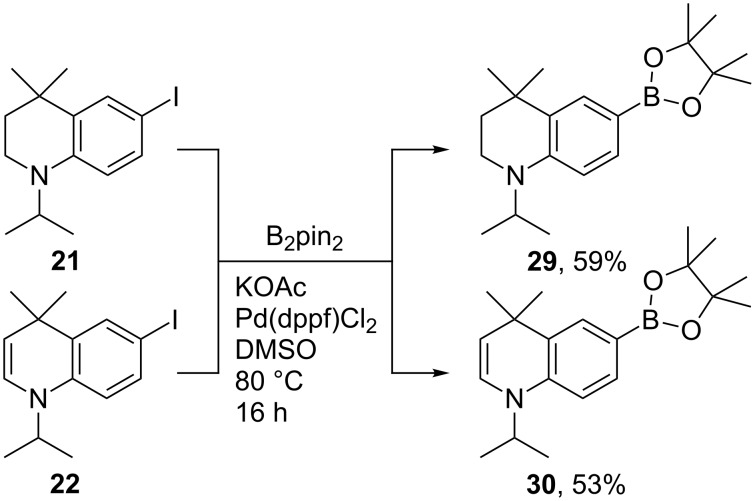
Miyaura borylation of **21** and **22** to give crystalline boronic esters **29** and **30**.

**Figure 4:**
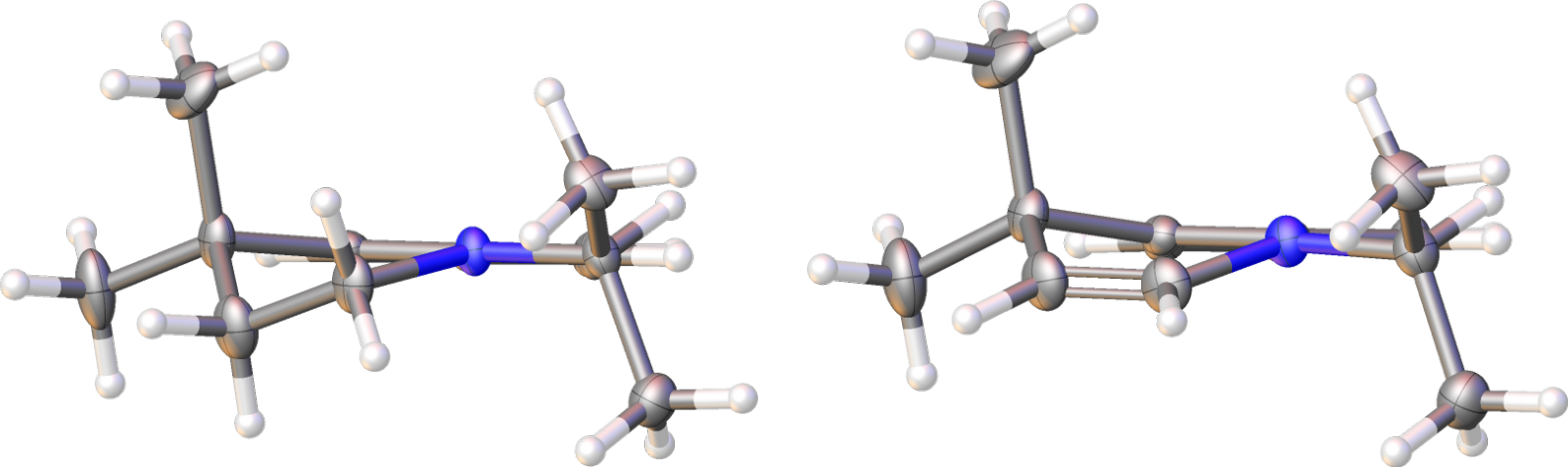
Comparison of the crystal structures of **29** (left) and **30** (right) as viewed along the plane of the aromatic ring, with the pinacolate ester groups removed for clarity. DHQ **30** exhibits a more planar structure. Compounds **29** and **30** were visualised using Olex2 [33].

To enable a wider range of cross-coupling reactions and to compare their 3-dimensional structures, the corresponding boronic esters **29** and **30** were prepared from **21** and **22** using typical Miyaura borylation conditions (Scheme 10) [32]. Both were highly crystalline compounds, and comparison of the two crystal structures (Figure 4) highlighted the more planar structure of **30** as a result of the enamine function, as predicted by ab initio methods in Figure 2. Furthermore, TLC samples of **30** were found to be fluorescent under a UV lamp, and an absorbance/emission analysis was accordingly conducted in diethyl ether and chloroform.

**Figure 5:**
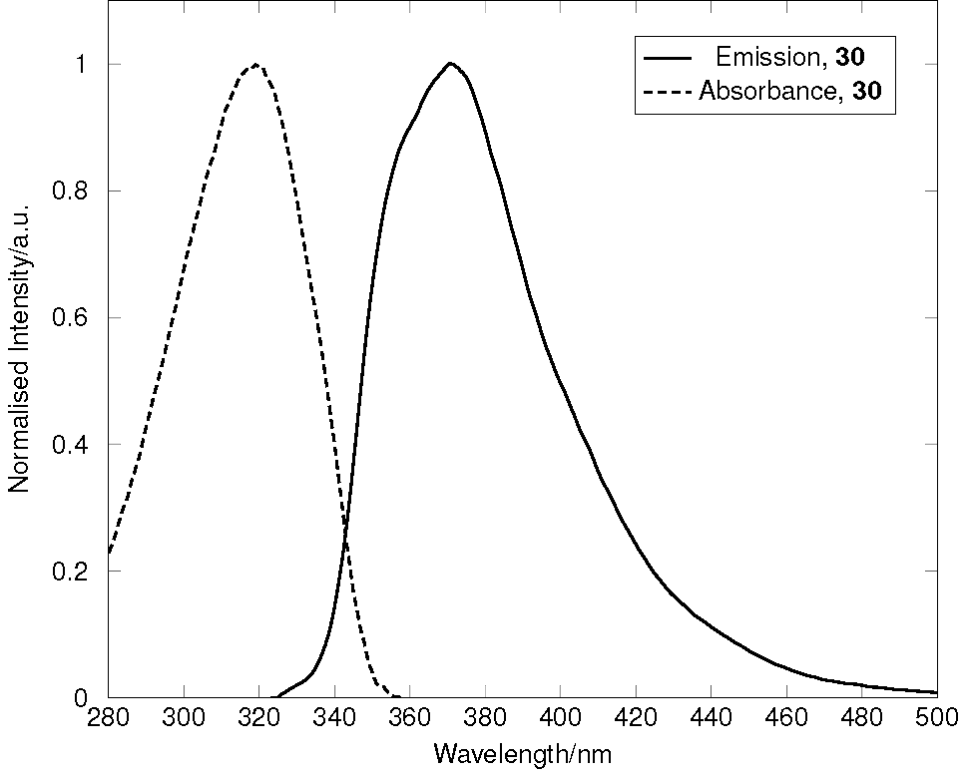
Combined, normalised absorption and emission spectra of **30** in diethyl ether. Absorption spectrum was recorded at 10 μM. Emission spectrum was recorded at 100 nM, with excitation at 318 nm.

Figure 5 shows the combined, normalised absorbance and emission profiles in diethyl ether at 10 μM and 100 nM, respectively. In diethyl ether, **30** exhibited a major absorbance at 318 nm, and when excited at this wavelength, exhibited an emission that peaks at 370 nm. A similar maximal absorbance was observed in chloroform (323 nm), and excitation at this wavelength produced an emission that was significantly lower in intensity, and mildly red shifted (to 376 nm). This behaviour indicates that 1,4-DHQ structures of this type may function as more effective donor chromophores in dyes and fluorophores than the corresponding THQs due to improved orbital overlap between the nitrogen lone pair and the aromatic π-system by virtue of the more planar ring structure.

## Conclusion

In conclusion, we have explored a wide range of methods for the synthesis of highly lipophilic tetrahydroquinolines and dihydroquinolines. Their iodo-substituents are reactive substrates for direct cross coupling using the plethora of cross-coupling methodologies in the literature. Iodoquinolin-2-ones **13**, **19** and **20a**, are stable intermediates, and we have developed straightforward, practical and scalable procedures towards their synthesis. We have also demonstrated the novel synthesis of dihydroquinoline **22**, by what appears to be a collapse of the intermediate DIBAL complex that is favoured at higher temperatures. Further mechanistic insight into this interesting reaction was garnered by varying the structure of the quinolin-2-one starting material, and indicated that increased steric bulk in the benzylic position and an *N*-alkyl substituent improves selectivity for the DHQ over the corresponding THQ. Comparison of the reactivity of 6-iodo-THQ **21** and 6-bromo-THQ **26** in a typical Suzuki coupling reaction showed significantly improved conversion in the case of the iodide. The resulting biaryl **28** was found to be highly fluorescent, highlighting the suitability of general structure **1** as an electron donor for the design of charge transfer fluorophores. Finally, crystallographic analysis of the boronic esters **29** and **30** highlighted a subtle flattening of the DHQ structure when compared to the saturated THQ; a structural characteristic that was found to cause fluorescence in **30**, indicating that the DHQ may be a more effective electron donor than the THQ. We are currently utilising the optimised syntheses of these interesting compounds in a range of novel fluorophores, and their applications will be communicated in due course.

## Experimental

### Synthetic chemistry

Reagents were purchased from Sigma-Aldrich, Acros Organics, Alfa-Aesar and Fluorochem and used without further purification unless otherwise stated. 4-Iodo-2-methylaniline and 4-iodoaniline were either purchased from Fluorochem or prepared according to a literature method [16]. Solvents were used as supplied, and dried before use if required with appropriate drying agents or using an Innovative Technologies Inc. Solvent Purification System. Thin-layer chromatography (TLC) was conducted using Merck Millipore silica gel 60G F254 25 glassplates and/or TLC-PET foils of aluminium oxide with fluorescent indicator 254 nm (40 × 80 mm) with visualisation by UV lamp or appropriate staining agents. Flash column chromatography was performed using SiO_2_ from Sigma-Aldrich (230–400 mesh, 40–63 μm, 60 Å), or activated neutral aluminium oxide (Alumina) from Sigma-Aldrich, and monitored using TLC. NMR spectra were recorded in CDCl_3_ using Varian VNMRS-700, Varian VNMRS-600, Bruker Avance-400 or Varian Mercury-400 spectrometers operating at ambient probe temperature. NMR peaks are reported as singlet (s), doublet (d), triplet (t), quartet (q), broad (br), septet (sept), combinations thereof, or as a multiplet (m). ESMS was performed by the Durham University departmental service using a TQD (Waters Ltd, UK) mass spectrometer with an Acquity UPLC (Waters Ltd, UK), and accurate mass measurements were obtained using a QtoF Premier mass spectrometer with an Acquity UPLC (Waters Ltd, UK). IR spectra were recorded using a Perkin Elmer FTIR spectrometer. Melting points were obtained using a Gallenkamp melting point apparatus and are uncorrected. Elemental analysis was conducted by the Durham University departmental service using an Exeter Analytical CE-440 analyser. UV–vis spectroscopy was conducted using a CARY100 UV–visible spectrophotometer using the Cary WinUV Scan software 3.00(182). Fluorescence emission spectroscopy was conducted using a Perkin Elmer LS 55 fluorescence spectrometer. All in situ FTIR spectroscopy experiments (ReactIR, Mettler Toledo) were carried out using a ReactIR 15 with MCT detector. Apodization = Happ General. Probe: Prob A DiComp (Diamond) connected via KAgX 9.5 mm × 2 m Fiber (Silver Halide); Sampling 2000–650 at 8 cm^−1^ resolution; Scan option: auto select, gain 1X.

### X-ray crystallography

Single-crystal diffraction experiments were conducted on a Bruker APEX-II CCD diffractometer (**13**,**14**, **15b**, **29** and **30**) and an Xcalibur Sapphire3 diffractomer (**19**), using Mo Kα radiation. Crystals were cooled using Cryostream (Oxford Cryosystems) open-flow N_2_ cryostats. The structures were solved within Olex2 by direct methods and refined by full-matrix least squares against F^2^ of all data, using SHELXTL software [33–37]. All non-hydrogen atoms were refined anisotropically. Hydrogen atom positions were calculated geometrically and refined using the riding model. CCDC (1433617–1433622) contains the supplementary crystallographic data for this paper. The data can be obtained free of charge from The Cambridge Crystallographic Data Centre via http://www.ccdc.cam.ac.uk/getstructures.

## Supporting Information

Supporting information features full synthetic procedures, in situ FTIR data plots, copies of ^1^H, ^13^C and ^11^B NMR spectra, and full crystallographic data including images and CIF files.

File 1 Full synthetic procedures, in situ FTIR data plots, and copies of ^1^H, ^13^C and ^11^B NMR spectra.

File 2 Crystallographic Information Files of compounds **13**, **14**, **15b**, **19**, **29**, and **30**.
