# Supplementary material for: Practical synthetic strategies towards lipophilic 6-iodotetrahydroquinolines and -dihydroquinolines
Source: Beilstein J Org Chem. 2016 Aug 16;12:1851–62. doi: 10.3762/bjoc.12.174 (PMC5082455; doi:10.3762/bjoc.12.174)
Supplement: File 1 — Full synthetic procedures, in situ FTIR data plots, and copies of 1H, 13C and 11B NMR spectra. [file Beilstein_J_Org_Chem-12-1851-s001.pdf]

Supporting Information
for

Practical synthetic strategies towards lipophilic 6-iodotetrahydroquinolines and -dihydroquinolines

David R. Chisholm[1], Garr-Layy Zhou[1], Ehmke Pohl[1,2], Roy Valentine[3] and Andrew Whiting[1*§]

Address: [1]Centre for Sustainable Chemical Processes, Department of Chemistry, Durham University, South Road, Durham, DH1 3LE, UK, [2]Biophysical Sciences Institute, Durham University, South Road, Durham, DH1 3LE, UK and [3]High Force Research Limited, Bowburn North Industrial Estate, Bowburn, Durham, DH6 5PF, UK

Email: Andrew Whiting - andy.whiting@durham.ac.uk

[§]Fax: +44 (0)191 384 4737; Tel: +44 (0)191 334 2081

*Corresponding author

Full synthetic procedures, in situ FTIR data plots, and copies of ^{1}H, ^{13}C and ^{11}B NMR spectra

1. ReactIR studies
To a rapidly stirred solution of compound **20a** (approximately 0.2 mmol) in an anhydrous solvent (10 mL) under Ar, was added dropwise DIBAL (1.0 M in cyclohexane, 1.0 equivalent) and the resultant solution stirred at rt until no further change was detected by ReactIR. The reactions were monitored by ReactIR in situ IR spectroscopy (Mettler Toledo) using the following procedure: 1) recorded a blank spectrum of the argon atmosphere, 2)

recorded a blank spectrum of the reaction solvent, 3) added the solution of compound **20a**, 4) identified the amide carbonyl stretch (1678–1685 cm^{-1}), 5) followed the amide carbonyl stretch whilst 1 equivalent of DIBAL was added using the 'Trend' function in the iC IR^{TM} (Mettler Toledo) software.

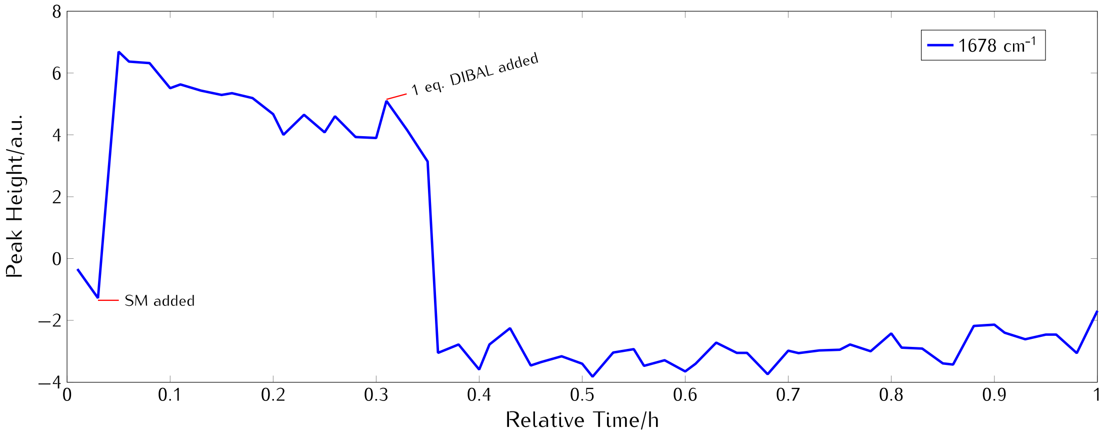

Figure S1: One equivalent of DIBAL added to a solution of 20a in anhydrous DCM at rt.

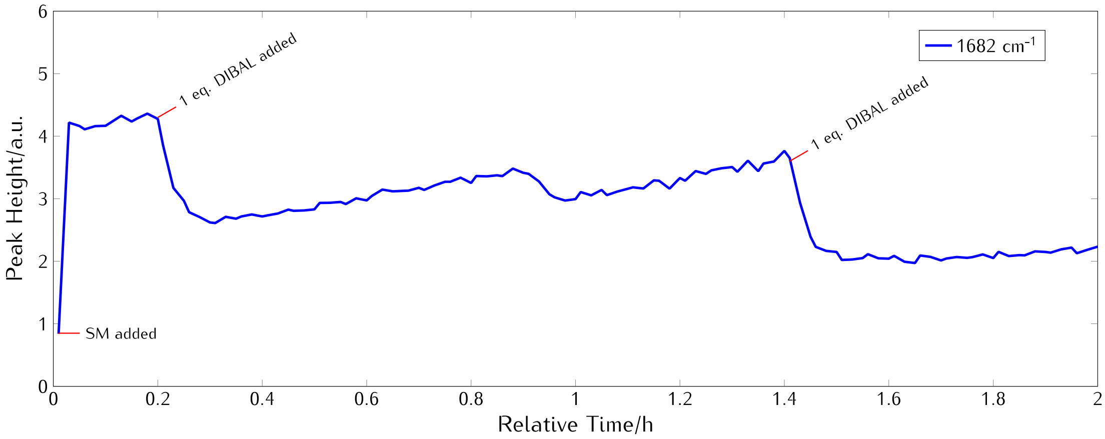

Figure S2: One equivalent of DIBAL added to a solution of 20a in anhydrous THF at rt. A further equivalent of DIBAL was added after 1.4 hours after the progress of the reaction appeared to be slow.

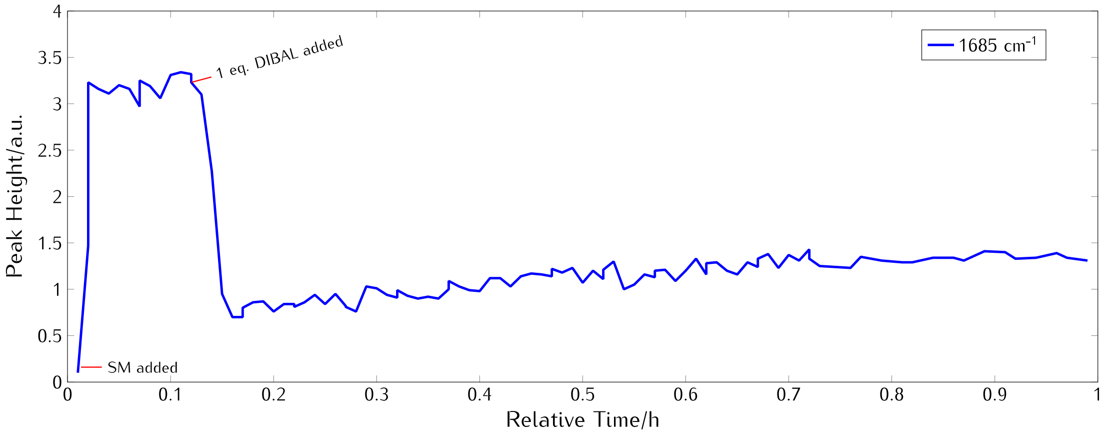

Figure S3: One equivalent of DIBAL added to a solution of 20a in anhydrous toluene at rt.

2. DIBAL reductions of quinolin-2-ones, 23a–e

Reductions of quinolin-2-ones, **23a-e** were conducted according to the optimised protocol developed for the reduction of **20a** to give DHQ **22**. The reactions were halted when TLC analysis indicated completion. In cases where the DHQ was formed in addition to the corresponding THQ (**23b** and **23d**), isolating the individual species by SiO_2 or neutral Al_2O_3 chromatography was found to be very difficult. Accordingly, the crude ^{1}H NMR spectra of reactions were analysed in order to ascertain the approximate ratios of each product formed. Approximate yields of each species were calculated by comparing the relative integrals of corresponding signals in the NMR spectra.

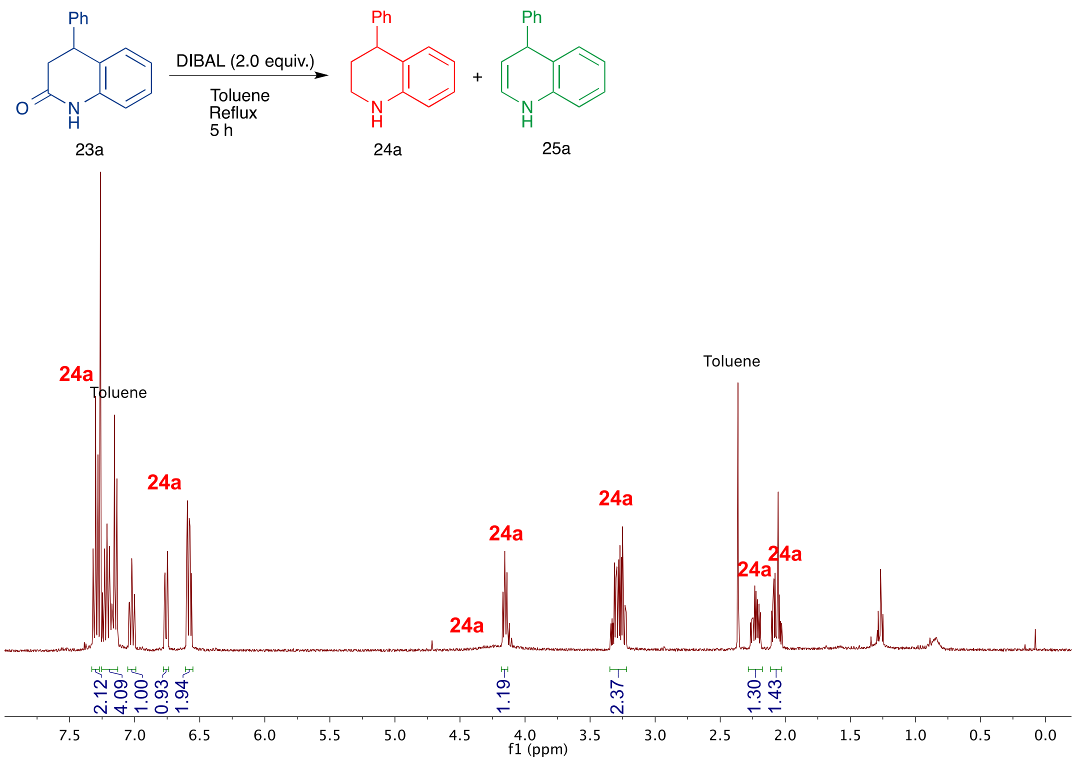

Figure S4: ^{1}H NMR spectrum of the crude mixture of the reaction between DIBAL and 23a. Only the THQ 24a was present, along with residual toluene and EtOAc [1].

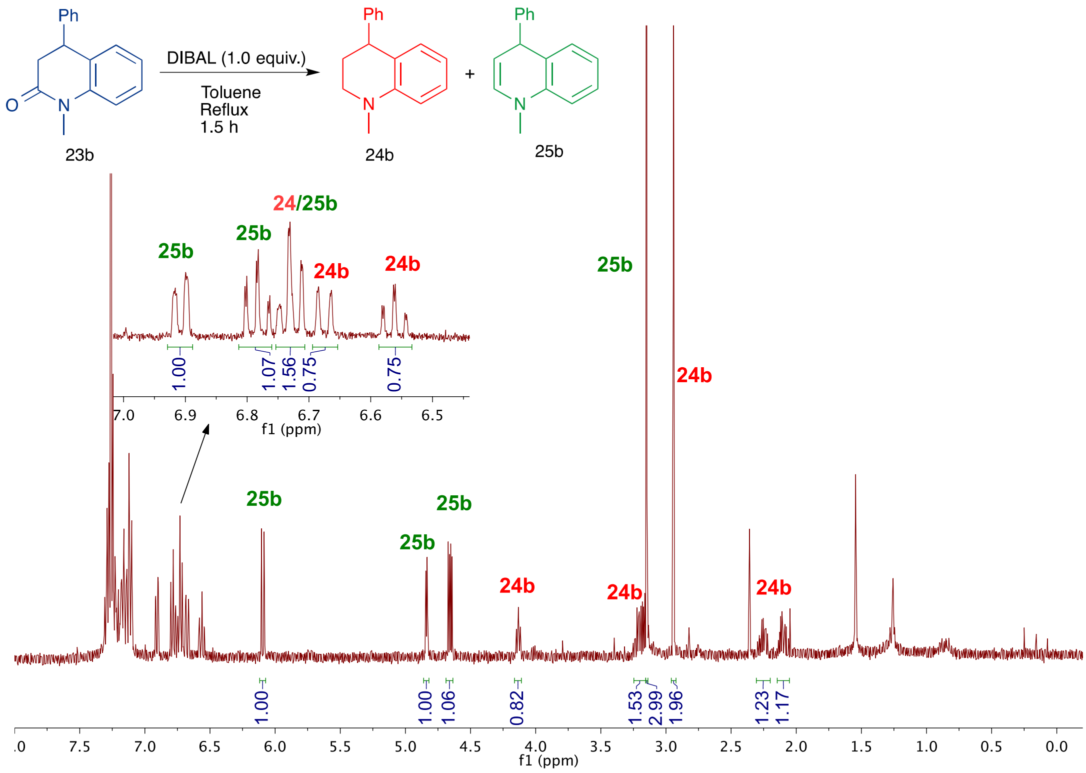

Figure S5: ¹H NMR spectrum of the crude mixture of the reaction between DIBAL and 23b. Approximate yields of THQ 24b and DHQ 25b were calculated by comparing the integrals of the corresponding *N*-Me signals (2.94 and 3.14 ppm, respectively) [2].

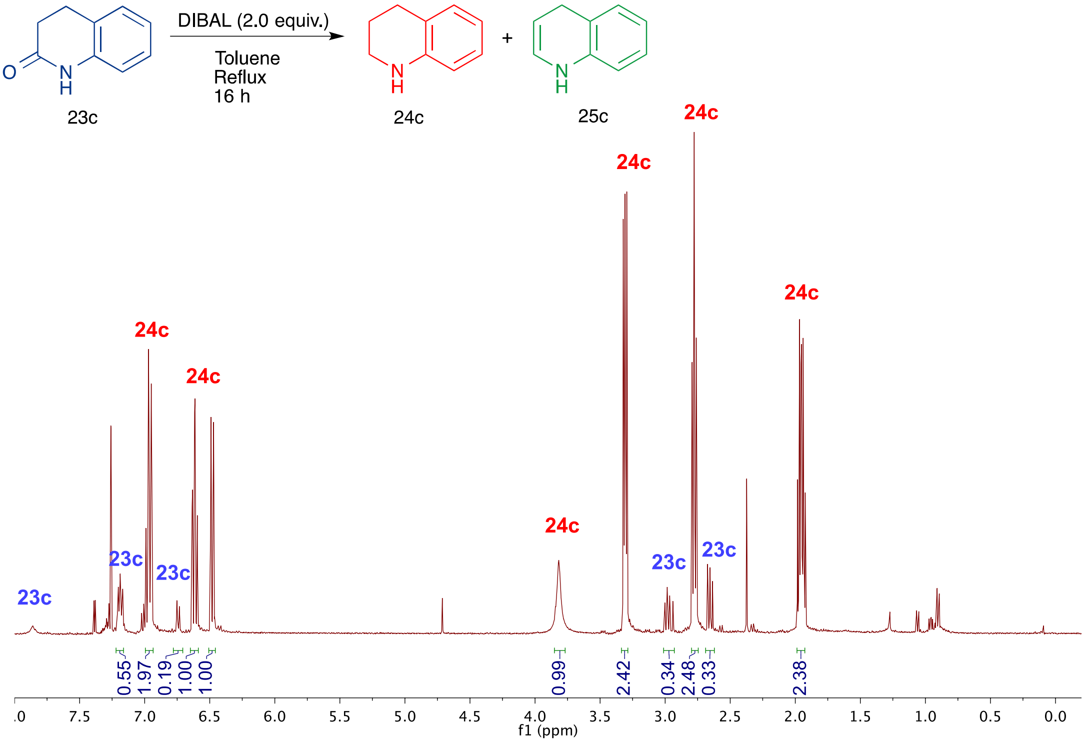

Figure S6: ¹H NMR spectrum of the crude mixture of the reaction between DIBAL and 23c. The approximate yield of THQ 24c compared to remaining 23c was calculated by comparing the integrals of the corresponding benzylic signals (2.77 and 2.97 ppm, respectively) [3].

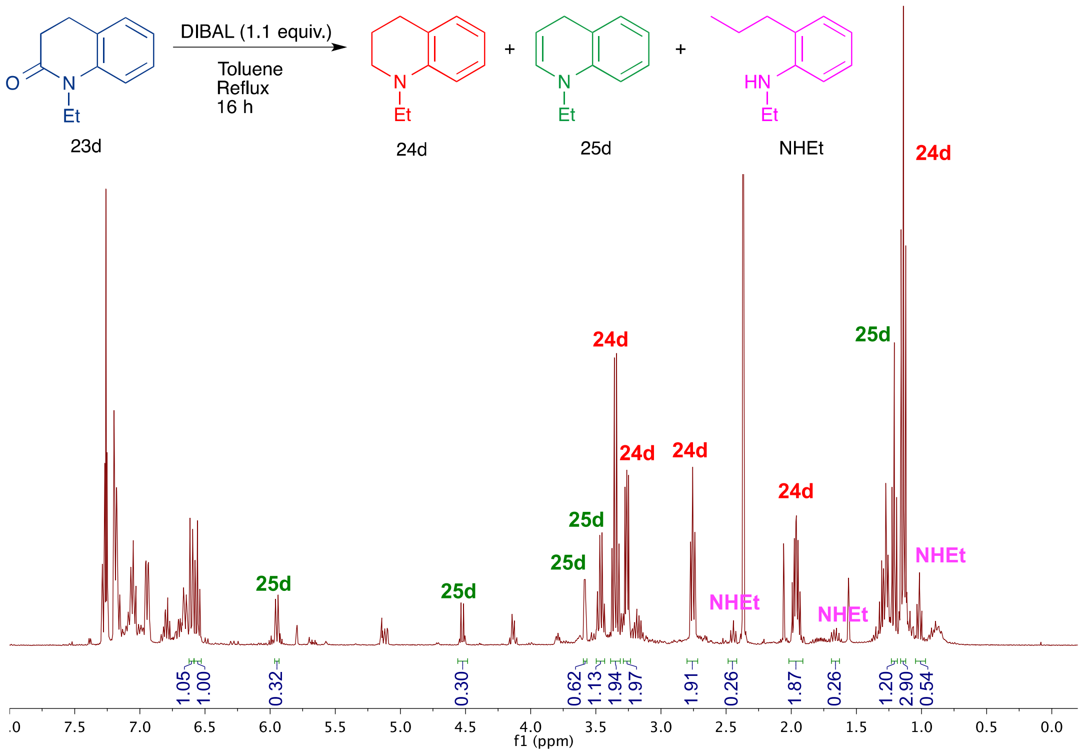

Figure S7: ^{1}H NMR spectrum of the crude mixture of the reaction between DIBAL and 23d. Approximate yields of THQ 24d, DHQ 25d and ring-opened aniline NHEt were calculated by comparing the integrals of the corresponding benzylic signals (2.76, 3.58 and 2.44 ppm, respectively). Other unidentified byproducts were also included in the calculation.

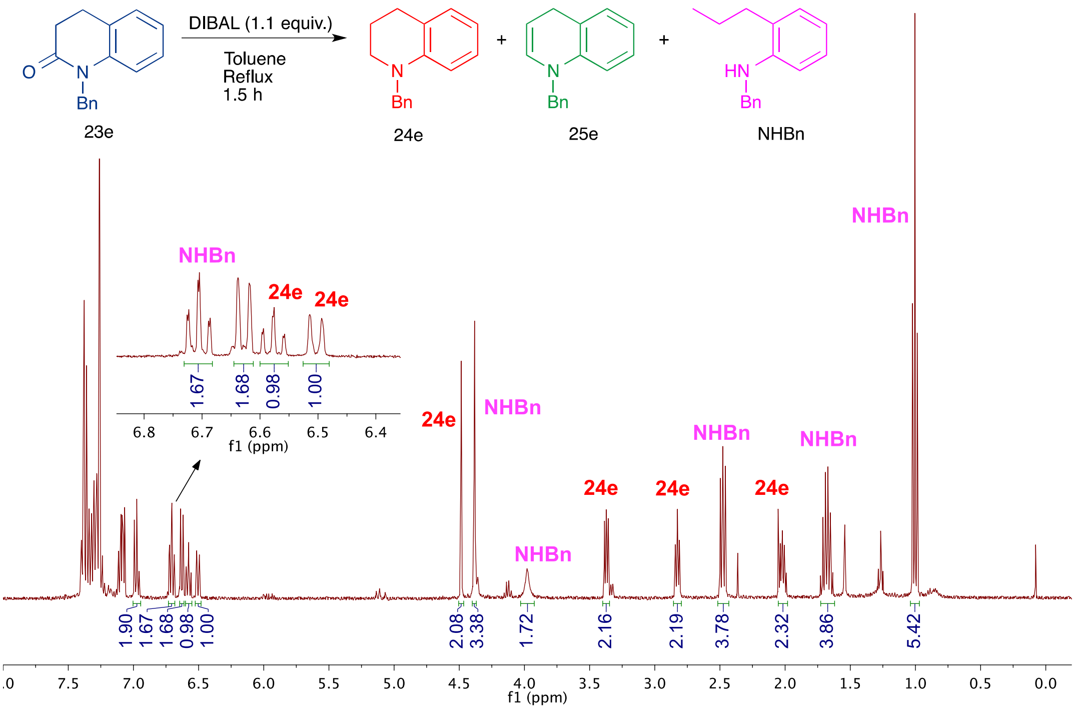

Figure S8: ^{1}H NMR spectrum of the crude mixture of the reaction between DIBAL and 23e. Approximate yields of THQ 24e and ring-opened aniline NHBn were calculated by comparing the integrals of the corresponding benzylic signals (2.82 and 2.46 ppm respectively). Trace amounts of 25e were also compared [4].

3. Synthetic procedures

2-Methyl-*N*-(propan-2-yl)aniline (6)

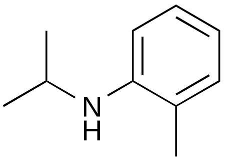

2-Iodopropane (40 mL, 400 mmol) was added to a refluxing mixture (105 °C) of *o*-toluidine (**5**, 21.4 mL, 200 mmol) and powdered NaOH (20 g, 500 mmol). The resultant solution was stirred at reflux for 12 h under Ar. The mixture was filtered, diluted with Et_2O, washed with H_2O, dried ($MgSO_4$) and evaporated. The residue purified by basic Al_2O_3 chromatography (hexane:EtOAc 95:5 as eluent), to afford compound **6** as a yellow oil (24.6 g, 82%): ^{1}H NMR (600 MHz; $CDCl_3$) δ 1.28 (d, *J* = 6.3 Hz, 6H), 2.15 (s, 3H), 3.33 (br, 1H), 3.71 (sept, *J* = 6.3 Hz, 1H), 6.64-6.67 (m, 2H), 7.08 (d, *J* = 7.5 Hz, 1H), 7.18 (t, *J* = 8.1 Hz, 1H); ^{13}C NMR (176 MHz; $CDCl_3$) δ 17.8, 23.4, 44.2, 110.4, 116.6, 121.9, 127.3, 130.4, 145.6; IR (neat) ν_{max}/cm^{-1} 3432br, 3040w, 2960m, 2862w, 1605s, 1508s, 1476m, 1382m, 741s; MS (ES): *m/z* = 150.1 $[M+H]^+$; HRMS (ES) calcd. for $C_{10}H_{16}N$ $[M+H]^+$: 150.1283, found: 150.1277.

2-Methyl-*N*-(3-methylbut-2-en-1-yl)-*N*-(propan-2-yl)aniline (4)

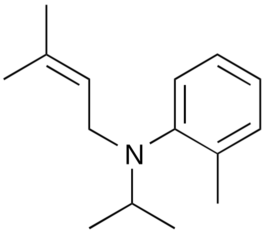

3,3-Dimethylallyl bromide (9.7 mL, 84 mmol) and compound **6** (11.3 g, 76 mmol) were dissolved in MeCN (200 mL). K_2CO_3 (11.6 g, 84 mmol) was added and the flask stirred at reflux overnight under Ar. The mixture was filtered and the solvent removed in vacuo to give a dark residue that was purified by basic Al_2O_3 chromatography, to give **4** as a yellow oil (11.5 g, 70%): ^{1}H NMR (700 MHz; $CDCl_3$) δ 1.10 (s, 6H), 1.60 (d, *J* = 7.0 Hz, 6H) 2.31 (s, 3H), 3.22 (sept, *J* = 7.0 Hz, 1H), 3.60 (d, *J* = 6.3 Hz, 2H), 5.02 (t, *J* = 6.3 Hz, 1H), 6.96 (t, *J* = 7.7 Hz, 1H), 7.04 (d, *J* = 7.7 Hz, 1H), 7.11 (t, *J* = 7.7 Hz, 1H), 7.19 (d, *J* = 7.7 Hz, 1H); ^{13}C NMR (176 MHz; $CDCl_3$) δ 18.5, 18.5**,** 19.7, 25.8, 44.2, 53.0, 123.0, 123.1, 123.7, 125.6, 130.9, 132.6, 135.7, 150.1; IR (neat) ν_{max}/cm^{-1} 2968s, 2929m, 1598m, 1490s, 1449m, 1380m, 1360m; MS (ES): *m/z* = 218 $[M+H]^+$; HRMS (ES) calcd for $C_{15}H_{24}N$ $[M+H]^+$: 218.1903, found: 218.1906.

4,4,8-Trimethyl-1-(propan-2-yl)-1,2,3,4-tetrahydroquinoline (3)

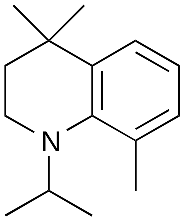

A mixture of polyphosphoric acid (PPA) (60 g) and compound **4** (10.6 g, 49 mmol) was heated to 120 °C for 24 h. The mixture was cooled, diluted with H_2O (50 mL), and 5% KOH added at 0 °C until pH 7. The reaction mixture was extracted with Et_2O (500 mL), and the extracts washed with H_2O, dried ($MgSO_4$) and evaporated to give a crude oil. This was purified by SiO_2 chromatography (hexane/EtOAc 9:1 as eluent), to afford compound **3** as a yellow oil (8.2 g, 78%): ^{1}H NMR (700 MHz; $CDCl_3$) δ 1.15 (d, *J* = 7.0 Hz, 6H) 1.28 (s, 6H), 1.68-1.65 (m, 2H), 2.26 (s, 3H), 3.12-3.15 (m, 2H), 3.55 (sept, *J* = 7.0 Hz, 1H), 6.79 (t, *J* = 7.7 Hz, 1H), 6.97 (dd, *J* = 7.0, 0.7 Hz, 1H), 7.15 (dd, *J* = 7.7, 1.4 Hz, 1H): ^{13}C NMR (176 MHz; $CDCl_3$) δ 20.5, 20.8, 32.2, 32.5, 37.8, 38.5, 53.0, 120.0, 124.1, 129.6, 130.1, 138.0, 146.0; IR (neat) ν_{max}/cm^{-1} 2959s, 1591w, 1464s, 1426s, 1130s; MS (ES$^+$): *m/z* = 218 [M+H]$^+$; HRMS (ES) calcd for $C_{15}H_{24}N$ [M+H]$^+$: 218.1903, found: 218.1904.

6-Bromo-4,4,8-trimethyl-1-(propan-2-yl)-1,2,3,4-tetrahydroquinoline (7)

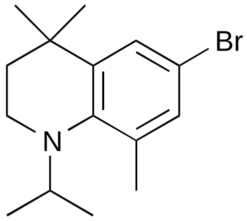

A stirred solution of compound **3** (2.30 g, 10.6 mmol) in chloroform (20 mL) was cooled to −60 °C in an acetone bath using a cryostat. Bromine (0.515 ml, 10.1 mmol) was then added dropwise to the reaction mixture. The temperature was raised gradually to −30 °C over a period of 1 h, after which the mixture was washed with a saturated solution of $NaHCO_3$ (3 × 20 mL), and the organics dried ($MgSO_4$) and evaporated. The crude oil was purified by filtering through a pad of neutral alumina (hexane/EtOAc 9:1 as eluent) to give compound **7** as a colourless oil after evaporation (1.95 g, 62%): ^{1}H NMR (600 MHz; $CDCl_3$) δ 1.13 (d, *J* = 6.6 Hz, 6H), 1.26 (s, 6H), 1.62-1.65 (m, 2H), 2.22 (s, 3H), 3.09-3.12 (m, 2H), 3.48 (sept, *J* = 6.6 Hz), 7.08 (d, *J* = 2.4 Hz, 1H), 7.21 (d, *J* = 2.4 Hz, 1H); ^{13}C NMR (151 MHz; $CDCl_3$) δ 20.3, 20.7, 31.9, 32.8, 37.3, 38.4, 53.1, 112.6, 126.8, 132.0, 132.4, 140.2, 145.2; IR (neat) ν_{max}/cm^{-1} 2960s, 1468s, 860s, 829m, 742m; MS (EI): *m/z* = 296 [M + H]$^+$; HRMS (ES) calcd for $C_{15}H_{23}NBr$ [M + H]$^+$: 296.1014, found: 296.1004.

4-[(2-Methylphenyl)amino]butan-2-one (8)

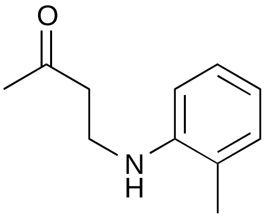

o-Toluidine, **5**, (2.68 mL, 25 mmol) and methyl vinyl ketone (2.03 mL, 25 mmol) were added to H_2O (50 mL) and the mixture was stirred vigorously for 48 h in the absence of light. The resultant product was extracted with EtOAc, washed with H_2O and brine, dried ($MgSO_4$) and evaporated to give a crude brown oil which was purified by distillation under vacuum using a Kugelrohr apparatus (approx. 0.5 Torr, **8** isolated between 80–110 °C) to give compound **8** as a colourless oil (2.26 g, 51%): ^1H NMR (700 MHz, $CDCl_3$) δ 2.14 (s, 3H), 2.18 (s, 3H), 2.79 (t, *J* = 6.2 Hz, 2H), 3.48 (t, *J* = 6.2 Hz, 2H), 3.92 (br, 1H), 6.64 (d, *J* = 8.0 Hz, 1H), 6.69 (td, *J* = 7.3, 0.9 Hz, 1H), 7.08 (d, *J* = 7.3 Hz, 1H), 7.15 (td, *J* = 7.3, 0.9 Hz, 1H); ^{13}C NMR (176 MHz, $CDCl_3$) δ 17.5, 30.4, 38.4, 42.7, 109.7, 117.3, 122.6, 127.2, 130.4, 145.8, 208.3; IR (neat) ν_{max}/cm^{-1} 3414w, 2980w, 2930w, 1708s, 1605, 1510s, 1448m, 1317m, 1263m, 1167s, 745s, 716m; MS(ES): *m/z* = 178.1 $[M+H]^+$: HRMS (ES) calcd. for $C_{11}H_{16}NO$ $[M+H]^+$: 178.1232, found 178.1239 [5].

Note: Compound **8** turns dark brown upon standing, but no obvious degradation is observed by ^1H NMR.

4,4,8-Trimethyl-1,2,3,4-tetrahydroquinoline (10)

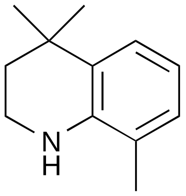

MeMgBr solution (3.0 M in Et_2O, 8.27 mL, 24.82 mmol) was added dropwise to a solution of compound **8** (2.0 g, 11.28 mmol) in dry THF (60 mL) at −78 °C under Ar. The mixture was stirred overnight, before being quenched and neutralised with 5% HCl, diluted with EtOAc, washed with H_2O and brine, dried ($MgSO_4$) and evaporated to give a crude brown oil of compound **9** (1.9 g, approx. 80% by NMR). A portion of crude **9** (1.48 g, 7.66 mmol) was dissolved in DCM (50 mL) and conc. H_2SO_4 (2 mL) added. The resultant solution was stirred at reflux for 4 h, before being cooled, neutralised with 5% NaOH (w/v), diluted with DCM, washed with H_2O, dried ($MgSO_4$) and evaporated to a give a crude brown oil. This was purified using SiO_2 chromatography (hexane/EtOAc 9:1, with 1% Et_3N as eluent) to give **10** as an orange oil (0.91 g, 68%): ^1H NMR (600 MHz, $CDCl_3$) δ 1.33 (s, 6H), 1.76-1.79 (m, 2H), 2.11 (s, 3H), 3.39-3.41 (m, 2H), 3.75 (br, 1H), 6.62 (t, *J* = 7.5 Hz, 1H), 6.90 (d, *J* = 7.3 Hz, 1H), 7.13 (d, *J* = 8.5 Hz, 1H); ^{13}C NMR (151 MHz, $CDCl_3$) δ 17.8, 31.5, 32.0, 37.5, 38.8, 116.5, 121.1, 124.5, 127.9, 129.9, 141.8; IR (neat) ν_{max}/cm^{-1} 3418br, 2964m, 2920m, 2850m, 1597m, 1472s, 1355m, 1103m, 740s; *m/z* = 176.1 $[M+H]^+$; HRMS (ES) calcd. for $C_{12}H_{18}N$ $[M+H]^+$: 176.1439, found 176.1442.

N-(4-Iodo-2-methylphenyl)-3-methylbut-2-enamide (12)

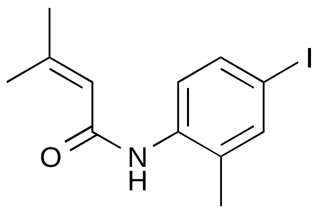

3,3-Dimethylacryloyl chloride (0.22 mL, 2.2 mmol) and pyridine (0.194 ml, 2.4 mmol) was added to a solution of 4-iodo-2-methylaniline (**11**, 0.466 g, 2.0 mmol) in DCM (30 mL). The resultant solution was stirred at rt for 6 h, and then diluted with DCM. The organics were washed with sat. NH_4Cl, H_2O and brine, dried ($MgSO_4$) and evaporated to give a crude white solid (0.60 g). This was purified by SiO_2 chromatography (hexane/EtOAc 8:2, with 1% Et_3N as eluent) to give compound **12** as a white solid (0.53 g, 84%): m.p. = 106-107 ^{o}C; ^{1}H NMR (400 MHz; $CDCl_3$) δ 1.90 (s, 3H), 2.19 (s, 3H), 2.20 (d, J = 1.2 Hz, 3H), 5.72 (br, 1H), 6.86 (br, 1H), 7.47-7.51 (m, 2H), 7.69 (br, 1H); ^{13}C NMR (101 MHz; $CDCl_3$) δ 17.6, 20.2, 27.6, 88.6, 118.4, 124.4, 130.8, 135.8, 136.1, 139.1, 154.3, 165.1; IR (neat) ν_{max}/cm^{-1} 3293w, 2980w, 2940w, 1635s, 1508s, 1442m, 1390m, 1286s, 838s, 798m, 620w; MS(ES): m/z = 316.0 $[M+H]^+$; HRMS (ES) calcd. for $C_{12}H_{15}INO$ $[M+H]^+$: 316.0198, found 316.0200; Found: C, 45.76; H, 4.46; N 4.36. Calc. for $C_{12}H_{14}INO$: C, 45.73; H, 4.48; N 4.44%.

6-Iodo-4,4,8-trimethyl-1,2,3,4-tetrahydroquinolin-2-one (13)

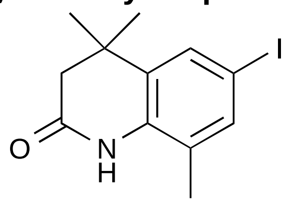

To a solution of compound **12** (0.13 g, 0.41 mmol) in DCM (15 mL) was added conc. H_2SO_4 (1 mL), and the solution was stirred at reflux overnight. The resultant solution was neutralised with sat. K_2CO_3 solution and diluted with DCM. The organics were washed with H_2O and with brine, dried ($MgSO_4$) and evaporated to give a crude orange solid (0.2 g). This was purified by SiO_2 chromatography (hexane/EtOAc 8:2, with 1% Et_3N as eluent) to give compound **13** as a white solid (0.088 g, 67%): m.p. = 184-186 ^{o}C; ^{1}H NMR (700 MHz; $CDCl_3$) δ 1.30 (s, 6H), 2.21 (s, 3H), 2.45 (s, 2H), 7.38 (d, J = 2.0 Hz, 1H), 7.43 (d, J = 2.0 Hz, 1H), 7.80 (br, 1H); ^{13}C NMR (176 MHz; $CDCl_3$) δ 17.0, 27.7, 34.3, 45.1, 86.6, 125.7, 131.6, 134.4, 135.0, 137.8, 170.4; IR (neat) ν_{max}/cm^{-1} 3189w, 2948w, 2926w, 1677s, 1474m, 1444m, 1361m, 1252m, 866s, 744m, 668w; MS(ES): m/z = 316.0 $[M+H]^+$; HRMS (ES) calcd. for $C_{12}H_{15}NOI$ $[M+H]^+$: 316.0198, found 316.0197; Found: C, 45.65; H, 4.48; N 4.42. Calc. for $C_{12}H_{14}NOI$: C, 45.73; H, 4.48; N 4.44%.

6-Iodo-4,4,8-trimethyl-2-(propan-2-yloxy)-3,4-dihydroquinoline (15b)

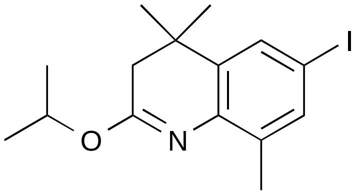

Compound **13** (0.8 g, 2.54 mmol) and NaH (60% dispersion in mineral oil, 0.15 g, 3.5 mmol) were added to a Schlenk flask. This was purged of air under vacuum and refilled with Ar, and anhydrous DMF (12 mL) was added. The resultant solution was heated at 80 °C for 1 h under Ar. The mixture was cooled, 2-iodopropane (0.51 mL, 5.08 mmol) was added and the mixture was stirred at 80 °C overnight. The resultant pale yellow solution was cooled and H_2O and EtOAc added. The organics were washed with sat. NH_4Cl, H_2O and brine, dried ($MgSO_4$) and evaporated to give a crude clear oil (1.0 g). This was purified using SiO_2 chromatography (hexane/EtOAc 8:2, with 1% Et_3N as eluent) to give compound **15b** as a colourless solid (0.30 g, 33%): m.p. = 85-87 ^oC; ^1H NMR (700 MHz; $CDCl_3$) δ 1.21 (s, 6H), 1.33 (d, *J* = 6.0 Hz, 6H), 2.19 (s, 2H), 2.30 (s, 3H), 5.39 (sept, *J* = 6.0 Hz, 1H), 7.38-7.40 (m, 2H); ^{13}C NMR (176 MHz; $CDCl_3$) δ 17.7, 22.0, 27.7, 33.9, 40.1, 68.5, 88.2, 130.2, 135.8, 137.5, 138.1, 141.7, 164.7; IR (neat) ν_{max}/cm^{-1} 2965w, 2916w, 2872w, 1617s, 1563m, 1372s, 1235s, 897s; MS(ES): *m/z* = 358.0 $[M+H]^+$; HRMS (ES) calcd. for $C_{15}H_{21}NOI$ $[M+H]^+$: 358.0668, found 358.0660.

6-Iodo-4,4,8-trimethyl-1,2,3,4-tetrahydroquinoline (14)

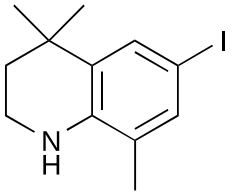

To a solution of compound **13** (0.4 g, 1.27 mmol) in dry toluene (20 mL) was added borane·dimethyl sulphide complex (0.13 mL, 1.35 mmol), and the solution was stirred at reflux overnight under Ar. The solution was then cooled, 10% aq Na_2CO_3 added (10 mL), and then stirred for 0.5 h. The solution was then diluted with EtOAc, washed with H_2O and brine, dried ($MgSO_4$) and evaporated to give a crude oil (0.33 g). This was purified by SiO_2 chromatography (hexane/EtOAc 8:2 with 1% Et_3N as eluent) to give compound **14** as a white solid (0.30 g, 79%): m.p. = 84-86 ^oC; ^1H NMR (700 MHz; $CDCl_3$) δ 1.28 (s, 6H), 1.70-172 (m, 2H), 2.03 (s, 3H), 3.35-3.37 (m, 2H), 3.75 (br, 1H), 7.16 (d, *J* = 2.0 Hz, 1H), 7.33 (d, *J* = 2.0 Hz, 1H); ^{13}C NMR (176 MHz; $CDCl_3$) δ 17.3, 31.2, 32.1, 36.9, 38.6, 77.5, 123.7, 132.4, 133.1, 136.1, 141.4; IR (neat) ν_{max}/cm^{-1} 3441w, 2960w, 2926w, 1581m, 1496s, 1442m, 1350m, 1280s, 859s, 746m, 604w; MS(ES): *m/z* = 302.0 $[M+H]^+$; HRMS (ES) calcd. for $C_{12}H_{17}NI$ $[M+H]^+$: 302.0406, found 302.0404.

Note: Compound **14** slowly turns from white to red/brown on standing, with some decomposition when stored for prolonged periods.

6-Iodo-1,4,4,8-tetramethyl-1,2,3,4-tetrahydroquinoline (16)

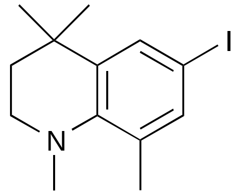

To a solution of compound **14** (0.367 g, 1.22 mmol) in anhydrous DMF (15 mL), was added NaH (60% dispersion in mineral oil, 0.060 g, 1.4 mmol) and the resultant slurry was stirred for 2 h at 80 °C under Ar. The solution was cooled, iodomethane (0.174 mL, 2.8 mmol) added, and then stirred overnight at rt. The solution was quenched with H_2O and extracted with EtOAc. The organics were washed with sat. NH_4Cl, H_2O and brine, dried ($MgSO_4$) and evaporated to give a crude orange oil (0.39 g). This was purified by SiO_2 chromatography (hexane/EtOAc 9:1, with 1% Et_3N as eluent) to give compound **16** as a clear oil (0.165 g, 43%): ^{1}H NMR (700 MHz; $CDCl_3$) δ 1.28 (s, 6H), 1.64-1.71 (m, 2H), 2.24 (s, 3H), 2.69 (s, 3H), 3.07-3.10 (m, 2H), 7.28-7.30 (m, 1H), 7.42 (d, *J* = 2.0 Hz, 1H); ^{13}C NMR (176 MHz; $CDCl_3$) δ 18.6, 31.7, 32.7, 33.0, 42.6, 48.4, 85.1, 133.5, 133.9, 137.3, 141.0, 146.8; IR (neat) ν_{max}/cm^{-1} 2959m, 2928m, 2890m, 1470s, 1142s, 860s, 733m, 653m; MS(ES): *m/z* = 316.0 $[M+H]^+$; HRMS (ES) calcd. for $C_{13}H_{19}NI$ $[M+H]^+$: 316.0562, found 316.0566.

***N*-(4-Iodophenyl)-3-methylbut-2-enamide (18)**

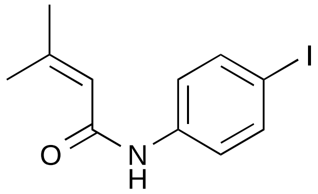

To a solution of 4-iodoaniline (**17**, 25.0 g, 114.0 mmol) in DCM (400 mL) was added 3,3-dimethylacryloyl chloride (13.36 mL, 120.0 mmol) and the resultant white suspension was stirred for 0.5 h, after which pyridine (9.70 mL, 120 mmol) was added and the solution stirred at rt for 16 h. The solution was diluted with DCM and H_2O, washed with sat. NH_4Cl, H_2O and brine, dried ($MgSO_4$) and evaporated to give a crude light brown solid (33 g) which was recrystallised from EtOH to give **18** as a white crystalline solid (31.8 g, 93%): m.p. = 136-138 ^{o}C; ^{1}H NMR (700 MHz, $CDCl_3$) δ 1.91 (s, 3H), 2.22 (s, 3H), 5.68 (s, 1H), 7.01 (s, 1H), 7.33 (m, 2H), 7.60 (d, *J* = 8.8 Hz, 2H); ^{13}C NMR (101 MHz, $CDCl_3$) δ 20.2, 27.6, 87.2, 118.7, 122.0, 137.8, 138.2, 154.1, 165.5; IR (neat) ν_{max}/cm^{-1} 3294m, 3094, 2964w, 2890w, 1666m, 1586m, 1430m, 821s, 650m; MS (ES): *m/z* = 302.0 $[M+H]^+$; HRMS (ES) calcd. for $C_{11}H_{13}NOI$ $[M+H]^+$: 302.0042, found: 302.0050; Found: C, 43.87; H, 4.02; N 4.64. Calc. for $C_{11}H_{12}NOI$: C, 43.88; H, 4.02; N 4.65%.

6-Iodo-4,4-dimethyl-1,2,3,4-tetrahydroquinolin-2-one (19)

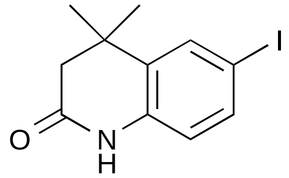

Compound **18** (11.5 g, 38.3 mmol) and $AlCl_3$ (7.66 g, 57.5 mmol) were added to anhydrous DCM (150 mL) under Ar and the resultant solution was stirred vigorously for 2.5 h at rt. The reaction was cooled to 0 °C, quenched slowly with H_2O, diluted with DCM, stirred with 5% NaOH (w/v) until the solution turned off-white, then further washed with H_2O and brine, dried ($MgSO_4$) and evaporated to give a crude yellow solid (12.0 g). This was recrystallised from EtOH to give **19** as a white crystalline solid (10.2 g, 88%): m.p. = 199-202 ^{o}C; ^{1}H NMR (700 MHz, $CDCl_3$) δ 1.32 (s, 6H), 2.47 (s, 2H), 6.62 (d, *J* = 8.3 Hz, 1H), 7.47 (dd, *J* = 8.3, 1.9 Hz, 1H), 7.56 (d, *J* = 1.8 Hz, 1H), 9.20 (s, 1H); ^{13}C NMR (176 MHz, $CDCl_3$) δ 27.7, 34.2, 45.2, 86.8, 118.1, 133.7, 135.1, 135.9, 136.6, 171.3; IR (neat) ν_{max}/cm^{-1} 3164m, 3102, 3040w, 2953m, 1671s, 1596m, 1484m, 817s; MS (ES): *m/z* = 302.0 [M+H]$^+$; HRMS (ES) calcd. for $C_{11}H_{13}NOI$ [M+H]$^+$: 302.0042, found: 302.0042; Found: C, 43.91; H, 4.02; N 4.63. Calc. for $C_{11}H_{12}NOI$: C, 43.88; H, 4.02; N 4.65%.

6-Iodo-4,4-dimethyl-1-(propan-2-yl)-1,2,3,4-tetrahydroquinolin-2-one (20a)

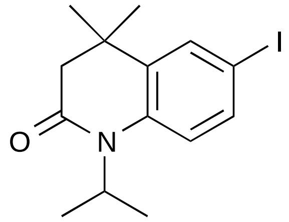

To a solution of compound **19** (25.9 g, 85.9 mmol) in anhydrous DMF (200 mL) was added crushed KOH (14.5 g, 257 mmol) and the resultant slurry was stirred for 1 h at 50 °C under Ar. To this was added 2-iodopropane (25.6 mL, 257 mmol) and the solution stirred at 50 °C for 40 h under Ar. The reaction was quenched with H_2O, diluted with EtOAc, washed with sat. NH_4Cl, H_2O and brine, dried ($MgSO_4$) and evaporated to give a crude clear oil (29.0 g). This was purified by SiO_2 chromatography (hexane/EtOAc 9:1, with 1% Et_3N as eluent) to give compound **20a** as a colourless oil (14.8 g, 50%): R_f 0.51 (hexane/EtOAc 8:2, with 1% Et_3N); ^{1}H NMR (400 MHz, $CDCl_3$) δ 1.25 (s, 6H), 1.50 (d, *J* = 7.0 Hz, 6H), 2.38 (s, 2H), 4.66 (sept, *J* = 7.0 Hz, 1H), 6.87 (d, *J* = 8.6 Hz, 1H), 7.50 (dd, *J* = 8.6, 2.1 Hz, 1H), 7.52 (d, *J* = 2.1 Hz, 1H); ^{13}C NMR (101 MHz, $CDCl_3$) δ 20.3, 26.8, 33.1, 47.2, 48.8, 86.9, 119.0, 133.4, 135.9, 139.1, 139.3, 169.8; IR (neat) ν_{max}/cm^{-1} 2961m, 2934w, 2870w, 1667s, 1582m, 1482m, 809s; MS (ES): *m/z* = 344.0 [M+H]$^+$; HRMS (ES) calcd. for $C_{14}H_{19}NOI$ [M+H]$^+$: 344.0511, found: 344.0512; Found: C, 49.21; H, 5.29; N 4.08. Calc. for $C_{14}H_{18}NOI$: C, 48.99; H, 5.29; N 4.08%.

Note: Compound **20a** was found to slowly solidify on standing over a number of weeks.

6-Iodo-4,4-dimethyl-2-(propan-2-yloxy)-3,4-dihydroquinoline (20b)

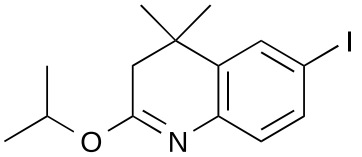

Compound **20b** was isolated as a byproduct of the alkylation of **19**, by SiO_2 chromatography (hexane/EtOAc 9:1, with 1% Et_3N as eluent) to give compound **20b** as a colourless oil (7.6 g, 26%): R_f 0.71 (hexane/EtOAc 8:2, with 1% Et_3N); ^1H NMR (700 MHz, $CDCl_3$) δ 1.23 (s, 6H), 1.32 (d, J = 7.0 Hz, 6H), 2.21 (s, 2H), 5.40 (sept, J = 6.2 Hz, 1H), 6.88 (d, J = 8.2 Hz, 1H), 7.50 (dd, J = 8.2, 2.0 Hz, 1H), 7.53 (d, J = 2.0 Hz, 1H); ^{13}C NMR (176 MHz, $CDCl_3$) δ 22.1, 27.6, 33.7, 40.3, 68.4, 88.5, 127.3, 132.6, 136.4, 138.5, 143.6, 166.2; IR (neat) ν_{max}/cm^{-1} 2960m, 2934w, 2872w, 1625s, 1586m, 1469m, 824s; MS (ES): m/z = 344.0 $[M+H]^+$; HRMS (ES) calcd. for $C_{14}H_{19}NOI$ $[M+H]^+$: 344.0511, found: 344.0513.

Note: Compound **20b** was found to slowly solidify on standing over a number of weeks.

6-Iodo-4,4-dimethyl-1-(propan-2-yl)-1,2,3,4-tetrahydroquinoline (21)

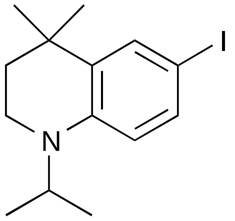

To a solution of compound **20a** (1.25 g, 3.63 mmol) in anhydrous toluene (15 mL) was added borane·dimethyl sulfide complex (2.0 M in THF, 1.91 mL, 3.81 mmol) dropwise and the resultant solution stirred at reflux for 16 h under Ar. The solution was cooled to rt, 10% aq Na_2CO_3 (25 mL) added and then stirred for 0.5 h. The resultant solution was diluted with EtOAc, washed with H_2O and brine, dried ($MgSO_4$) and evaporated to give a crude colourless oil (1.12 g). This was purified by SiO_2 chromatography (hexane/EtOAc 9:1, with 1% Et_3N as eluent) to give compound **21** as a colourless oil (1.08 g, 90%): ^1H NMR (700 MHz, $CDCl_3$) δ 1.19 (d, J = 6.6 Hz, 6H), 1.24 (s, 6H), 1.65-1.67 (m, 2H), 3.14-3.17 (m, 2H), 4.06 (sept, J = 6.6 Hz, 1H), 6.46 (d, J = 8.8 Hz, 1H), 7.28 (dd, J = 8.9, 2.1 Hz, 1H), 7.39 (d, J = 2.2 Hz, 1H); ^{13}C NMR (176 MHz, $CDCl_3$) δ 18.9, 30.3, 32.4, 36.6, 36.8, 47.3, 76.1, 113.4, 134.5, 134.8, 135.6, 144.0; IR (neat) ν_{max}/cm^{-1} 2957m, 2927w, 2863w, 1580m, 1489m, 792s, 684w; MS (ES): m/z = 330.1 $[M+H]^+$; HRMS (ES) calcd. for $C_{14}H_{21}NI$ $[M+H]^+$: 330.0719, found: 330.0717.

Note: Compound **21** slowly turns a red/pink colour when stored for prolonged periods, and ideally should be derivatised further as soon as possible.

6-Iodo-4,4-dimethyl-1-(propan-2-yl)-1,4-dihydroquinoline (22)

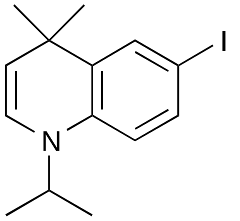

A solution of compound **20a** (0.95 g, 2.77 mmol) in anhydrous toluene (24 mL) was heated to reflux under Ar. DIBAL (1.0 M in cyclohexane) (3.05 mL, 3.05 mmol) was then added dropwise, and the resultant solution stirred rapidly for 1 h. The reaction mixture was then carefully quenched with 20% aq. NaOH (w/v), cooled, diluted with EtOAc, washed with H_2O and brine, dried ($MgSO_4$) and evaporated to give a crude colourless oil. This was purified by neutral Al_2O_3 chromatography (hexane/DCM 98:2 as eluent) to give compound **22** as a colourless oil (0.60 g, 66%); ^{1}H NMR (400 MHz, $CDCl_3$) δ 1.26 (d, *J* = 6.4 Hz, 6H), 1.27 (s, 6H), 4.12 (sept, *J* = 6.6 Hz, 1H), 4.53 (d, *J* = 7.9 Hz, 1H), 6.15 (d, *J* = 7.9 Hz, 1H), 6.56 (d, *J* = 9.1 Hz, 1H), 7.36 (dd, *J* = 8.8, 2.2 Hz, 1H), 7.45 (d, *J* = 2.2 Hz, 1H); ^{13}C NMR (101 MHz, $CDCl_3$) δ 20.8, 32.8, 33.3, 47.0, 81.8, 109.8, 113.5, 122.9, 134.6, 135.1, 135.2, 139.7; IR (neat) ν_{max}/cm^{-1} 2961w, 2925w, 2853w, 1668m, 1581m, 1478s, 1418m, 1206s, 1054m, 796s; MS (ES): *m/z* = 328.1 $[M+H]^+$; HRMS (ES) calcd. for $C_{14}H_{19}NI$ $[M+H]^+$: 328.0554, found: 328.0562.

Note: Compound **22** slowly turns a red/pink colour when stored for prolonged periods, and ideally should be derivatised further as soon as possible.

4-Phenyl-1,2,3,4-tetrahydroquinolin-2-one (23a)

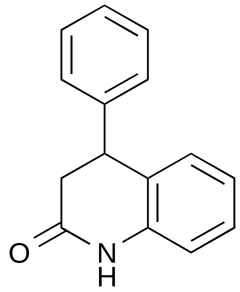

To a solution of aniline (5.87 g, 54.8 mmol) in DCM (200 mL) was added cinnamoyl chloride (9.58 g, 57.5 mmol). The resultant suspension was stirred for 0.5 h, after which pyridine (4.65 mL, 57.5 mmol) was added and the solution stirred at rt for 4 h. The solution was diluted with DCM, washed with sat. NH_4Cl, and H_2O, dried ($MgSO_4$) and evaporated to give a crude light brown solid. This was recrystallised from EtOH to give (2*E*)-*N*,3-diphenylprop-2-enamide as a colourless crystalline solid (12.08 g, 92%). Polyphosphoric acid (20 g) was heated to 120 °C, whereupon (2*E*)-*N*,3-diphenylprop-2-enamide (1 g, 4.48 mmol) was added, and the mixture stirred for 10 minutes. This was cooled, before crushed ice was added, and the resultant slurry stirred rapidly for 20 minutes. The mixture was then extracted with $CHCl_3$, washed with H_2O, dried ($MgSO_4$) and evaporated to give a crude orange solid. This was recrystallised from EtOH/H_2O (1:1) to give compound **23a** as a white solid (0.58 g, 58%): ^{1}H NMR (700 MHz; $CDCl_3$) δ 2.89-2.99 (m, 2H), 4.31 (t, *J* = 8.5 Hz, 1H), 6.86 (d, *J* = 7.4 Hz, 1H), 6.92 (d, *J* = 7.5 Hz, 1H), 6.97

(td, J = 7.5, 1.2 Hz, 1H), 7.19-7.22 (m, 3H), 7.26–7.30 (m, 1H), 7.34 (t, J = 7.6 Hz, 2H), 8.53 (br, 1H, H7); Found: C, 80.85; H, 5.88; N, 6.21. Calc. for $C_{15}H_{13}NO$: C, 80.69; H, 5.87; N, 6.27%; all other spectroscopic and analytical data were identical to those reported in the literature [6,7].

Note: Compound **23a** can also be purified via Kugelrohr distillation (190–210 °C, 0.8 Torr).

1-Methyl-4-phenyl-1,2,3,4-tetrahydroquinolin-2-one (23b)

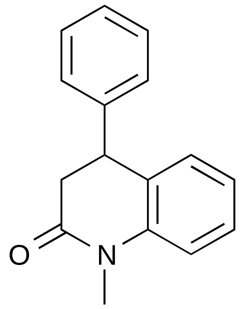

A solution of compound **23a** (0.30 g, 1.34 mmol) in anhydrous DMF (8 mL) was cooled to 0 °C under Ar, whereupon NaH (60% dispersion in mineral oil, 0.089 g, 2.02 mmol) was added, and the resultant slurry was stirred for 1 h. Iodomethane (0.1 mL, 1.61 mmol) was added, and the solution was stirred at rt for 40 h. The solution was diluted with H_2O, and extracted with EtOAc. The organics were washed with sat. NH_4Cl, H_2O and brine, dried ($MgSO_4$) and evaporated to give a crude colourless oil. This was purified by SiO_2 chromatography (hexane/EtOAc 9:1, with 1% Et_3N as eluent) to give compound **23b** as a white solid (0.98 g, 97%); m.p. 108-110 °C; ^1H NMR (700 MHz; $CDCl_3$) δ 2.92–3.02 (m, 2H), 3.40 (s, 3H), 4.21–4.26 (m, 1H), 6.93 (d, J = 7.5 Hz, 1H), 7.00 (t, J = 7.5 Hz, 1H), 7.06 (d, J = 8.1 Hz, 1H), 7.17 (d, J = 7.4 Hz, 2H), 7.27 (d, J = 7.4 Hz, 1H), 7.30 (t, J = 8.3 Hz, 1H), 7.34 (t, J = 7.4 Hz, 2H); ^{13}C NMR (176 MHz, $CDCl_3$) δ 29.7, 39.0, 41.7, 115.1, 123.2, 127.4, 128.0, 128.1, 128.2, 129.0, 129.3, 140.6, 141.2, 169.5; IR (neat) ν_{max}/cm^{-1} 3062w, 3039, 2977w, 1663s, 1596s, 1498m, 1457m, 1370s, 1275m, 759s; MS (ES): m/z = 238.1 $[M+H]^+$; HRMS (ES) calcd. for $C_{16}H_{16}NO$ $[M+H]^+$: 238.1232, found 238.1240; Found: C, 80.97; H, 6.43; N, 5.76. Calc. for $C_{16}H_{15}NO$: C, 80.98; H, 6.37; N, 5.90% [8].

1-Ethyl-1,2,3,4-tetrahydroquinolin-2-one (23d)

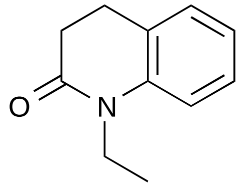

A solution of 3,4-dihydro-2(1*H*)-quinolinone (2.21 g, 15.0 mmol) in anhydrous DMF (25 mL) was cooled to 0 °C under Ar, whereupon NaH (60% dispersion in mineral oil, 0.99 g, 22.5 mmol) was added, and the resultant slurry was stirred for 30 min. Iodoethane (1.61 mL, 20.0 mmol) was added, and the solution was stirred at rt for 40 h. The solution was diluted with H_2O, and extracted with EtOAc. The organics were washed with sat. NH_4Cl, H_2O and

brine, dried ($MgSO_4$) and evaporated to give a crude yellow oil. This was purified by SiO_2 chromatography (hexane/EtOAc 75:25, with 1% Et_3N as eluent) to give compound **23d** as a light yellow oil (2.38 g, 91%); ^{1}H NMR (600 MHz; $CDCl_3$) δ 1.26 (t, *J* = 7.1 Hz, 3H), 2.60-2.67 (m, 2H), 2.87-2.90 (m, 2H), 3.99 (q, *J* = 7.1 Hz, 2H), 6.97-7.04 (m, 2H), 7.16 (dd, *J* = 7.5, 1.4 Hz, 1H), 7.24 (t, *J* = 7.5 Hz, 1H); ^{13}C NMR (151 MHz; $CDCl_3$) δ 13.0, 25.8, 32.1, 37.5, 114.8, 122.8, 126.7, 127.6, 128.2, 139.8, 170.1; IR (neat) ν_{max}/cm^{-1} 2975w, 2936w, 2849w, 1667s, 1602s, 1500m, 1463m, 1376s, 1255s, 756s; MS (ES): *m/z* = 176.1 $[M+H]^+$; HRMS (ES) calcd. for $C_{11}H_{14}NO$ $[M+H]^+$: 176.1075, found 176.1077 [9].

1-Benzyl-1,2,3,4-tetrahydroquinolin-2-one (23e)

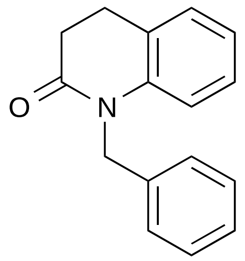

A solution of 3,4-dihydro-2(1*H*)-quinolinone (4.42 g, 30.0 mmol) in anhydrous DMF (50 mL) was cooled to 0 °C under Ar, whereupon NaH (60% dispersion in mineral oil, 1.98 g, 45.0 mmol) was added, and the resultant slurry was stirred for 1 h. Benzyl chloride (4.14 mL, 36.0 mmol) was added, and the solution was stirred at rt for 40 h. The solution was diluted with H_2O, and extracted with EtOAc. The organics were washed with sat. NH_4Cl, H_2O and brine, dried ($MgSO_4$) and evaporated to give a crude colourless oil. This was purified by SiO_2 chromatography (hexane/EtOAc 9:1, with 1% Et_3N as eluent) to give compound **23e** as a white waxy solid (4.83 g, 68%); m.p. 56-57 ^{o}C; ^{1}H NMR (600 MHz; $CDCl_3$) δ 2.77-2.83 (m, 2H), 2.99 (dd, *J* = 8.7, 6.1 Hz, 2H), 5.19 (s, 2H), 6.88(d, *J* = 8.2 Hz, 1H), 6.97 (t, *J* = 7.4Hz, 1H), 7.10 (td, *J* = 7.8, 1.5 Hz, 1H), 7.17 (d, *J* = 7.3 Hz, 1H), 7.20-7.26 (m, 3H), 7.29-7.32 (m, 2H); ^{13}C NMR (151 MHz; $CDCl_3$) δ 25.8, 32.1, 46.4, 115.8, 123.1, 126.5, 126.6, 127.2, 127.6, 128.0, 128.9, 137.2, 140.1, 170.7; IR (neat) ν_{max}/cm^{-1} 3064w, 3027w, 2999w, 1670s, 1600m, 1495s, 1462m, 1373s, 1180, 758s; MS (ES): *m/z* = 238.1 $[M+H]^+$; HRMS (ES) calcd. for $C_{16}H_{16}NO$ $[M+H]^+$: 238.1232, found 238.1228; Found: C, 80.79; H, 6.43; N, 5.87. Calc. for $C_{16}H_{15}NO$: C, 80.98; H, 6.37; N, 5.90% [4].

6-Bromo-4,4-dimethyl-1-(propan-2-yl)-1,2,3,4-tetrahydroquinolin-2-one

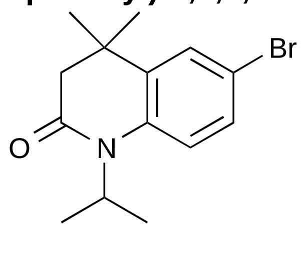

To a solution of 4-bromoaniline (5.0 g, 29.1 mmol) in DCM (100 mL) was added 3,3-dimethylacryloyl chloride (3.40 mL, 30.6 mmol) and the resultant white suspension was stirred for 0.5 h, after which pyridine (9.70 mL, 30.6

mmol) was added and the solution stirred at rt for 16 h. The solution was diluted with DCM and H_2O, washed with sat. NH_4Cl, H_2O, dried ($MgSO_4$) and evaporated to give a crude light brown solid (33 g) which was recrystallised from hexane to give *N*-(4-bromophenyl)-3-methylbut-2-enamide as a white solid (6.87 g, 94%). *N*-(4-Bromophenyl)-3-methylbut-2-enamide (4.5 g, 17.7 mmol) and $AlCl_3$ (3.54 g, 26.6 mmol) were added to anhydrous DCM (90 mL) under Ar and the resultant solution stirred vigorously for 2.5 h at rt. The reaction was then cooled to 0 °C, quenched slowly with H_2O, diluted with DCM, stirred with 20% NaOH (w/v) until the solution turned off-white, then further washed with H_2O, dried ($MgSO_4$) and evaporated to give 6-bromo-4,4-dimethyl-1,2,3,4-tetrahydroquinolin-2-one (4.07 g, 90%). To a solution of 6-bromo-4,4-dimethyl-1,2,3,4-tetrahydroquinolin-2-one (4.07 g, 16.02 mmol) in anhydrous DMF (50 mL) was added crushed KOH (2.70 g, 48.06 mmol) and the resultant slurry stirred for 1 h at 50 °C under Ar. To this was added 2-iodopropane (4.8 mL, 48.06 mmol) and the solution stirred at 50 °C for 40 h. The reaction was quenched with H_2O, diluted with EtOAc, washed with sat. NH_4Cl, H_2O and brine, dried ($MgSO_4$) and evaporated to give a crude clear oil. This was purified by SiO_2 chromatography (hexane/EtOAc 9:1, with 1% Et_3N as eluent) to give 6-bromo-4,4-dimethyl-1-(propan-2-yl)-1,2,3,4-tetrahydroquinolin-2-one as a colourless oil (2.19 g, 46%): ^{1}H NMR (700 MHz; $CDCl_3$) δ 1.27 (s, 6H), 1.51 (d, J = 7.0 Hz, 6H), 2.40 (s, 2H), 4.68 (sept, J = 7.1 Hz, 1H), 7.00 (d, J = 8.7 Hz, 1H), 7.32 (dd, J = 8.7, 2.3 Hz, 1H), 7.36 (d, J = 2.3 Hz, 1H); ^{13}C NMR (176 MHz; $CDCl_3$) δ 20.4, 26.8, 33.2, 47.2, 48.9, 116.4, 118.6, 127.6, 129.9, 138.6, 138.9, 169.8; IR (neat) ν_{max}/cm^{-1} 2964m, 2934w, 2872w, 1671s, 1590m, 1484s, 1417m, 1332s, 1253s, 811s; MS (ES): *m/z* = 296.4 [M+H]$^+$; HRMS (ES) calcd. for $C_{14}H_{19}NOBr$ [M+H]$^+$: 296.0650, found: 296.0658.

6-Bromo-4,4-dimethyl-1-(propan-2-yl)-1,2,3,4-tetrahydroquinoline (26)

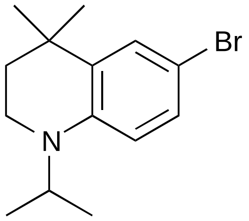

To a solution of 6-bromo-4,4-dimethyl-1-(propan-2-yl)-1,2,3,4-tetrahydroquinolin-2-one (1.17 g, 3.96 mmol) in anhydrous toluene (20 mL) under Ar was added borane dimethyl sulfide complex (2.0 M in THF, 2.0 mL, 4.00 mmol) dropwise and the resultant solution stirred under reflux for 16 h. The solution was cooled to rt, 10% aq Na_2CO_3 (25 ml) added and the solution stirred for 0.5 h. The solution was then diluted with EtOAc, washed with brine, dried ($MgSO_4$) and evaporated to give a crude colourless oil. This was purified by SiO_2 chromatography (hexane/EtOAc 9:1, with 1% Et_3N as eluent) to give compound **26** as a colourless oil (1.00 g, 89%): ^{1}H NMR (700 MHz; $CDCl_3$) δ 1.19 (d, J = 6.6 Hz, 6H), 1.25 (s, 6H), 1.67 (t, J = 6.1 Hz, 2H), 3.14 (t, J = 6.1 Hz, 2H), 4.05 (sept, J = 6.6 Hz, 1H), 6.55 (d, J = 8.7 Hz, 1H), 7.11 (dd, J = 8.9, 2.2 Hz, 1H), 7.23 (d, J = 2.3 Hz, 1H); ^{13}C NMR (101 MHz, $CDCl_3$) δ 18.9, 30.3, 32.5, 36.6, 36.9, 47.4, 107.2, 112.7, 128.4, 128.7, 129.6, 134.2, 143.4; IR (neat) ν_{max}/cm^{-1} 2966m, 2929w, 2866, 1587m, 1490, 1448, 793s; MS(ES):

m/z = 282.4 $[M+H]^+$; HRMS (ES) calcd. for $C_{14}H_{21}NBr$ $[M+H]^+$: 282.0857, found 282.0876.

Methyl 4-(tetramethyl-1,3,2-dioxaborolan-2-yl)benzoate (27)

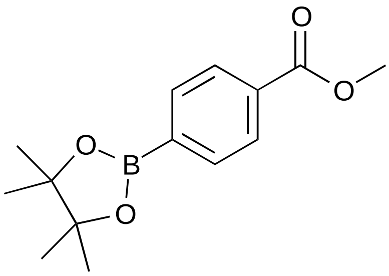

Methyl 4-iodobenzoate (1.57 g, 6.00 mmol), B_2Pin_2 (1.68 g, 6.60 mmol), KOAc (1.77 g, 18.0 mmol) and $Pd(dppf)Cl_2.CH_2Cl_2$ (0.147 g, 0.18 mmol) were added to anhydrous DMSO (12 mL, degassed via freeze-pump-thaw) under Ar. The resultant suspension was then stirred at 80 °C overnight. The mixture was cooled, diluted with H_2O and extracted with EtOAc (3×). The organics were washed with H_2O and brine, dried ($MgSO_4$) and evaporated to give a crude solid (2.0 g). The crude solid was distilled under vacuum using a Kugelrohr (130–140 °C, 0.64 Torr) to give a white solid which was then recrystallised from MeOH at −20 °C to give compound **27** as a colourless crystalline solid (1.27 g, 81%); m.p. 77–78 °C; ^1H NMR (700 MHz; $CDCl_3$) δ 1.35 (s, 12H) 3.91 (s, 3H), 7.85-7.89 (m, 2H), 8.00-8.04 (m, 2H); ^{13}C NMR (176 MHz; $CDCl_3$) δ 25.1, 52.3, 84.4, 128.8, 132.5, 134.9, 167.3; ^{11}B NMR (128 MHz, $CDCl_3$) δ 30.63; IR (neat) ν_{max}/cm^{-1} 2980w, 2955w, 2935w, 1719s, 1507m, 1398s, 1359s, 1267s, 707s; MS(EI): m/z = 262.1 $[M]^+$; Found: C, 64.32; H, 7.27. Calc. for $C_{14}H_{19}BO_4$: C, 64.15; H, 7.31% [10].

Note: The ^{13}C resonance for the carbon atom bonded to boron was not observed, despite processing with larger line broadening.

Methyl 4-[4,4-dimethyl-1-(propan-2-yl)-1,2,3,4-tetrahydroquinolin-6-yl]benzoate (28)

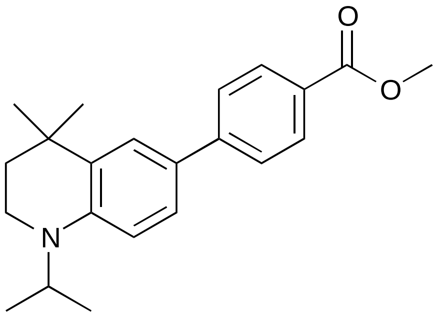

Compound **21** (0.0925 g, 0.281 mmol) was dissolved in DMSO/H2O (6 mL, 5:1), and the resultant solution was degassed via freeze-pump-thaw. Under Ar, compound **27** (0.081 g, 0.309 mmol), K_3PO_4 (0.12 g, 0.562 mmol) and $Pd(dppf)Cl_2$ (6 mg, 0.00843 mmol) were added, and the resultant suspension was stirred at 80 °C for 16 h. The solution was cooled, diluted with H_2O, and extracted with EtOAc (3×). The organics were washed with H_2O and brine,

dried ($MgSO_4$) and evaporated to give a crude solid. This was purified by SiO_2 chromatography (hexane/EtOAc 92:8, with 1% Et_3N as eluent) to give compound **28** as a light yellow crystalline solid (0.065 g, 68%): m.p. 103-104 ^oC; ^1H NMR (700 MHz; $CDCl_3$) δ 1.24 (d, *J* = 6.6 Hz, 6H), 1.34 (s, 6H), 1.69–1.79 (m, 2H), 3.18–3.29 (m, 2H), 3.92 (s, 3H), 4.19 (sept, *J* = 6.7 Hz, 1H), 6.77 (d, *J* = 8.7 Hz, 1H), 7.38 (dd, *J* = 8.6, 2.3 Hz, 1H), 7.50 (d, *J* = 2.4 Hz, 1H), 7.56–7.69 (m, 2H), 8.00–8.13 (m, 2H); ^{13}C NMR (176 MHz; $CDCl_3$) δ 19.1, 30.4, 32.4, 36.7, 37.1, 47.4, 52.2, 111.2, 124.8, 125.8, 126.0, 126.2, 127.2, 130.2, 132.2, 144.7, 146.3, 167.5; IR (neat) ν_{max}/cm^{-1} 2978w, 2953w, 2909w, 2847w, 1708s, 1596m, 1492m, 1434m, 1260s, 775s, MS(ES): *m/z* = 338.7 $[M+H]^+$; HRMS (ES) calcd. for $C_{22}H_{28}NO_2$ $[M+H]^+$: 338.2120, found 338.2121.

4,4-Dimethyl-1-(propan-2-yl)-6-(tetramethyl-1,3,2-dioxaborolan-2-yl)-1,2,3,4-tetrahydroquinoline (29)

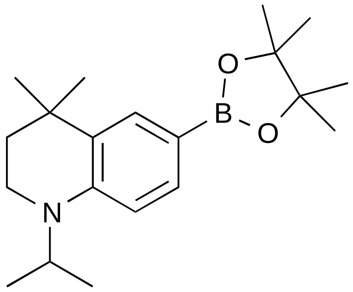

Compound **21** (0.89 g, 2.70 mmol) was dissolved in anhydrous DMSO (12 mL) in a Schlenk flask, and the resultant solution was degassed using the freeze-pump-thaw method, and the flask refilled with Ar. Pd(dppf)Cl_2 (0.099 g, 0.135 mmol), KOAc (0.53 g, 5.39 mmol) and B_2Pin_2 (0.719 g, 2.83 mmol) were then added under Ar. The resultant mixture was then stirred at 80 °C for 18 h. The mixture was cooled, diluted with EtOAc, washed with H_2O and brine, dried ($MgSO_4$) and evaporated to give a crude solid. This was passed through a short SiO_2 plug (eluting with DCM), evaporated, and then recrystallised from MeOH at −20 °C to give compound **29** as a colourless crystalline solid (0.52 g, 59%): m.p. = 146-147 ^oC; ^1H NMR (600 MHz, $CDCl_3$) δ 1.20 (d, *J* = 6.6 Hz, 6H), 1.30 (s, 6H), 1.32 (s, 12H), 1.66-1.69 (m, 2H), 3.18-3.22 (m, 2H), 4.19 (sept, *J* = 6.6 Hz, 1H), 6.68 (d, *J* = 8.4 Hz, 1H), 7.53 (dd, *J* = 8.4, 1.4 Hz, 1H), 7.63 (d, *J* = 1.2 Hz, 1H); ^{13}C NMR (151 MHz, $CDCl_3$) δ 19.1, 25.0, 30.2, 32.2, 36.8, 37.1, 47.2, 83.2, 110.0, 130.7, 132.5, 134.5, 146.9; ^{11}B NMR (128 MHz, $CDCl_3$) δ 30.81; IR (neat) ν_{max}/cm^{-1} 2967w, 2932w, 2867w, 1601s, 1511w, 1466m, 1405m, 1312s, 1140s, 963m, 804s; MS (ES): *m/z* = 329.2 $[M+H]^+$; HRMS (ES) calcd. for $C_{20}H_{33}NBO_2$ $[M+H]^+$: 329.2641, found: 329.2635; Found: C, 73.51; H, 9.82; N 4.29. Calc. for $C_{20}H_{32}NBO_2$: C, 72.95; H, 9.80; N 4.25%.

Note: The ^{13}C resonance for the carbon atom bonded to boron was not observed, despite processing with larger line broadening.

4,4-Dimethyl-1-(propan-2-yl)-6-(tetramethyl-1,3,2-dioxaborolan-2-yl)-1,4-dihydroquinoline (30)

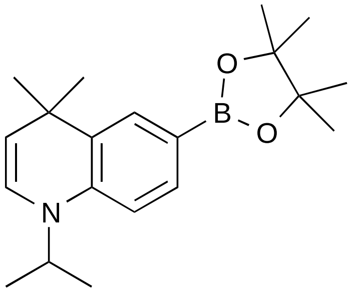

Compound **22** (0.53 g, 1.62 mmol) was dissolved in anhydrous DMSO (10 mL) in a Schlenk flask, and the resultant solution was degassed using the freeze-pump-thaw method, and the flask refilled with Ar. Pd(dppf)Cl_2 (0.059 g, 0.0812 mmol), KOAc (0.319 g, 3.25 mmol) and B_2Pin_2 (0.455 g, 1.79 mmol) were then added under Ar. The resultant mixture was then stirred at 80 °C for 18 h. The mixture was cooled, diluted with EtOAc, washed with H_2O and brine, dried ($MgSO_4$) and evaporated to give a crude residue. This was passed through a short SiO_2 plug (eluting with DCM), evaporated, and then recrystallised from MeOH at −20 °C to give **30** as a colourless crystalline solid (0.27 g, 53%); m.p. = 119-120 ^oC; ^1H NMR (700 MHz; $CDCl_3$) δ 1.28 (d, *J* = 6.6 Hz, 6H) 1.34 (s, 18H), 4.24 (sept, *J* = 6.7 Hz, 1H), 4.58(d, *J* = 7.9 Hz, 1H), 6.16 (d, *J* = 7.9 Hz, 1H), 6.80 (d, *J* = 8.6 Hz, 1H), 7.59 (dd, *J* = 8.3, 1.5 Hz, 1H), 7.70 (d, *J* = 1.5 Hz, 1H); ^{13}C NMR (176 MHz; $CDCl_3$) δ 20.9, 25.1, 33.0, 33.3, 46.8, 83.2, 83.4, 110.3, 110.8, 122.5, 130.9, 133.3, 133.8, 142.4; ^{11}B NMR (128 MHz, $CDCl_3$) δ 30.74; IR (neat) ν_{max}/cm^{-1} 2994w, 2974w, 1663w, 1601m, 1496w, 1397m, 1206m, 809m; MS (ES): *m/z* = 327.2 $[M+H]^+$; HRMS (ES) calcd. for $C_{20}H_{31}NBO_2$ $[M+H]^+$: 327.2484, found: 327.2480; Found: C, 73.47; H, 9.23; N 4.11. Calc. for $C_{20}H_{30}NBO_2$: C, 73.40; H, 9.24; N 4.28%.

4. ^{1}H, ^{13}C and ^{11}B NMR spectra

2-Methyl-*N*-(propan-2-yl)aniline (6)

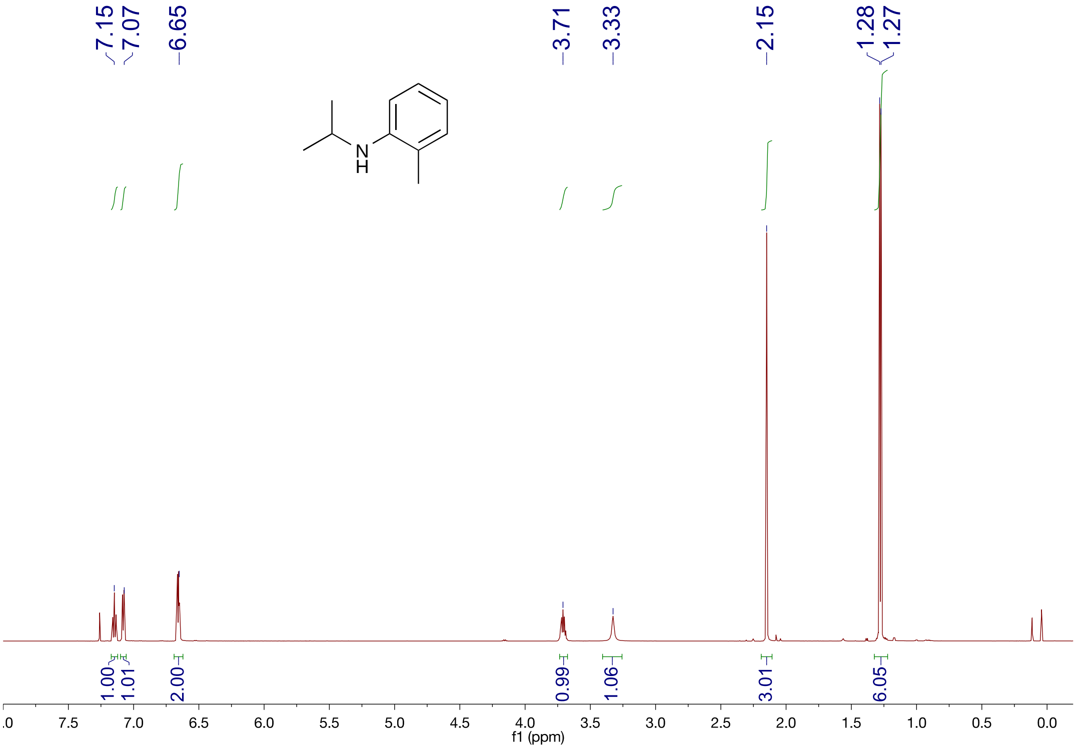

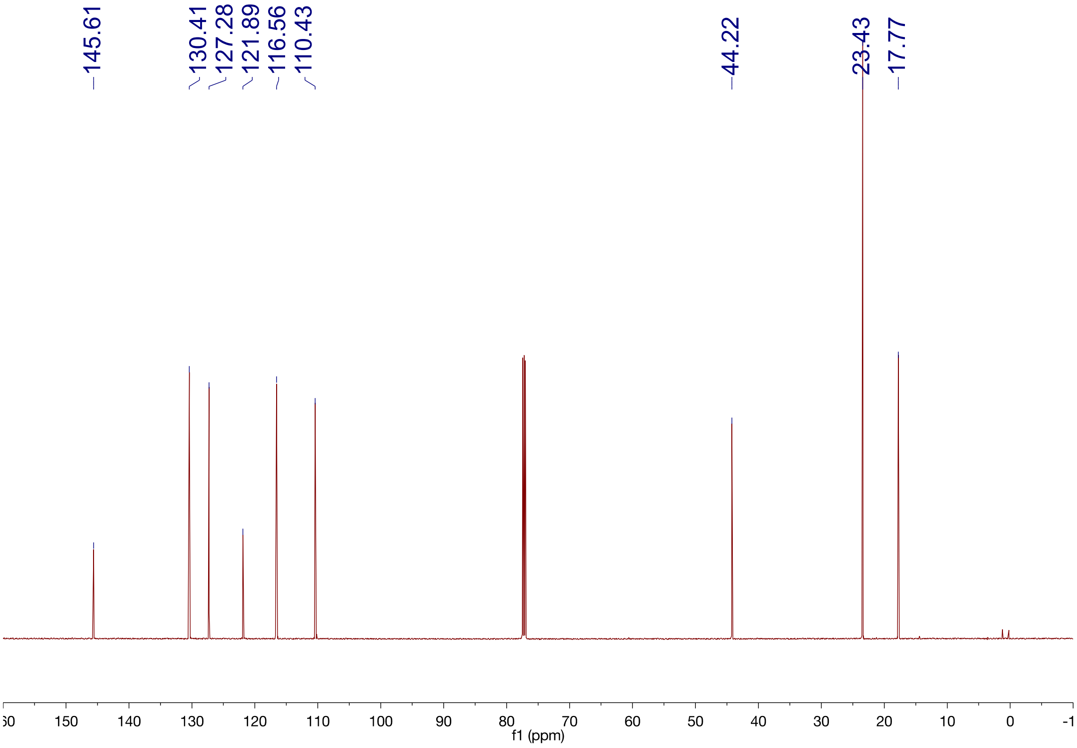

2-Methyl-*N*-(3-methylbut-2-en-1-yl)-*N*-(propan-2-yl)aniline (4)

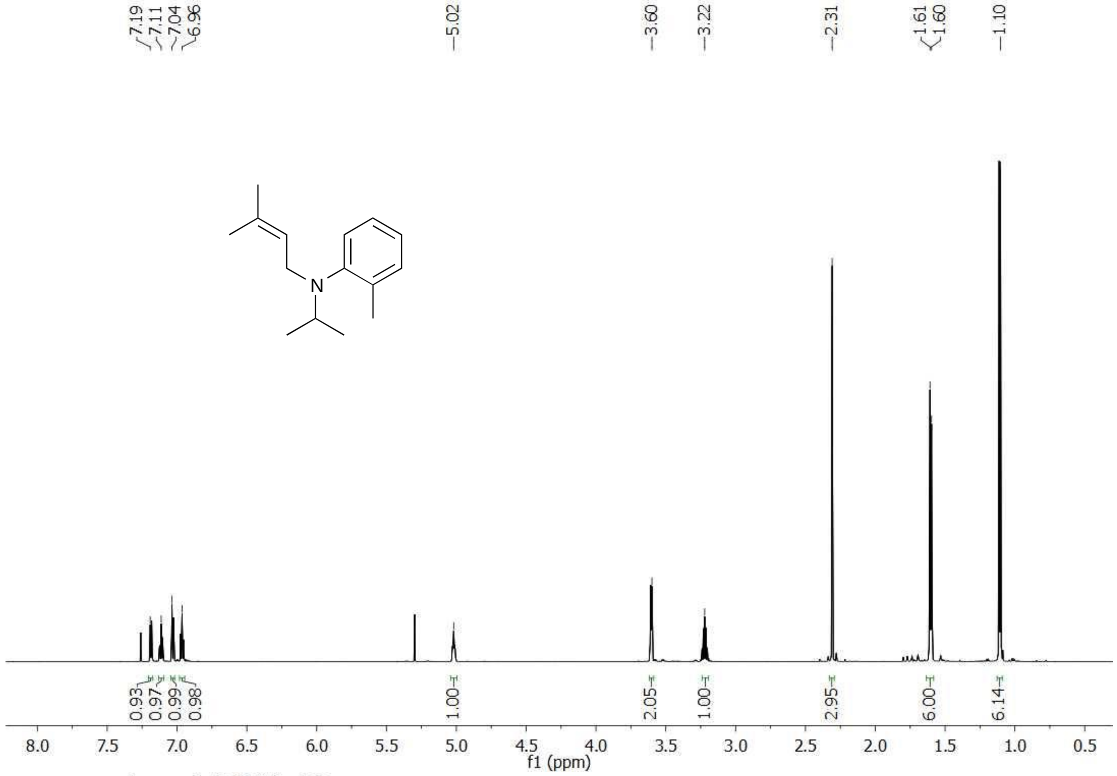

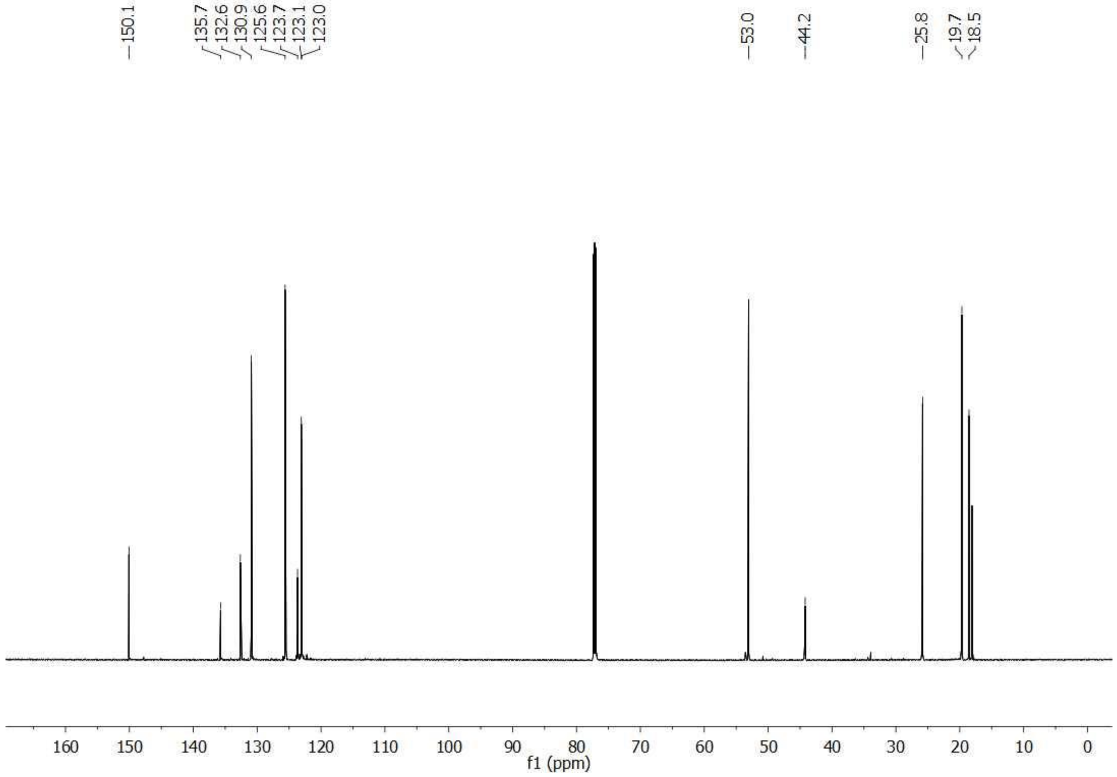

1-Isopropyl-4,4,8-trimethyl-1,2,3,4-tetrahydroquinoline (3)

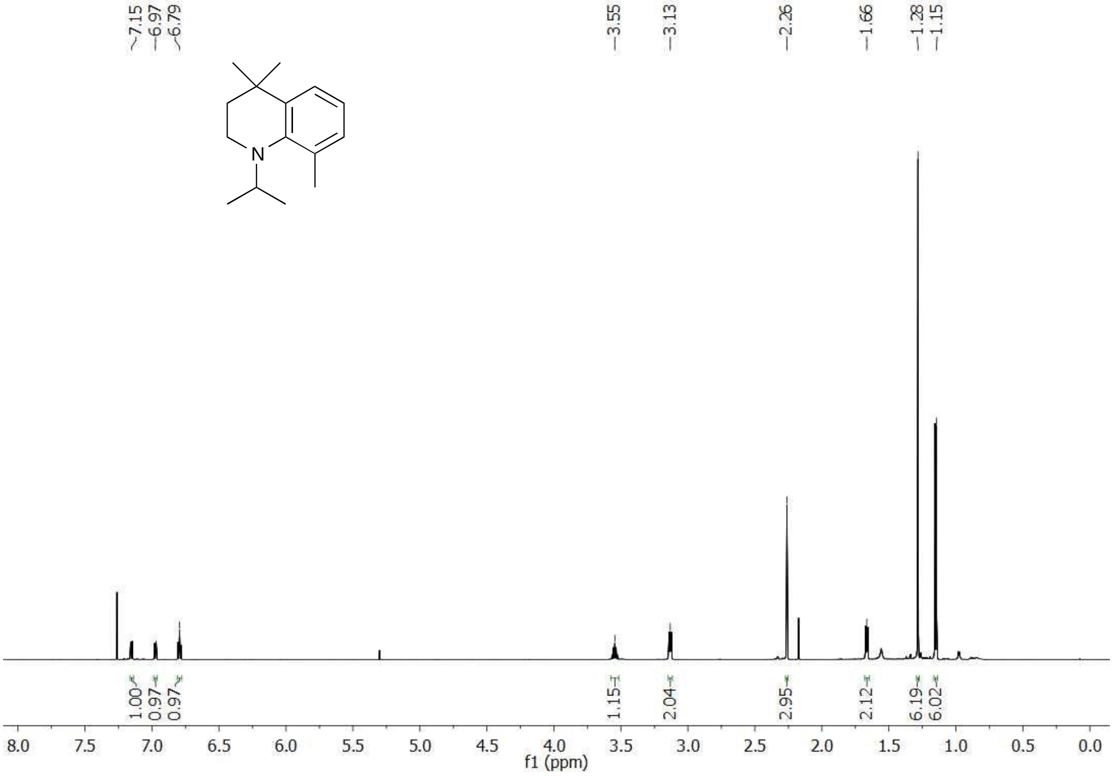

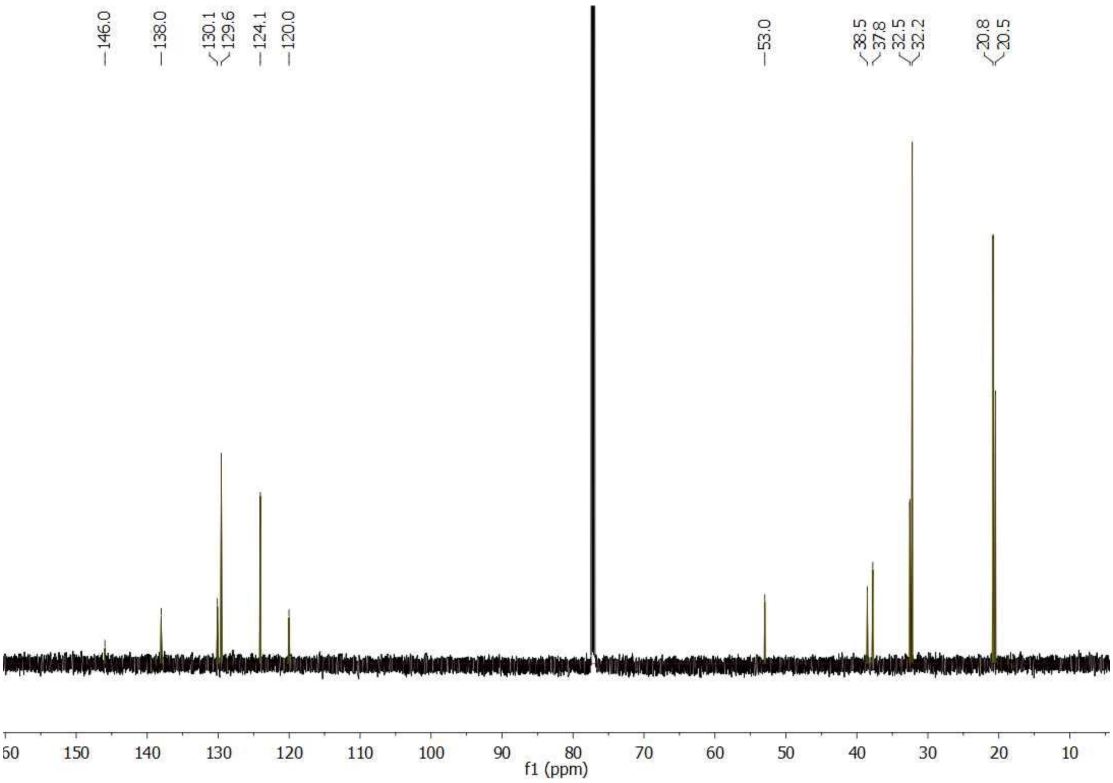

6-Bromo-4,4,8-trimethyl-1-(propan-2-yl)-1,2,3,4-tetrahydroquinoline (7)

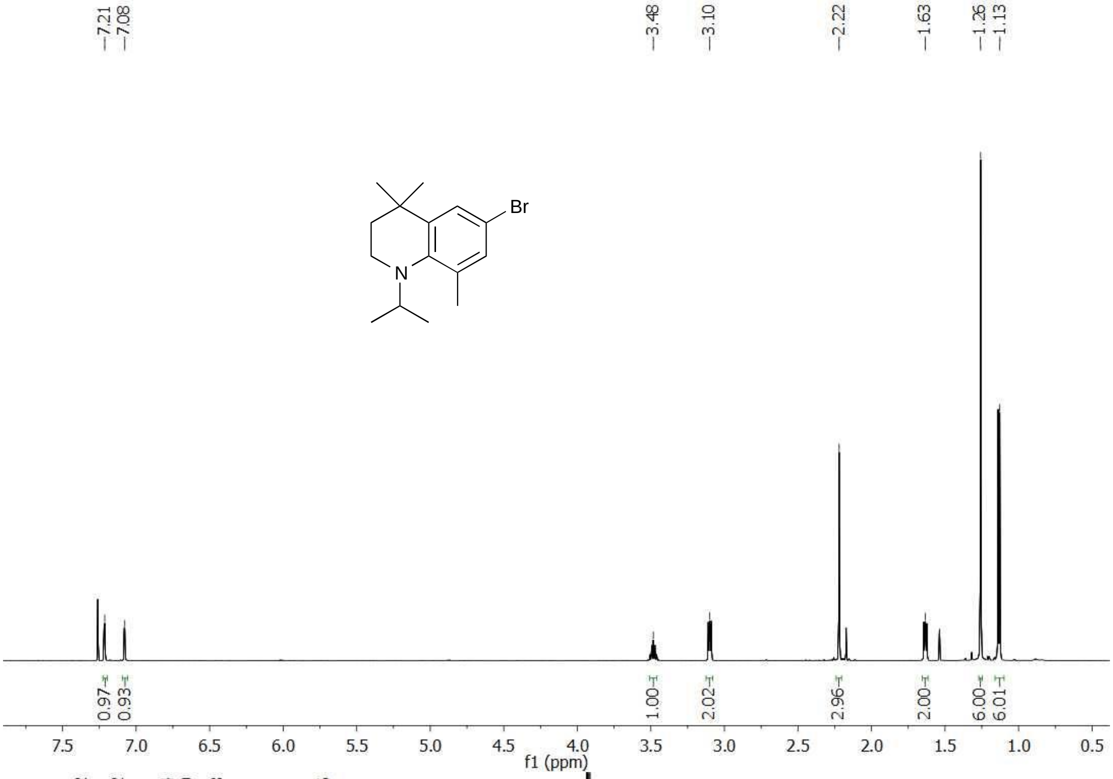

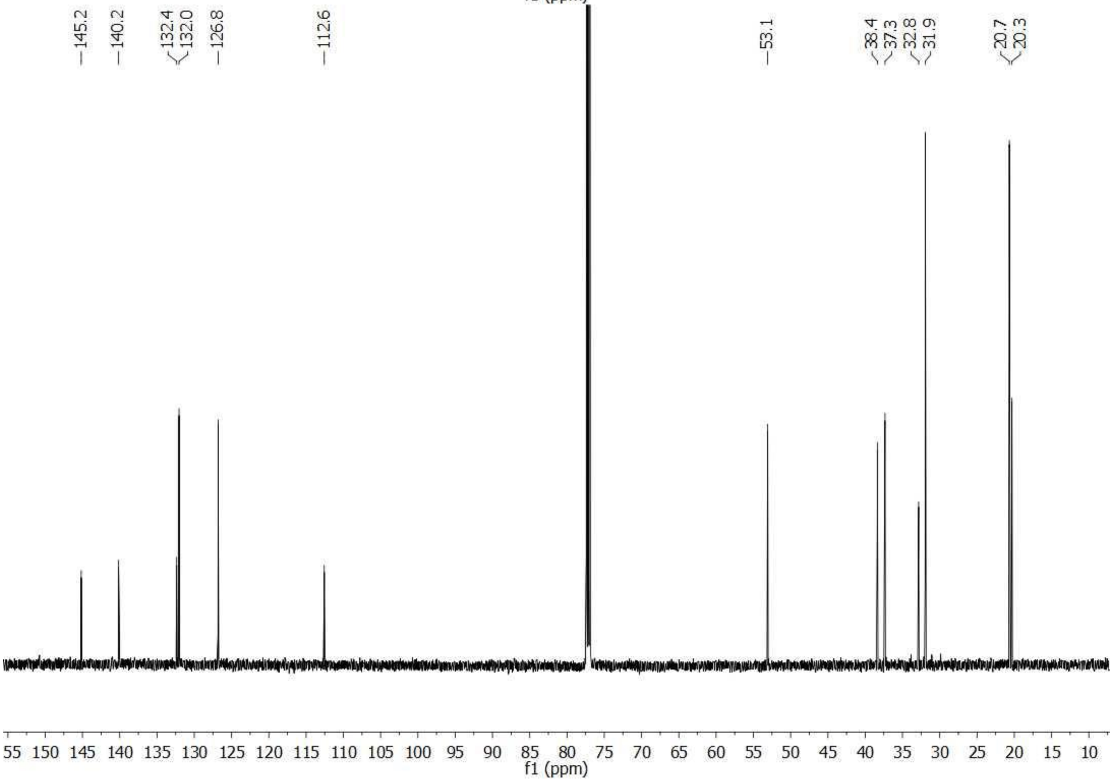

4-[(2-Methylphenyl)amino]butan-2-one (8)

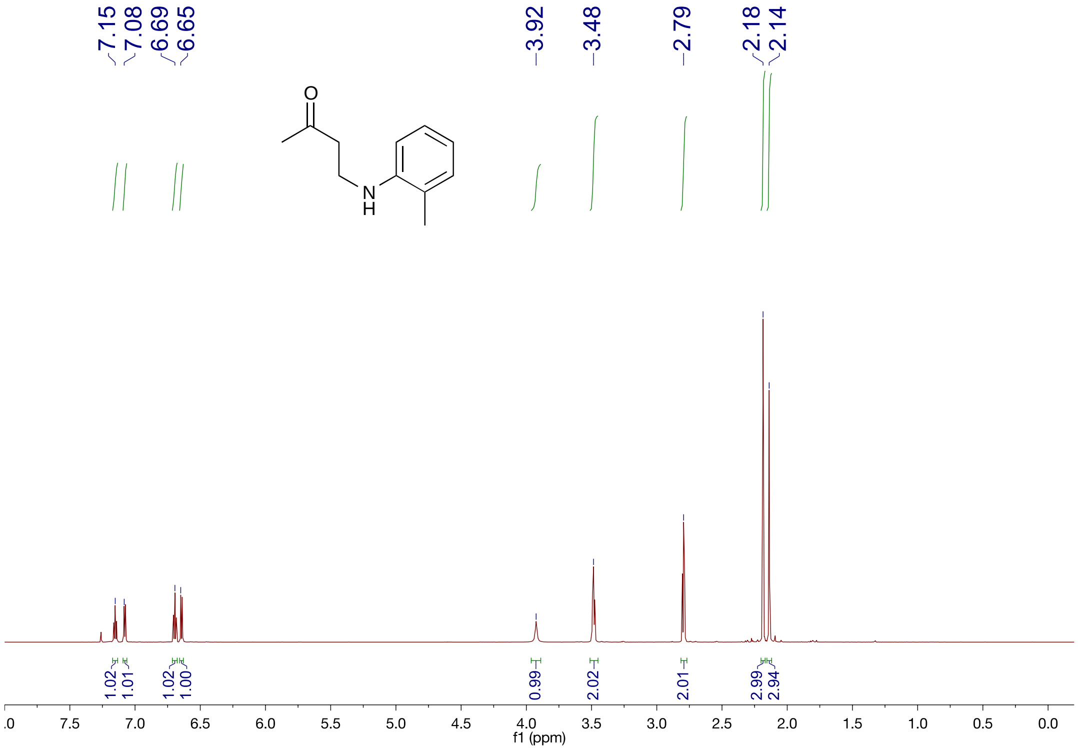

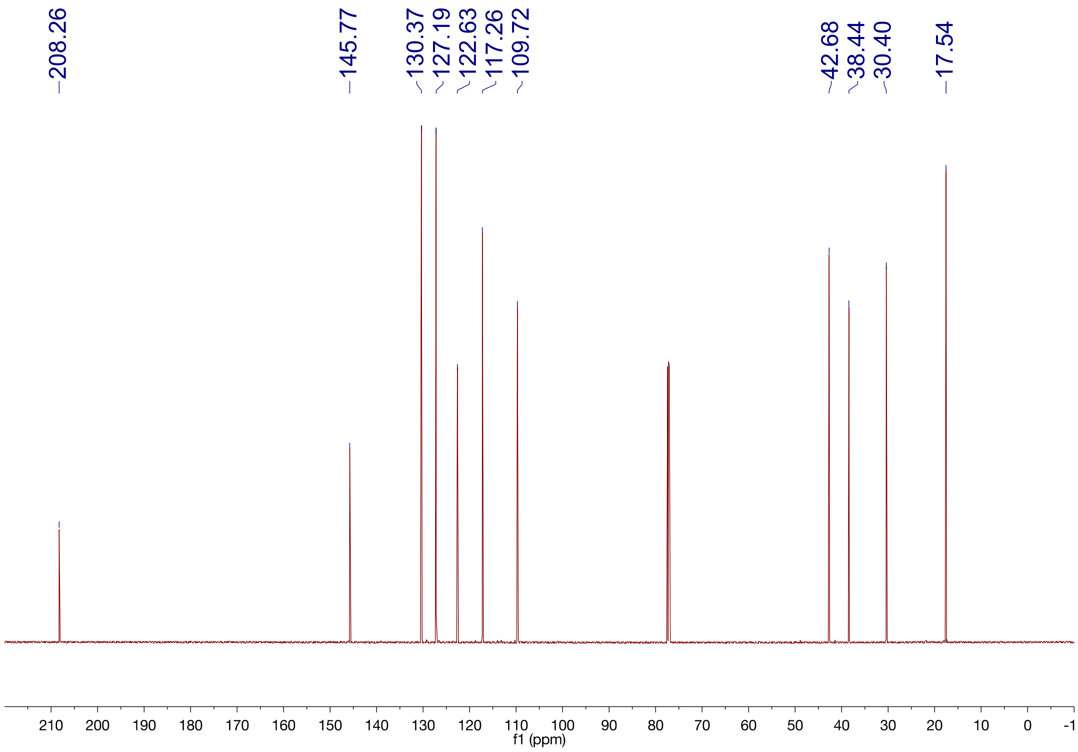

4,4,8-trimethyl-1,2,3,4-tetrahydroquinoline (10)

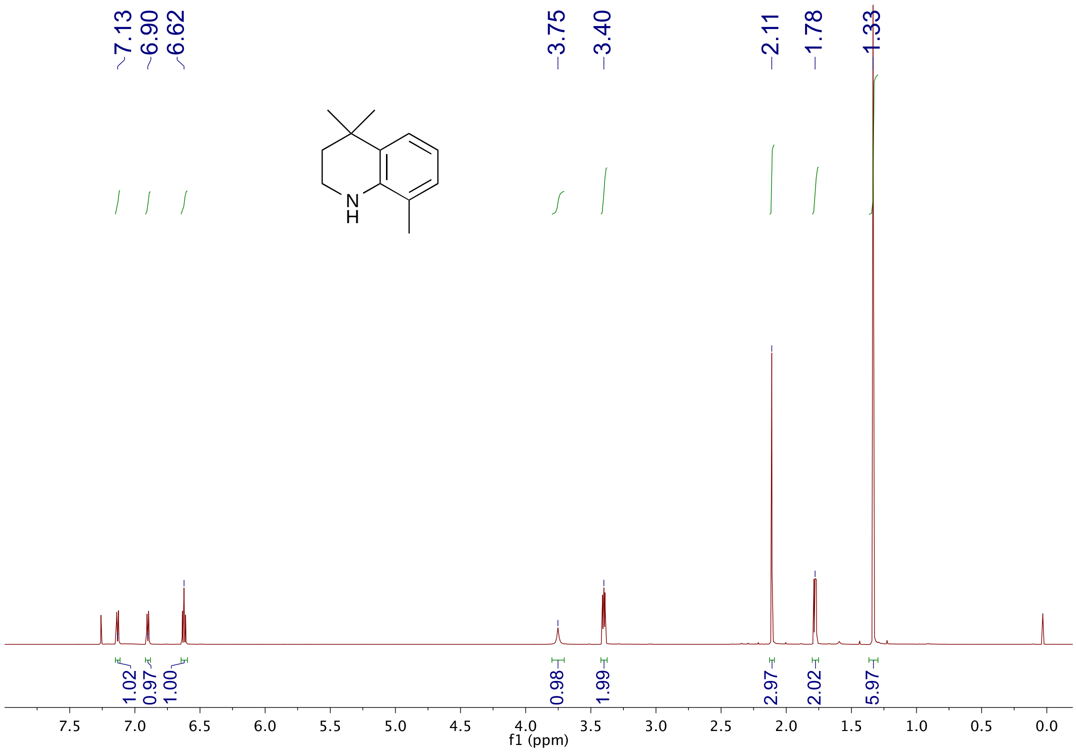

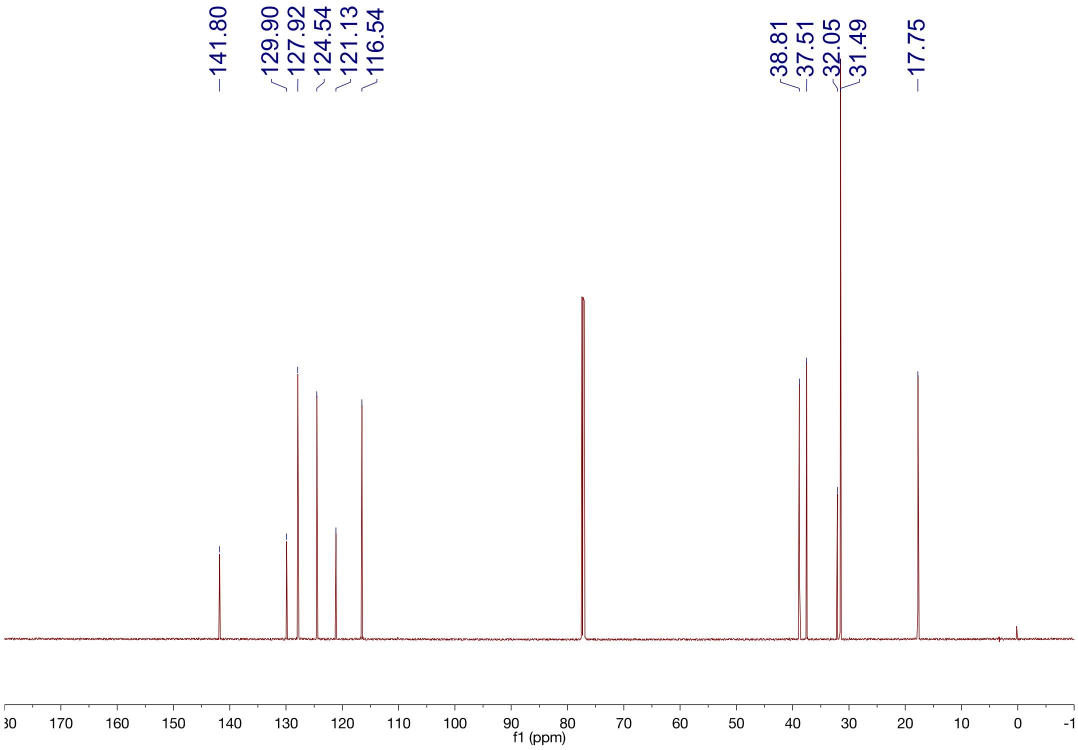

N-(4-Iodo-2-methylphenyl)-3-methylbut-2-enamide (12)

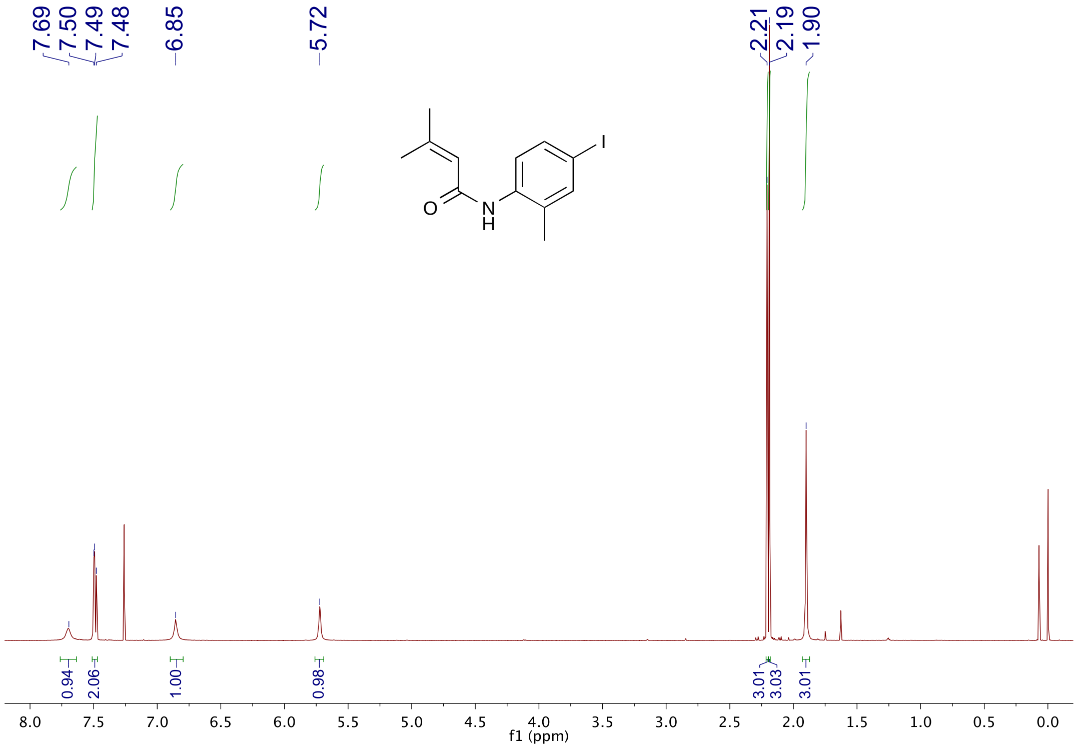

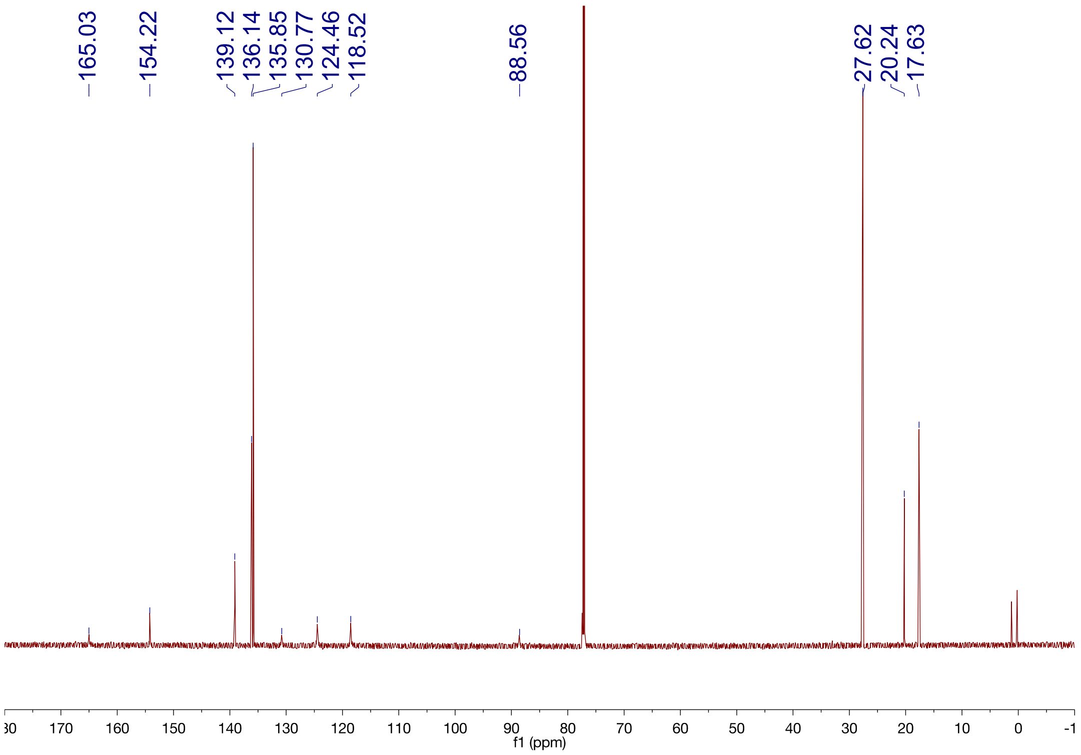

6-Iodo-4,4,8-trimethyl-1,2,3,4-tetrahydroquinolin-2-one (13)

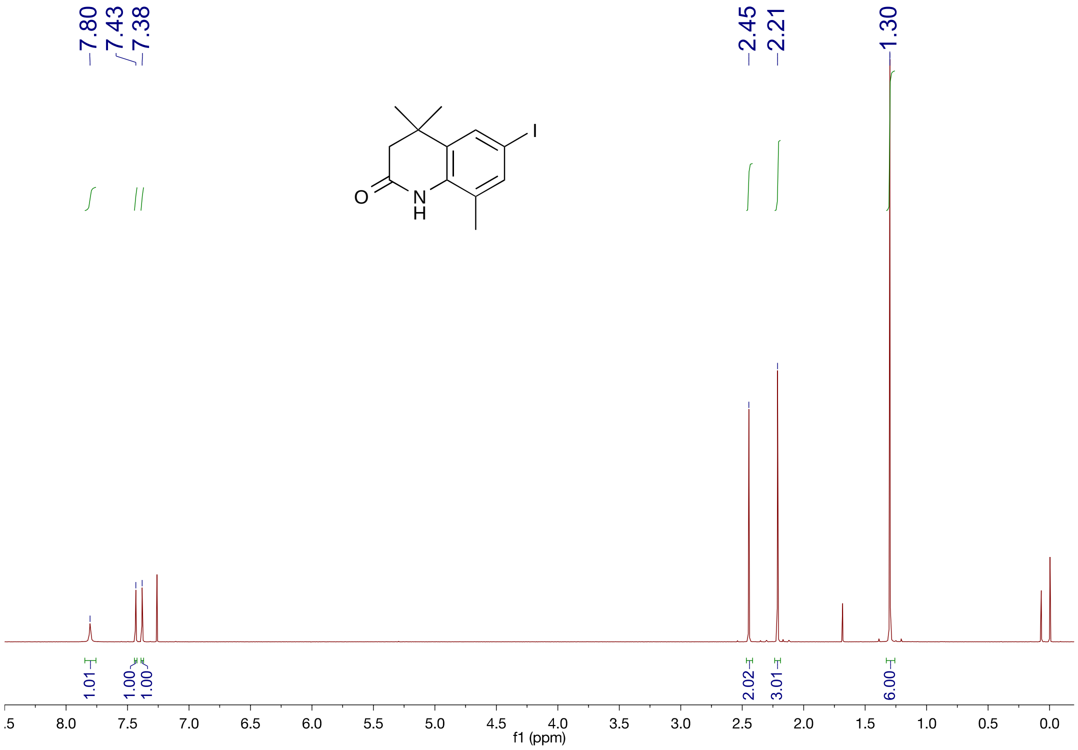

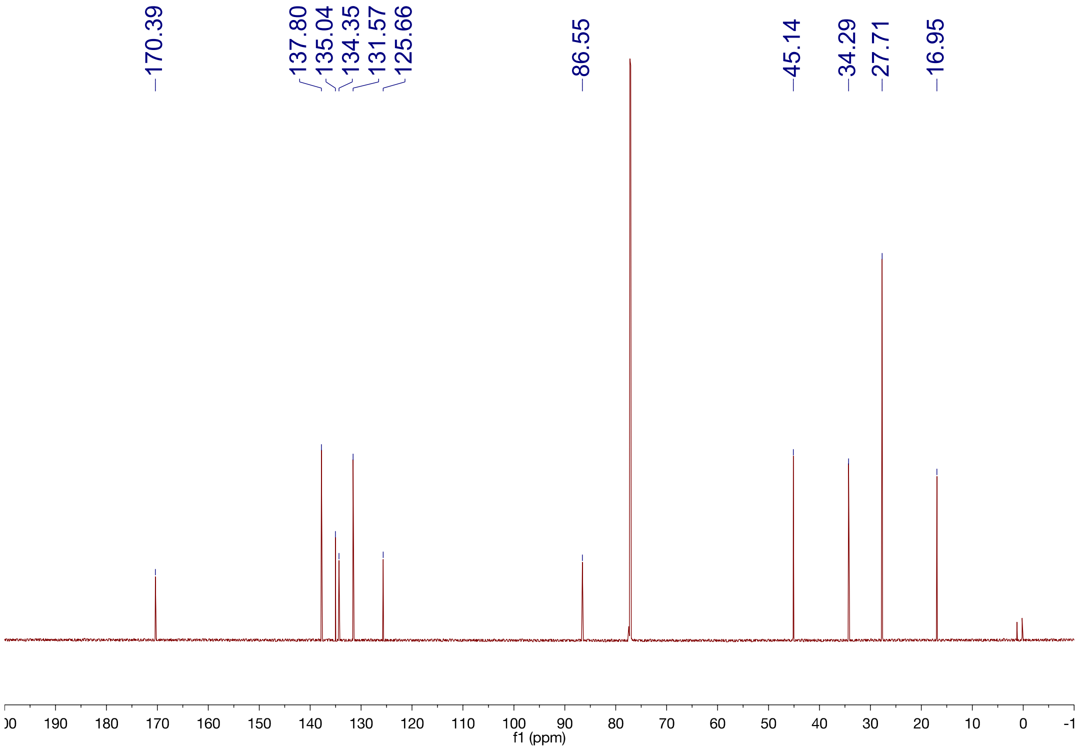

6-Iodo-4,4,8-trimethyl-1,2,3,4-tetrahydroquinoline (14)

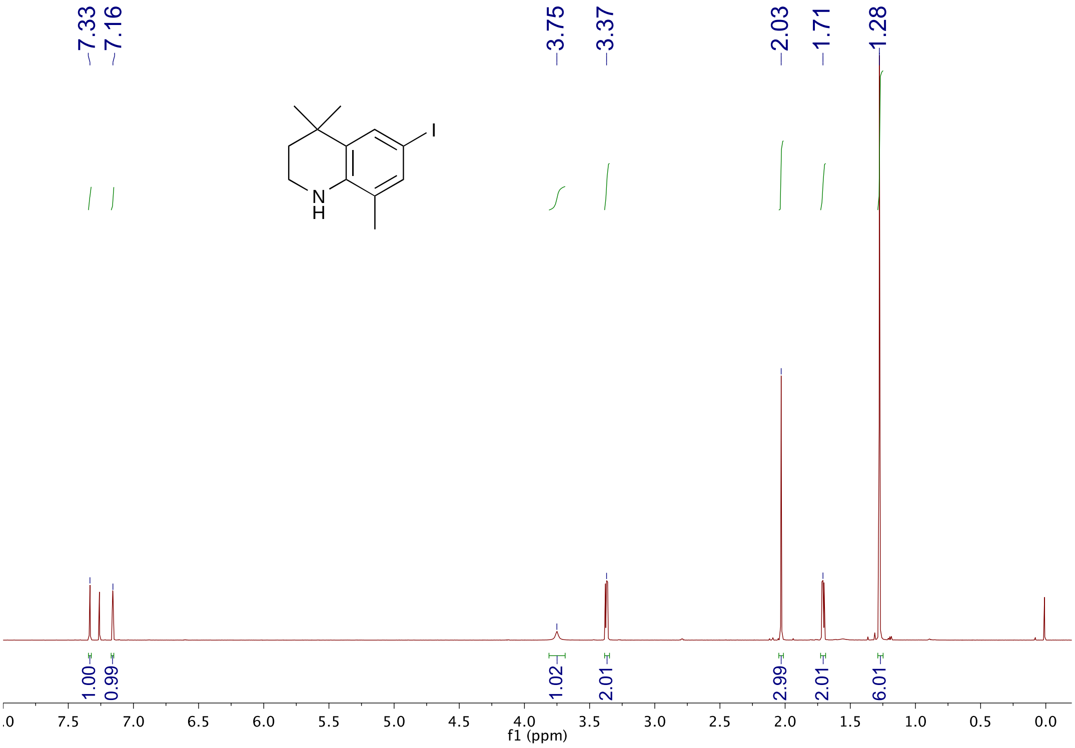

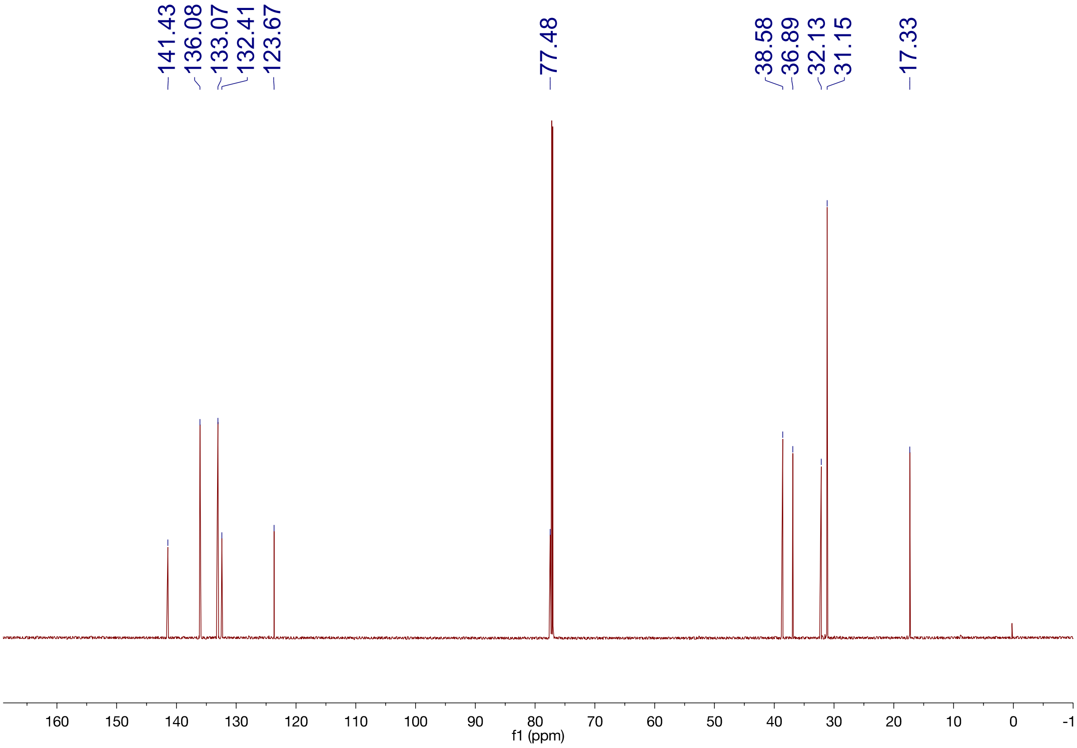

6-Iodo-4,4,8-trimethyl-2-(propan-2-yloxy)-3,4-dihydroquinoline (15b)

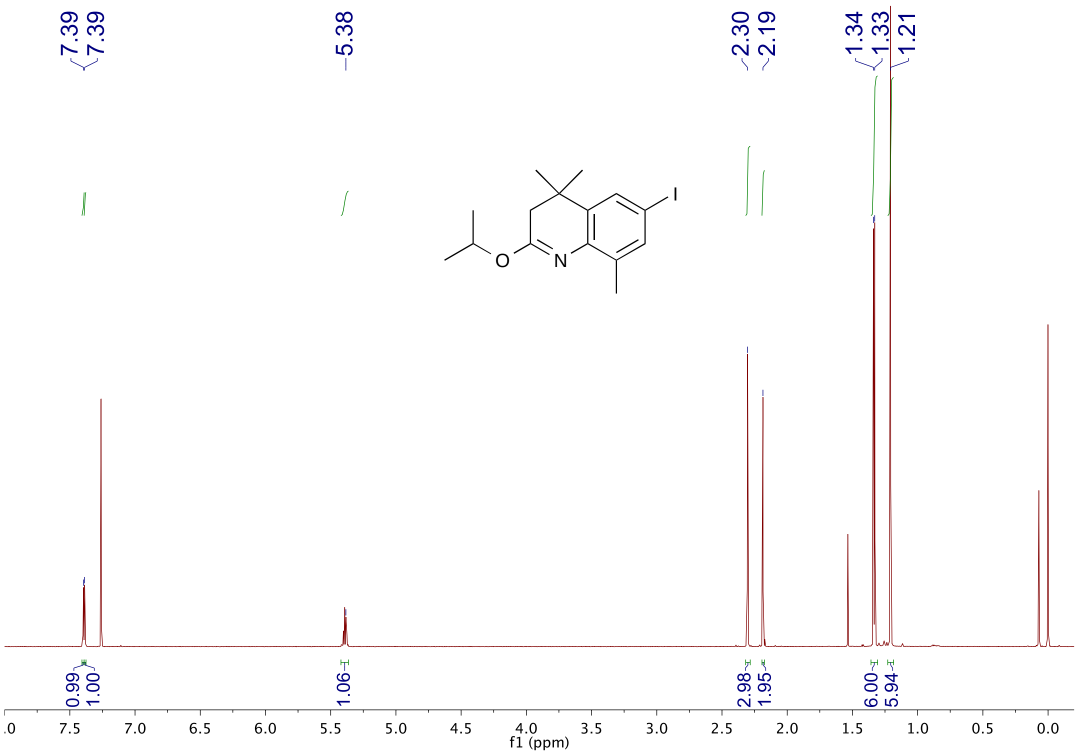

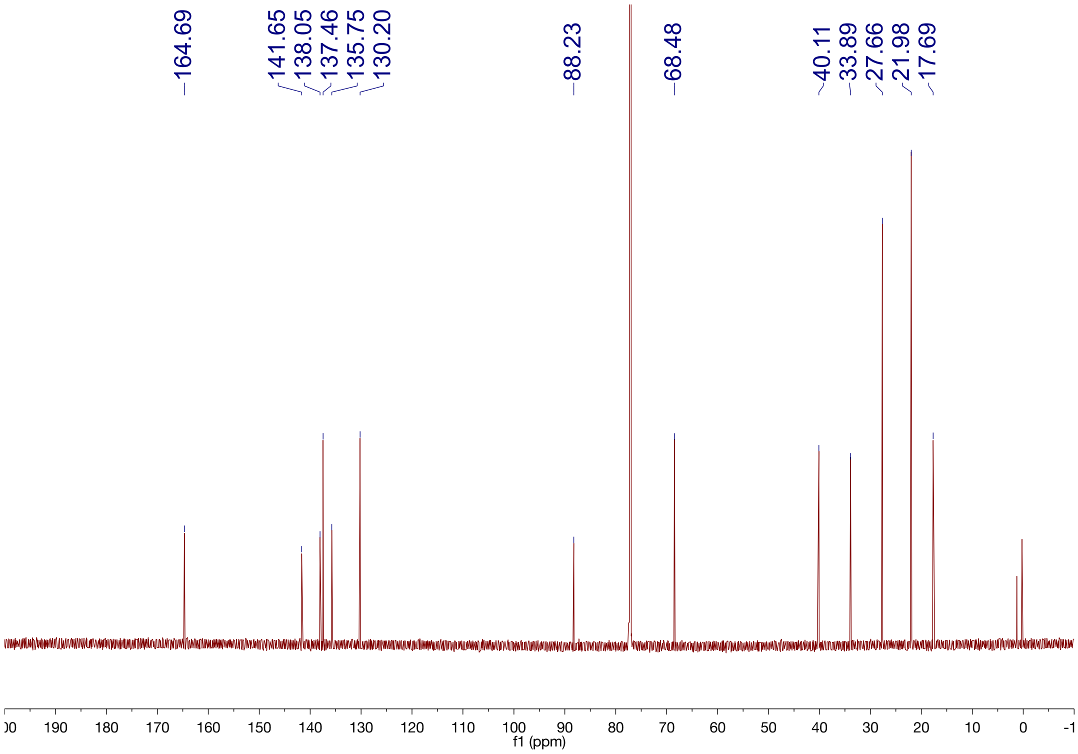

6-Iodo-1,4,4,8-tetramethyl-1,2,3,4-tetrahydroquinoline (16)

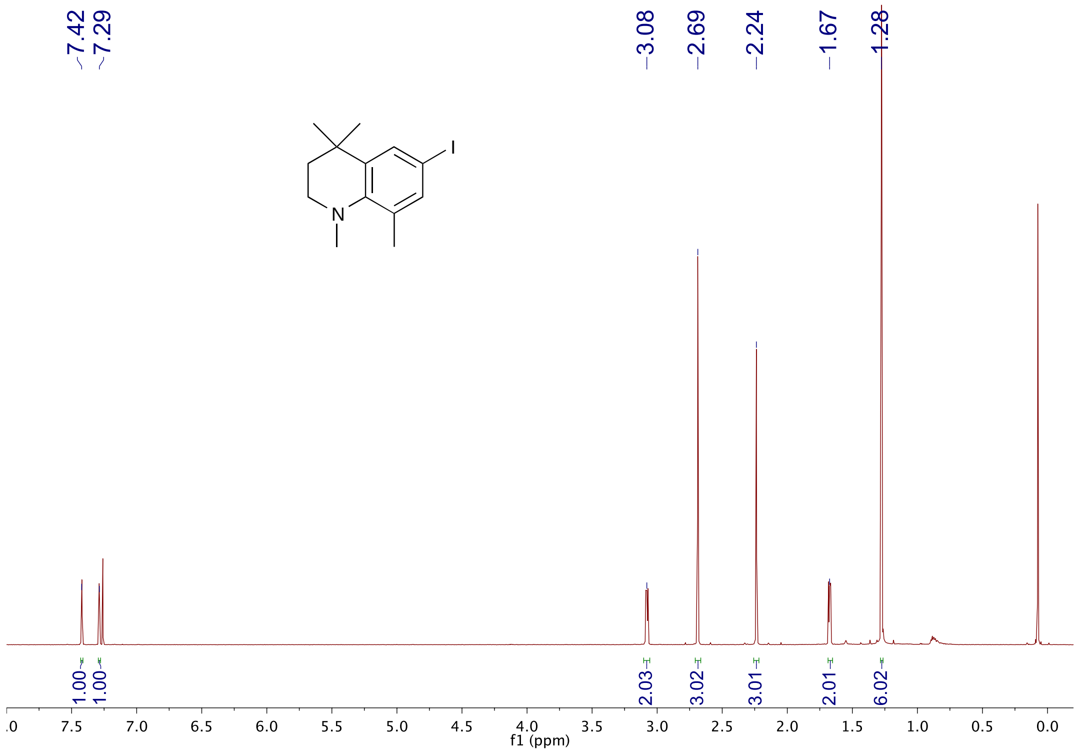

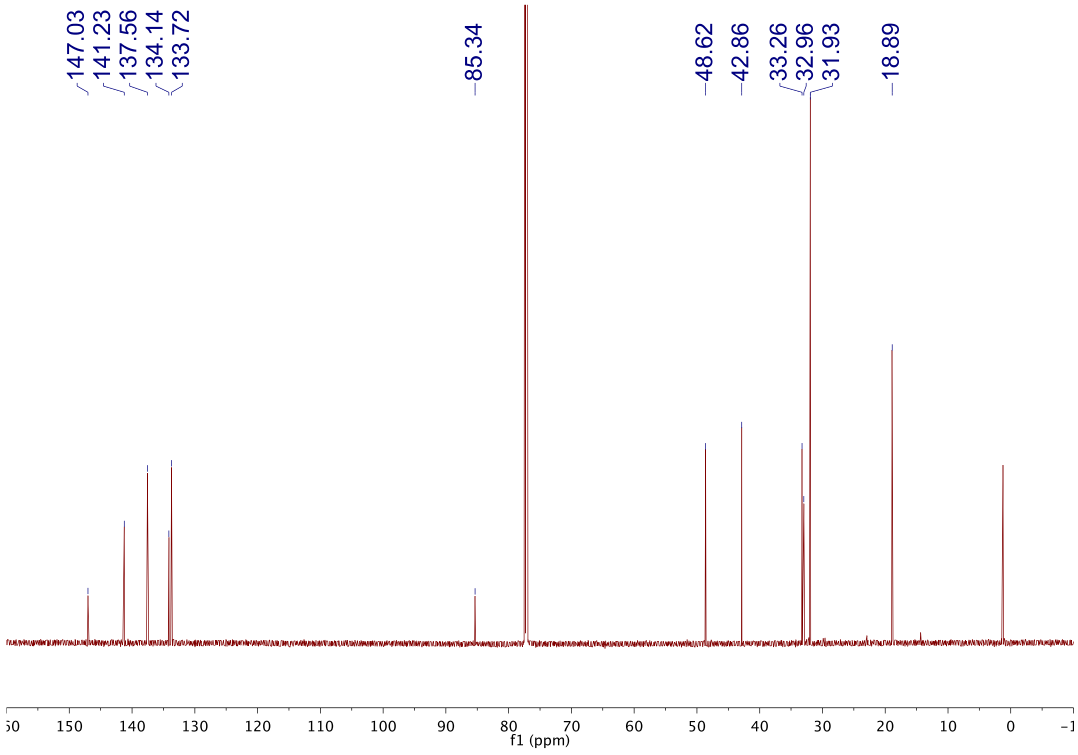

N-(4-Iodophenyl)-3-methylbut-2-enamide (18)

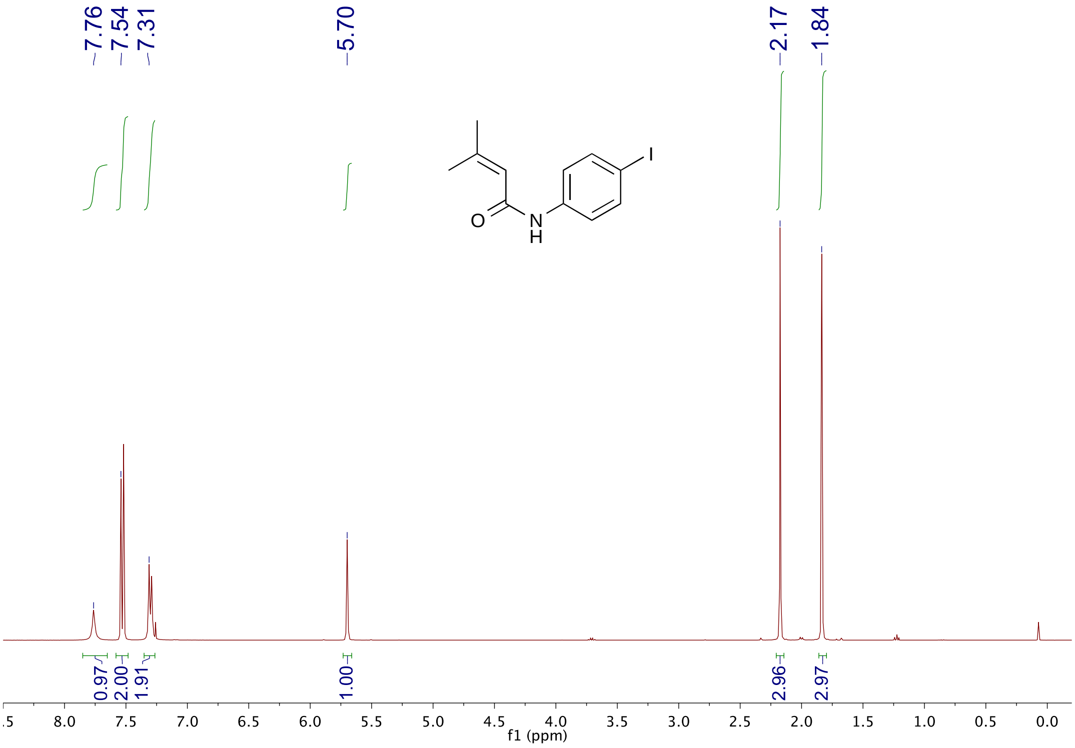

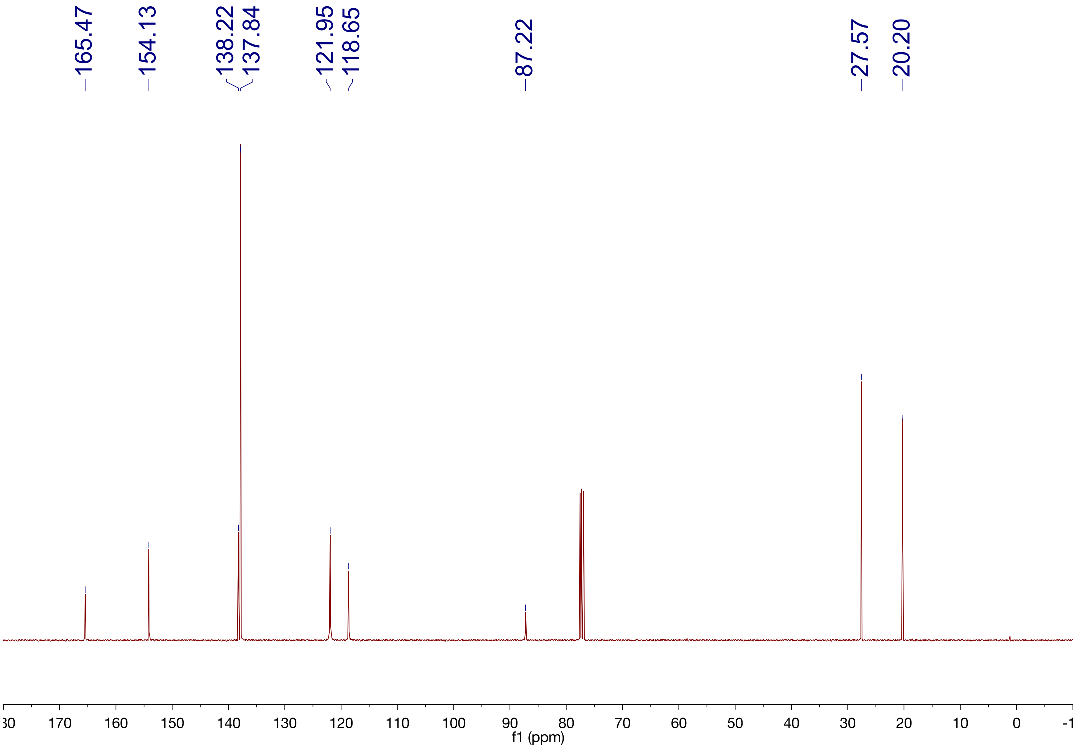

6-Iodo-4,4-dimethyl-1,2,3,4-tetrahydroquinolin-2-one (19)

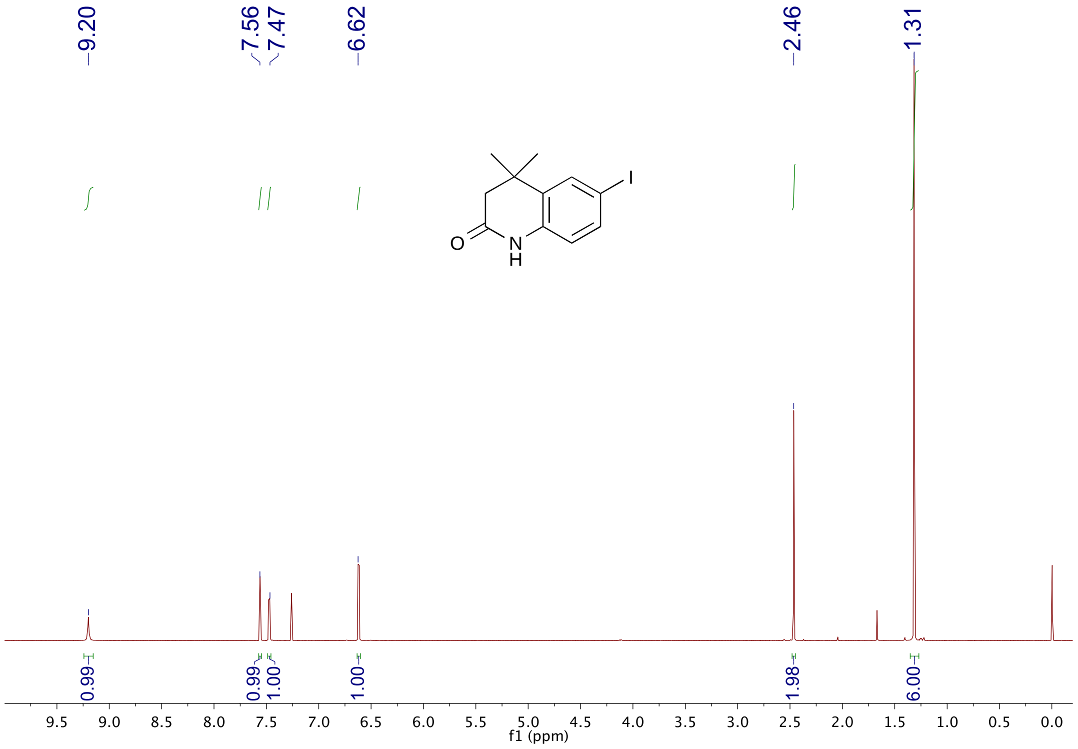

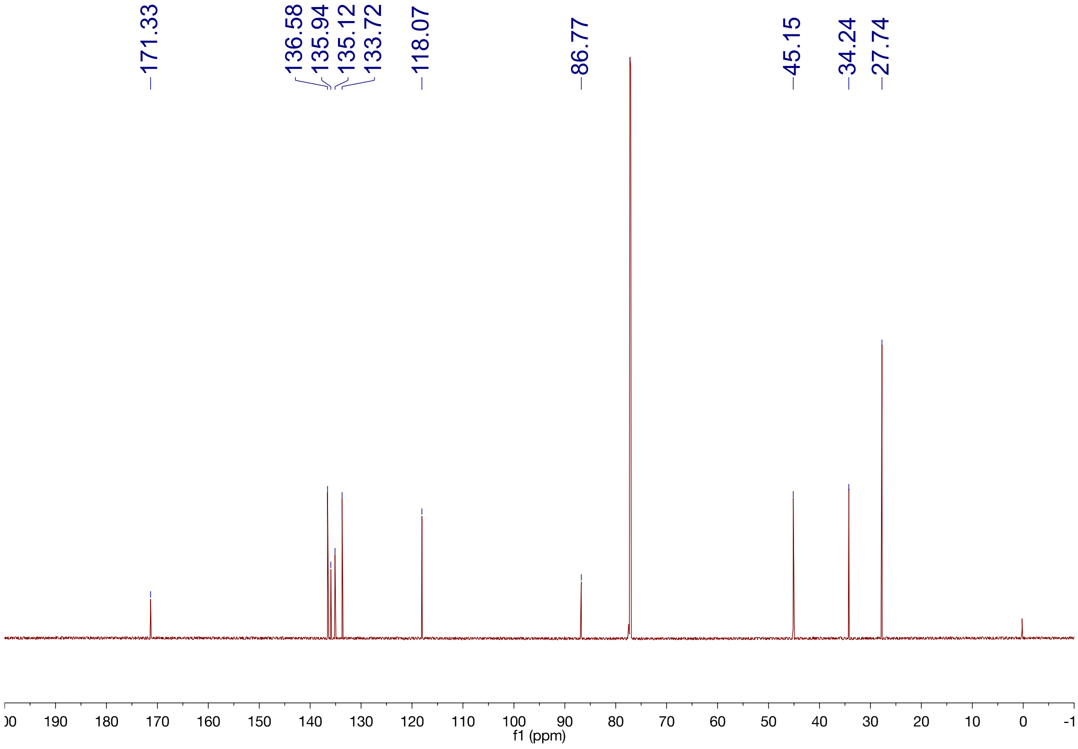

6-Iodo-4,4-dimethyl-1-(propan-2-yl)-1,2,3,4-tetrahydroquinolin-2-one (20a)

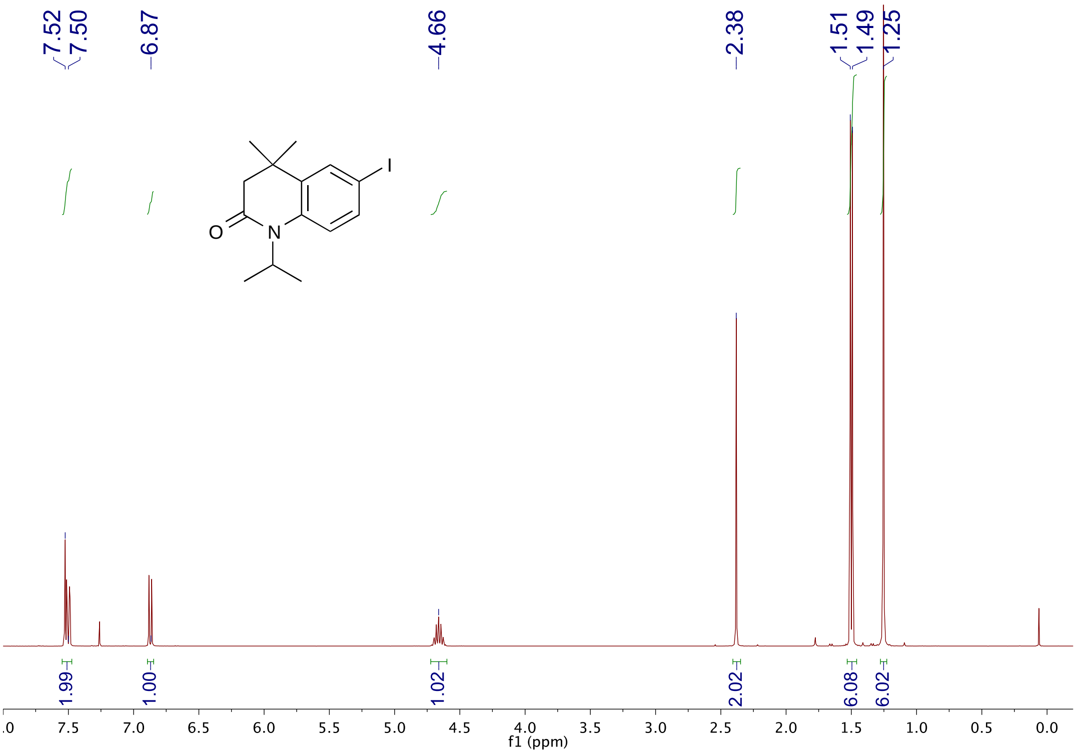

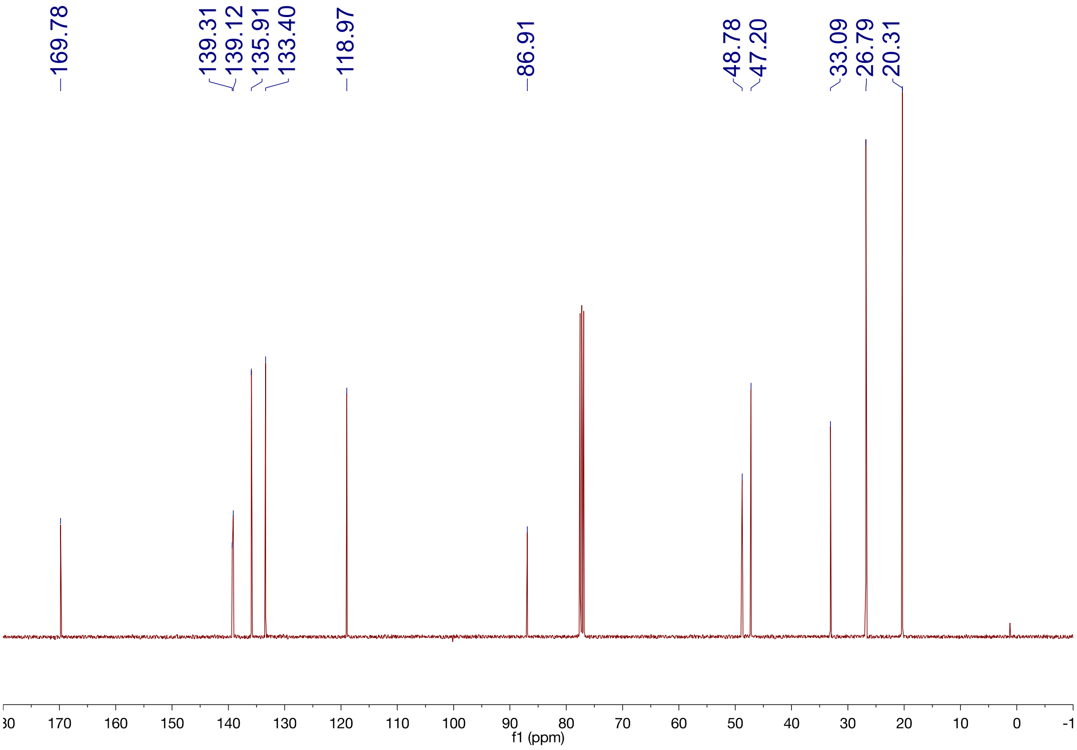

6-Iodo-4,4-dimethyl-2-(propan-2-yloxy)-3,4-dihydroquinoline (20b)

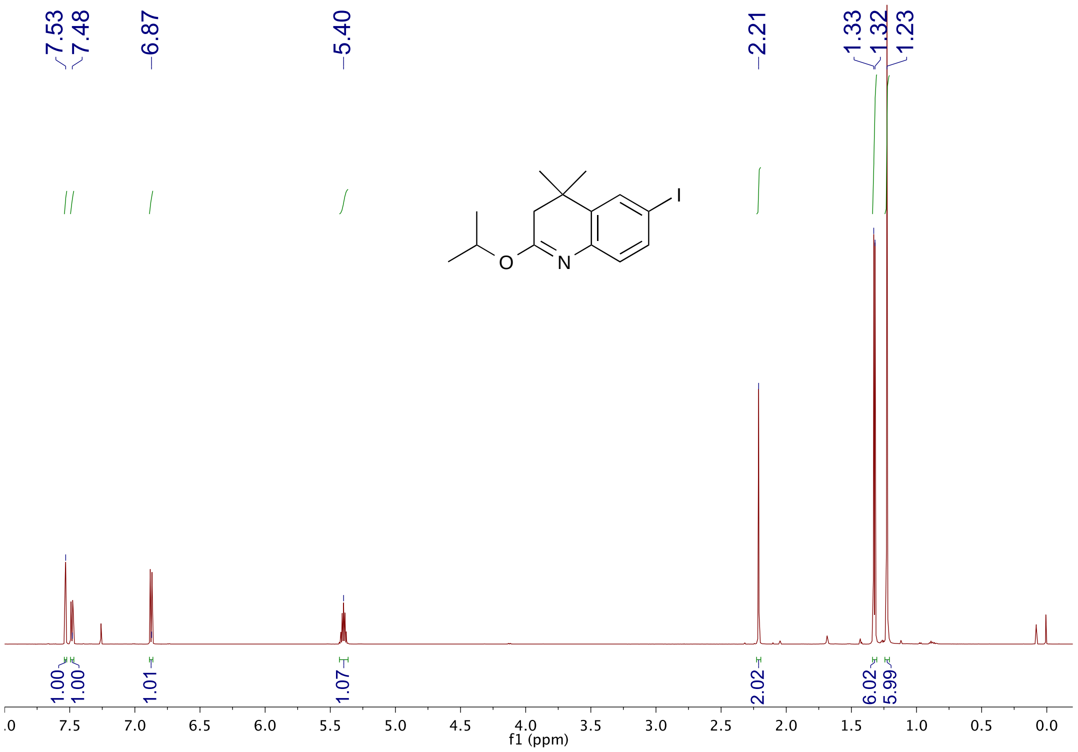

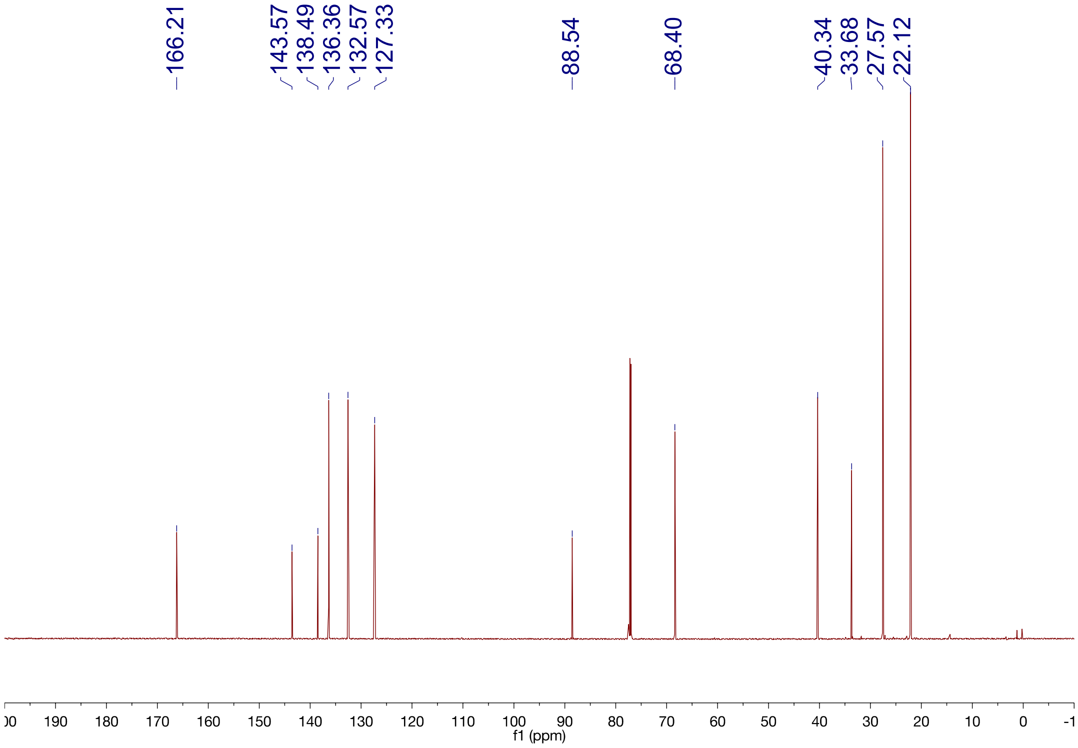

6-Iodo-4,4-dimethyl-1-(propan-2-yl)-1,2,3,4-tetrahydroquinoline (21)

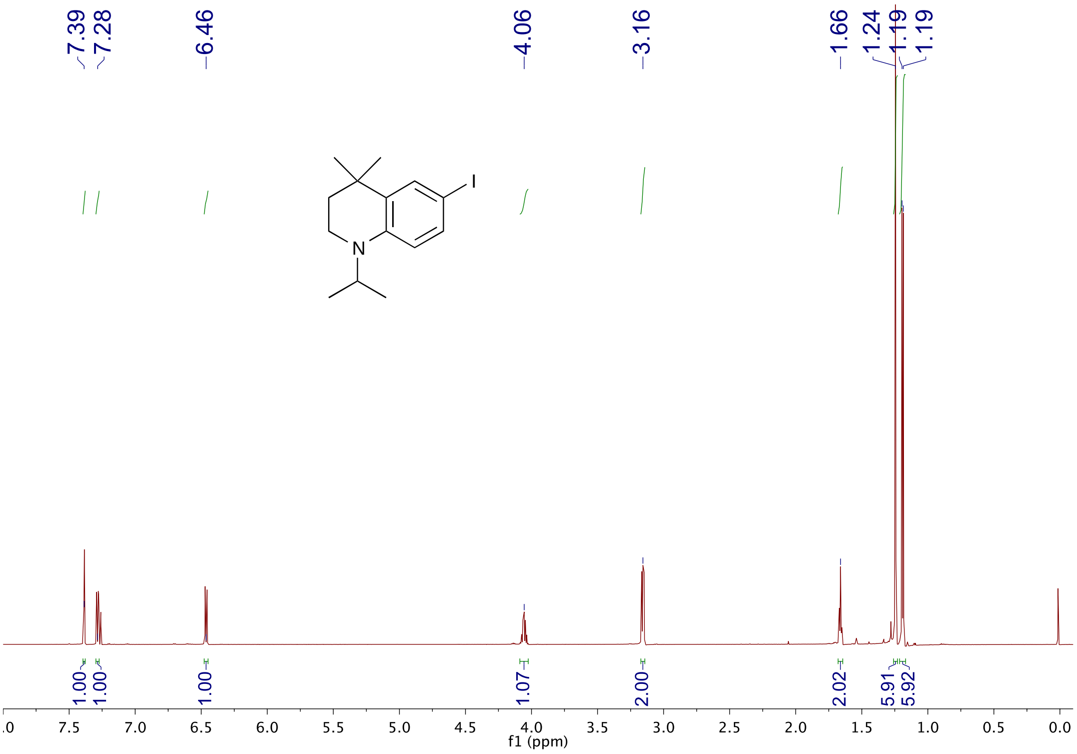

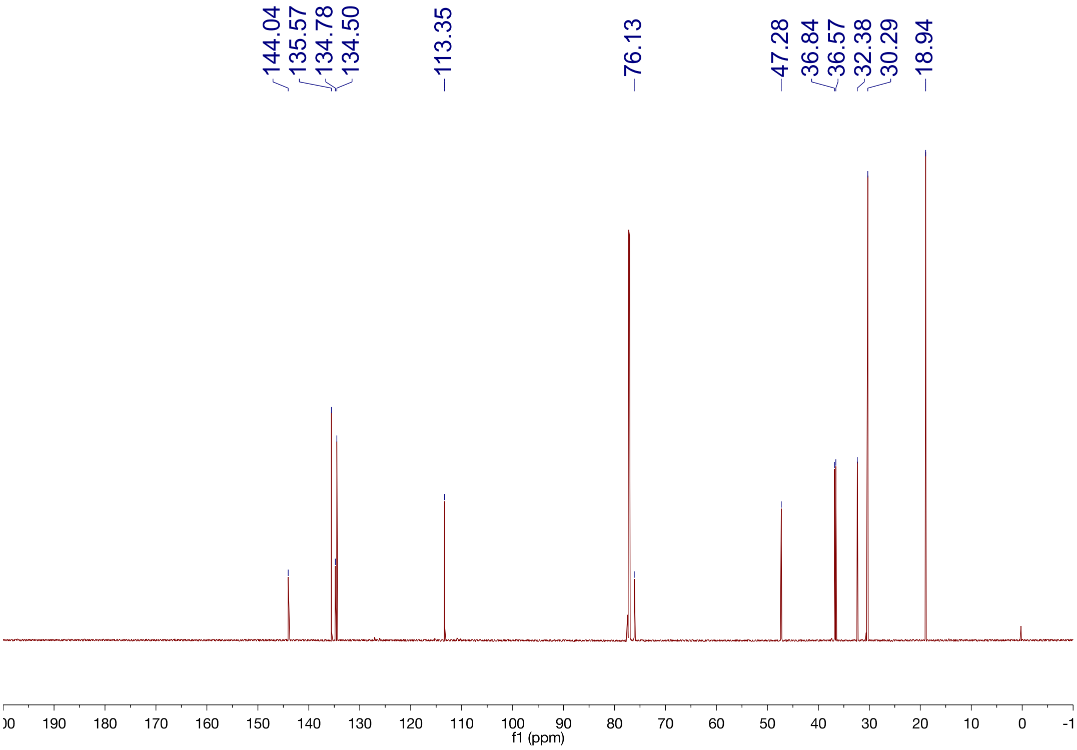

6-Iodo-4,4-dimethyl-1-(propan-2-yl)-1,4-dihydroquinoline (22)

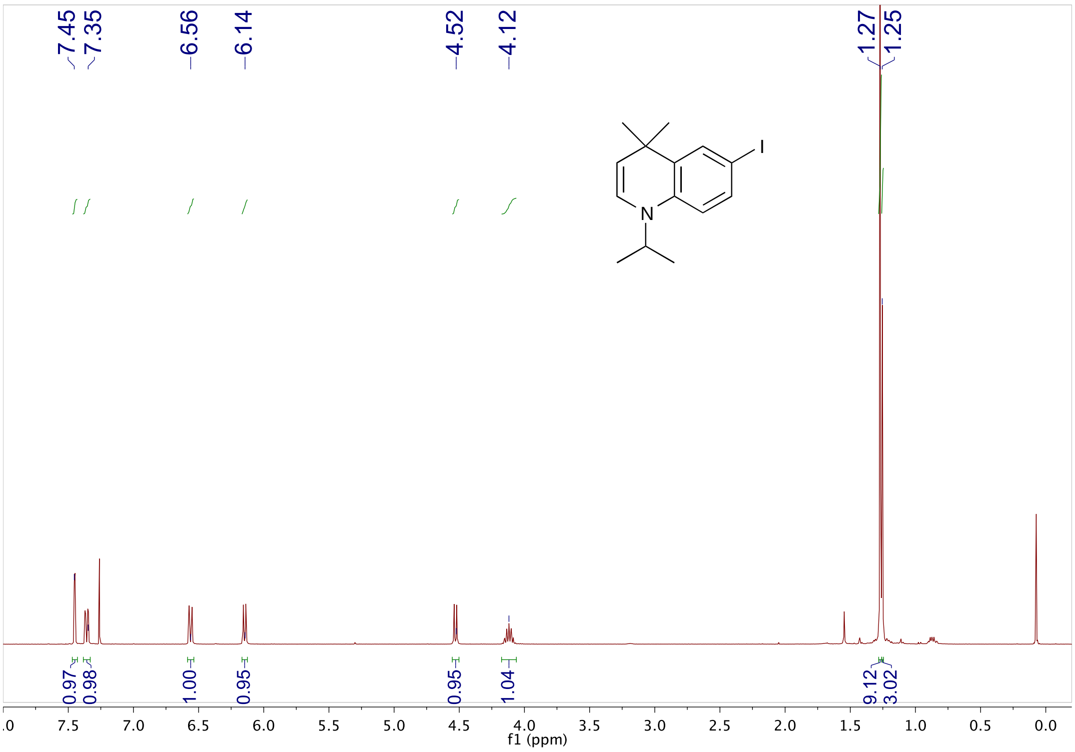

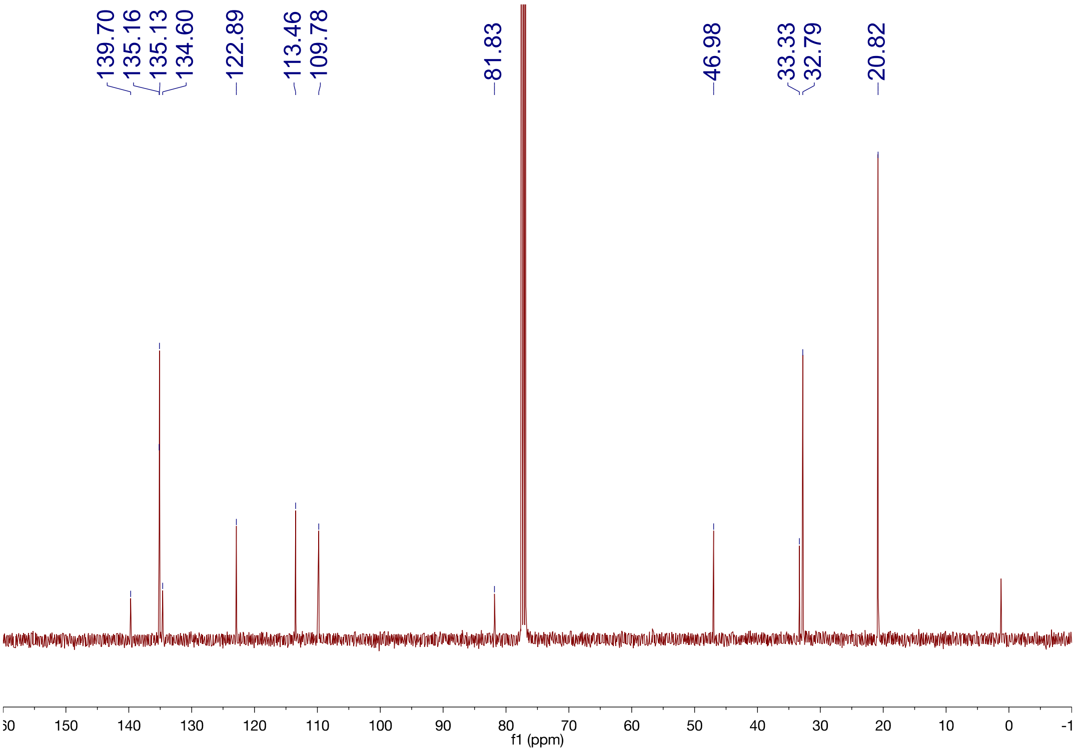

4-Phenyl-1,2,3,4-tetrahydroquinolin-2-one (23a)

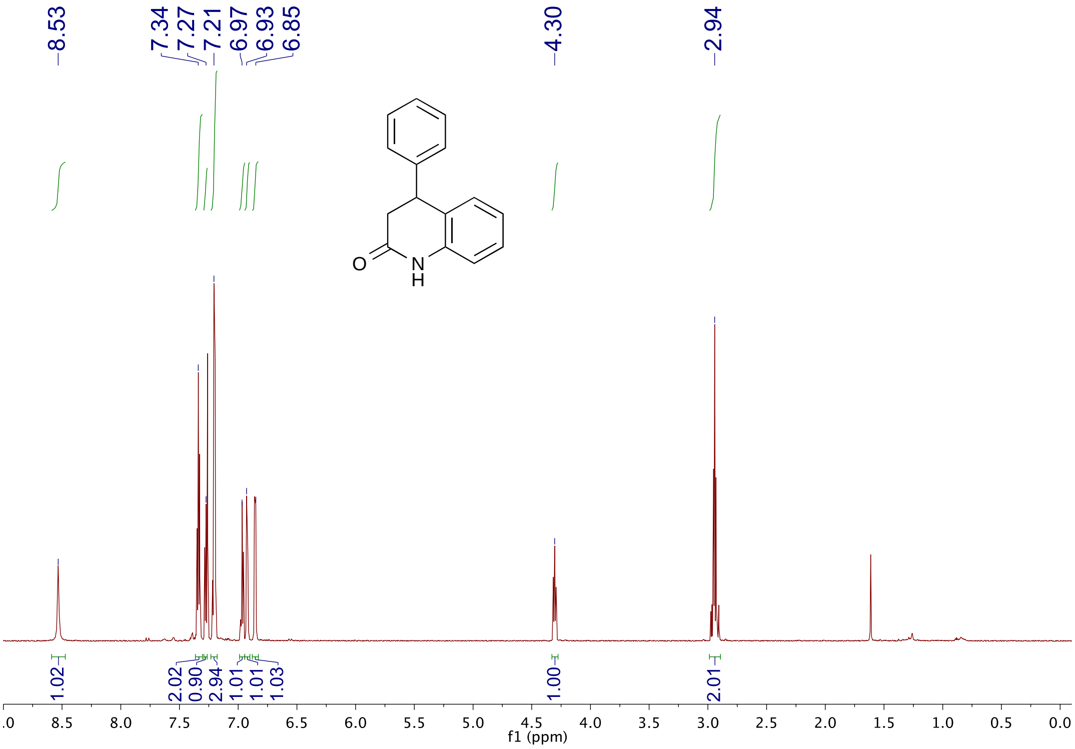

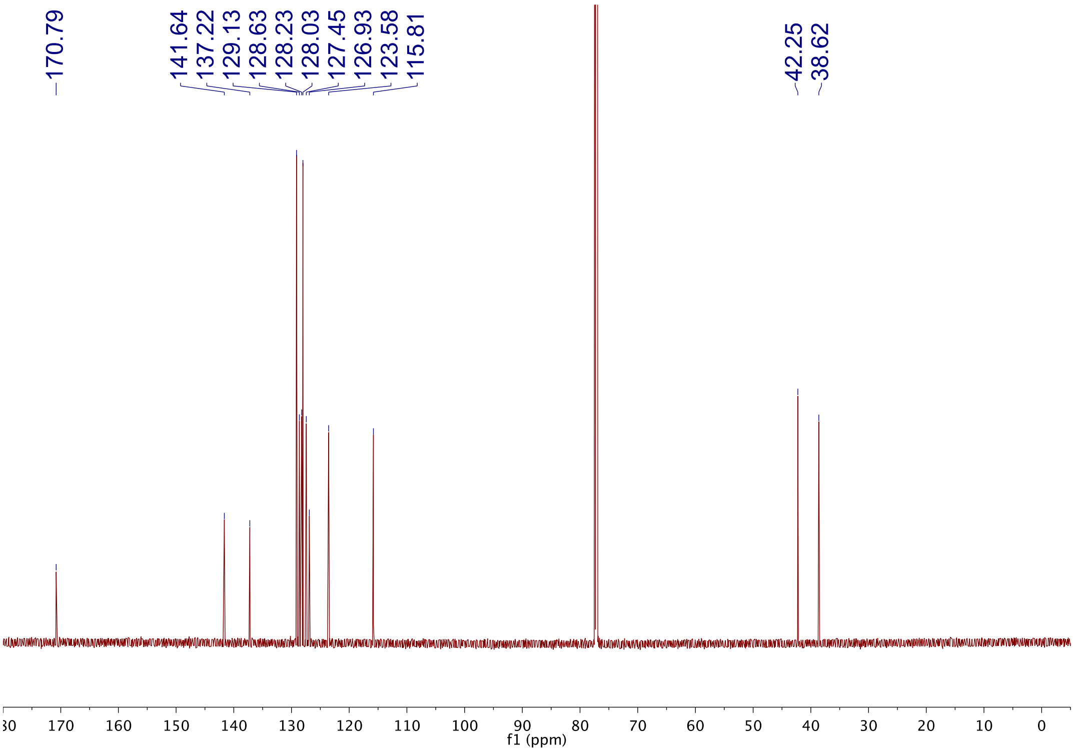

1-Methyl-4-phenyl-1,2,3,4-tetrahydroquinolin-2-one (23b)

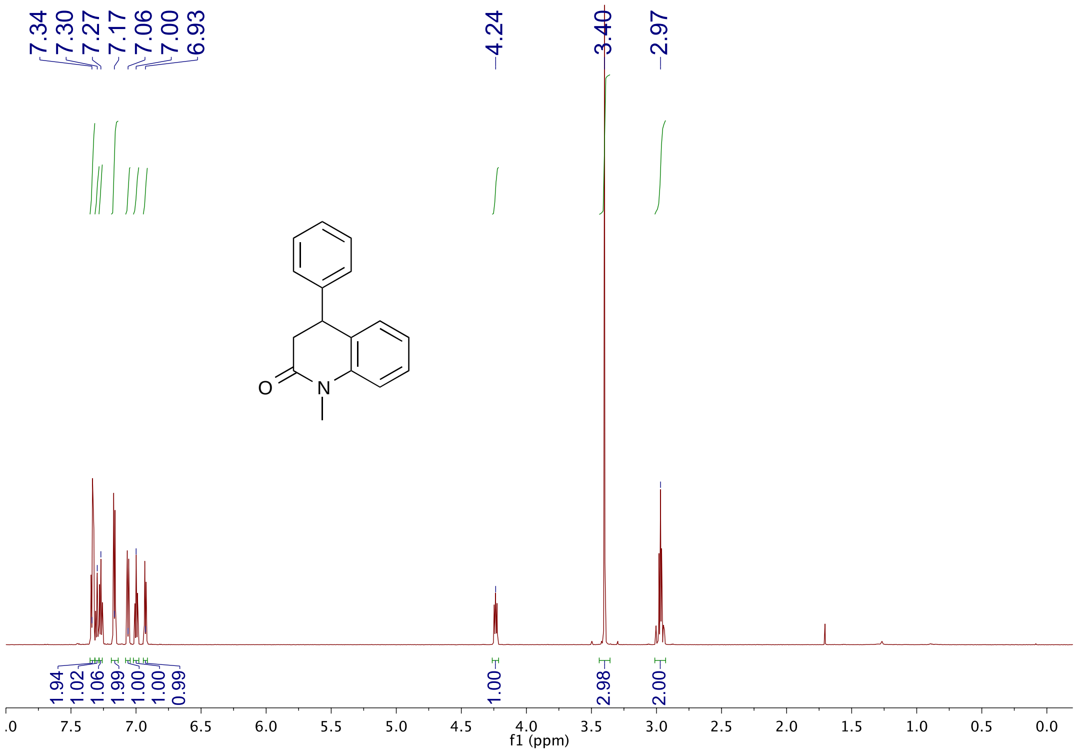

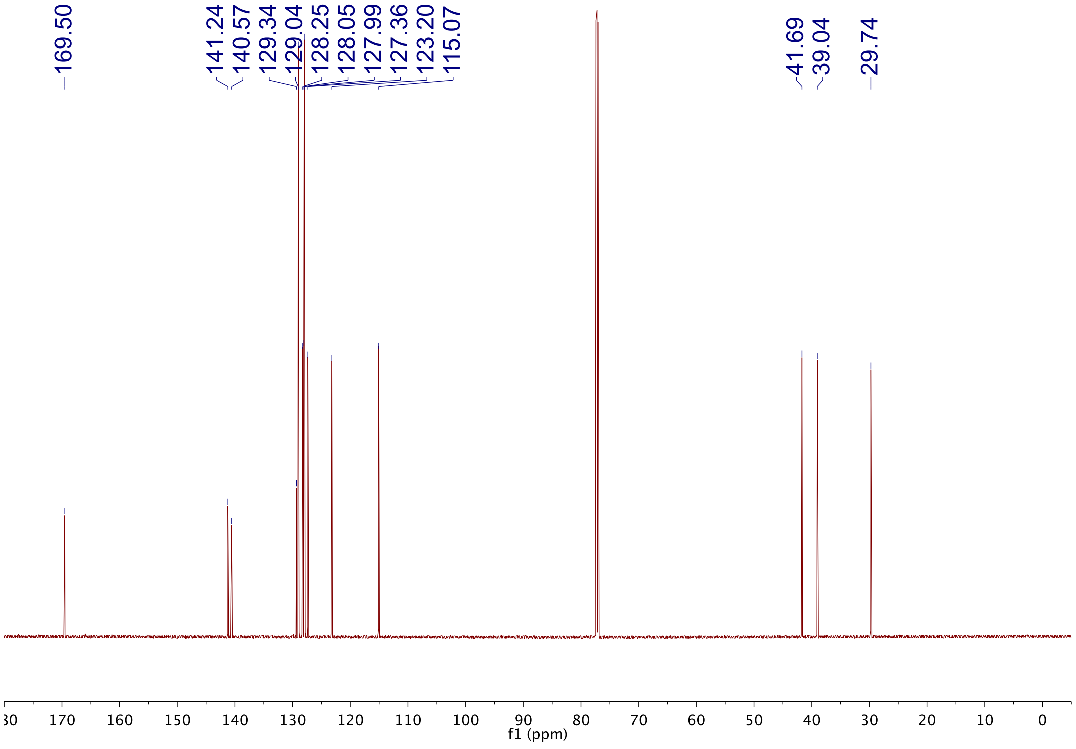

1-Ethyl-1,2,3,4-tetrahydroquinolin-2-one (23d)

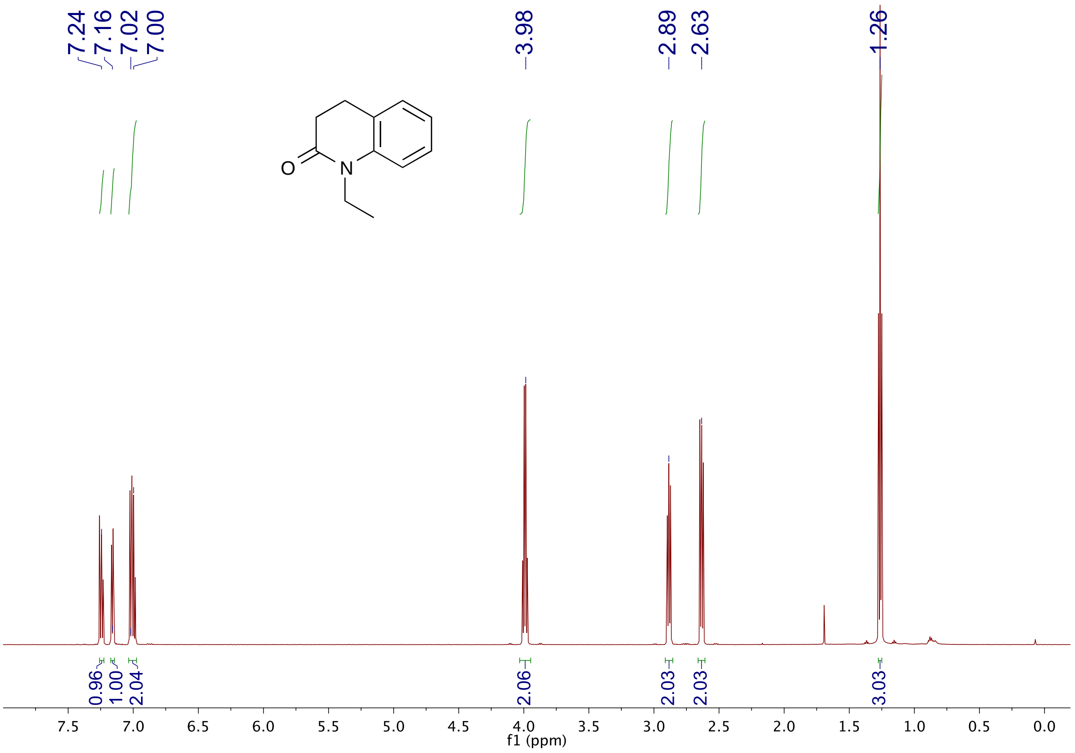

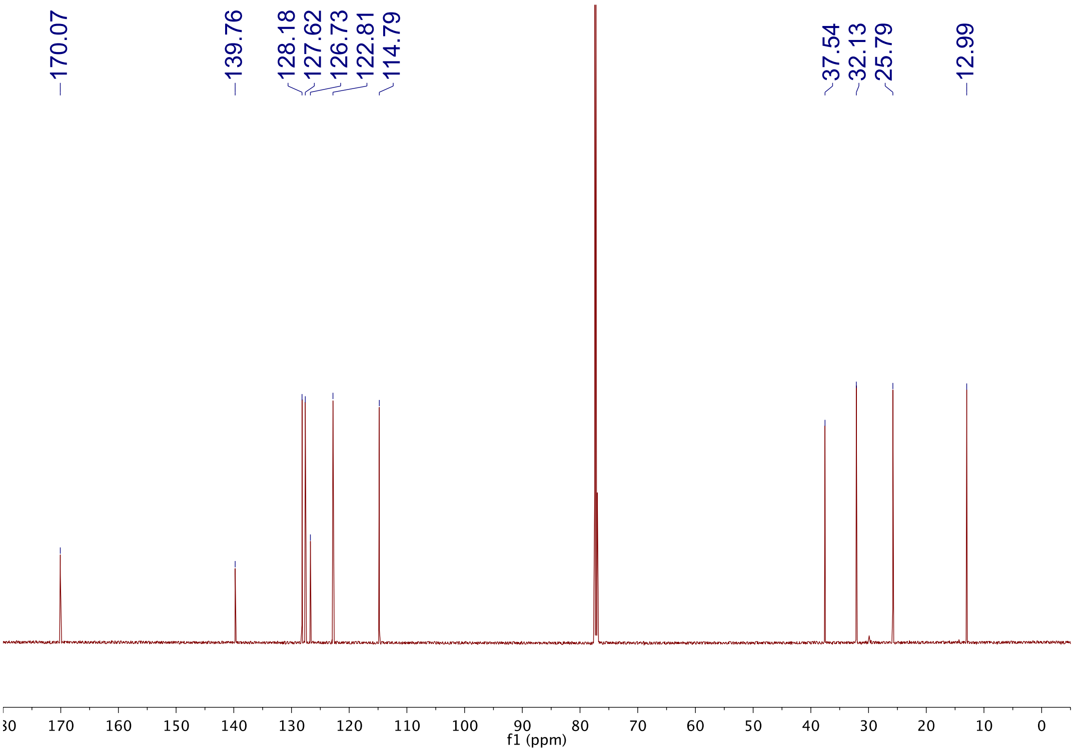

1-Benzyl-1,2,3,4-tetrahydroquinolin-2-one (23e)

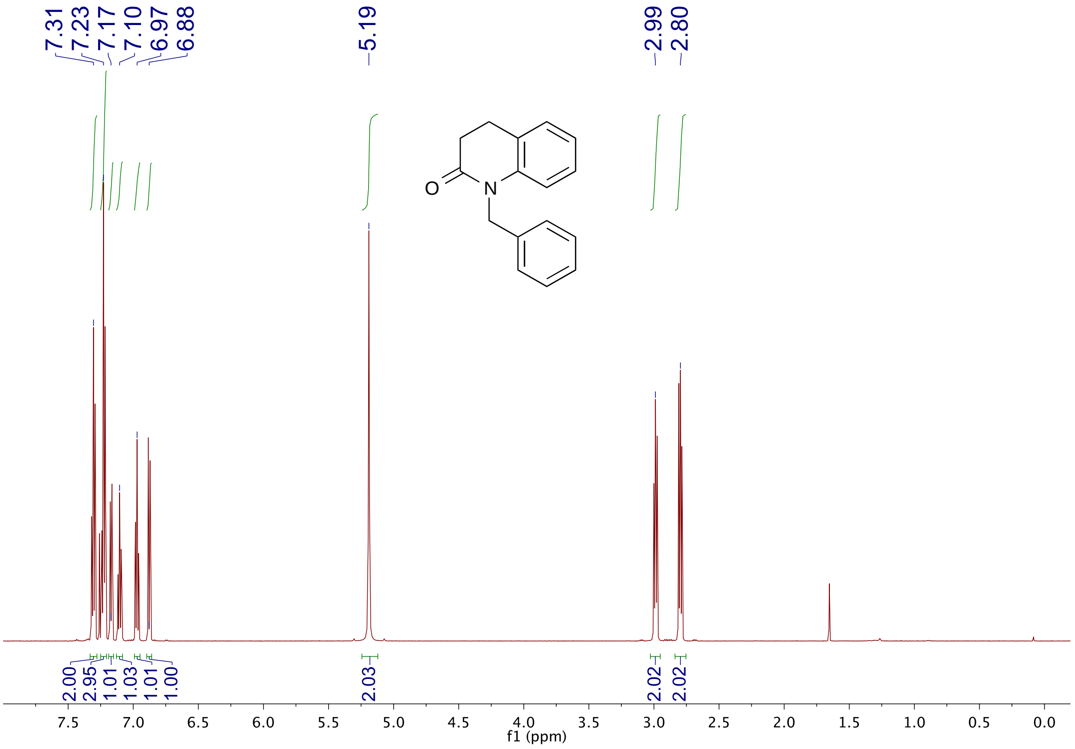

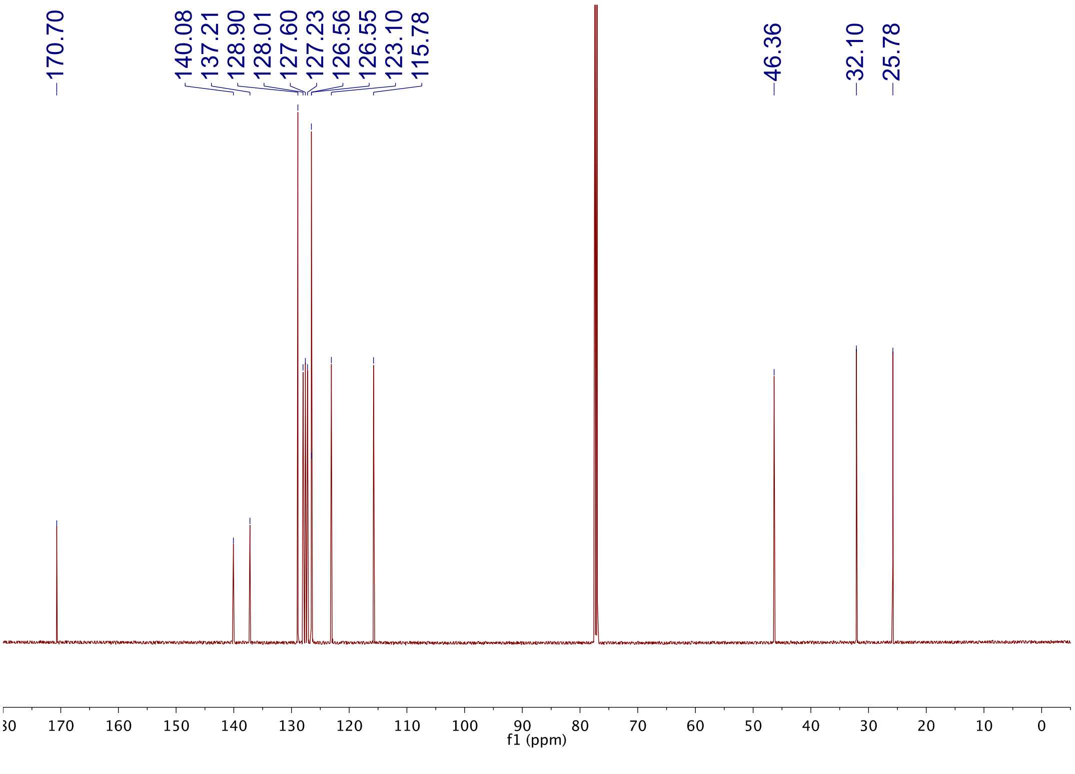

6-Bromo-4,4-dimethyl-1-(propan-2-yl)-1,2,3,4-tetrahydroquinolin-2-one

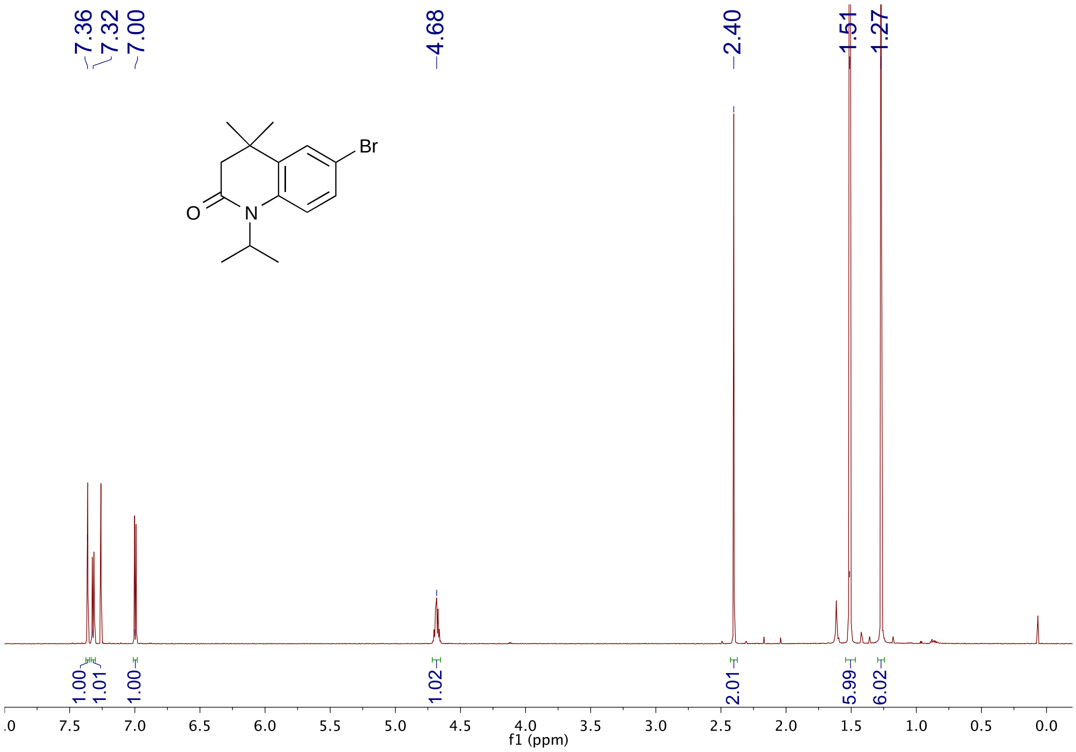

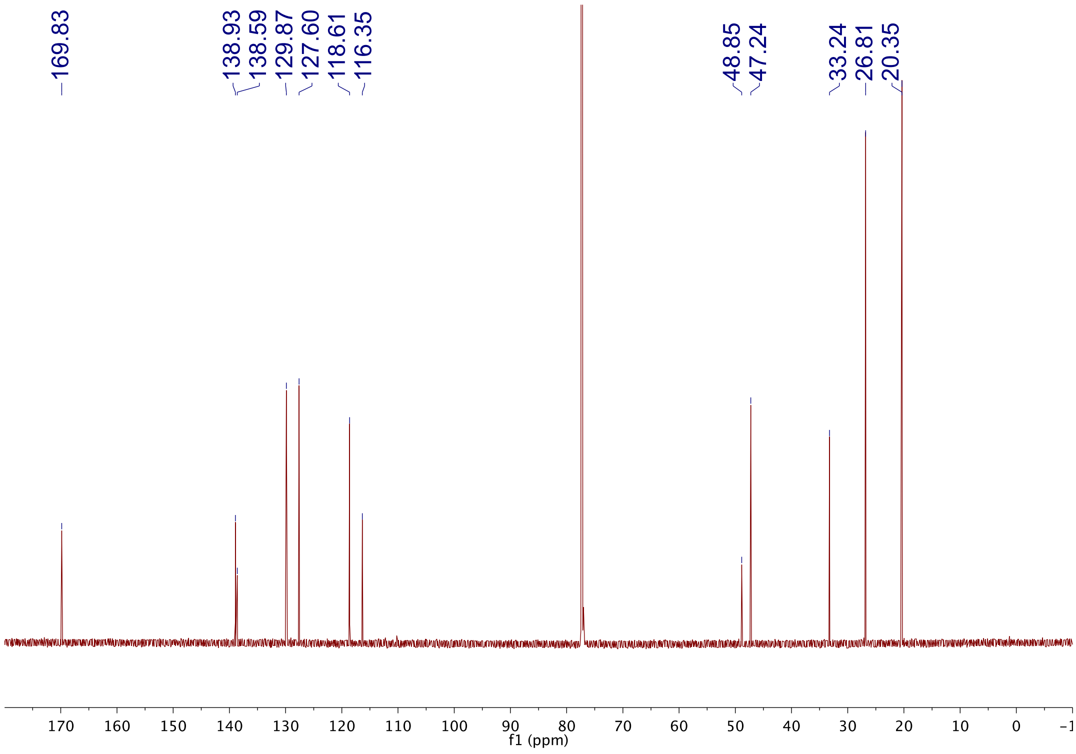

6-Bromo-4,4-dimethyl-1-(propan-2-yl)-1,2,3,4-tetrahydroquinoline (26)

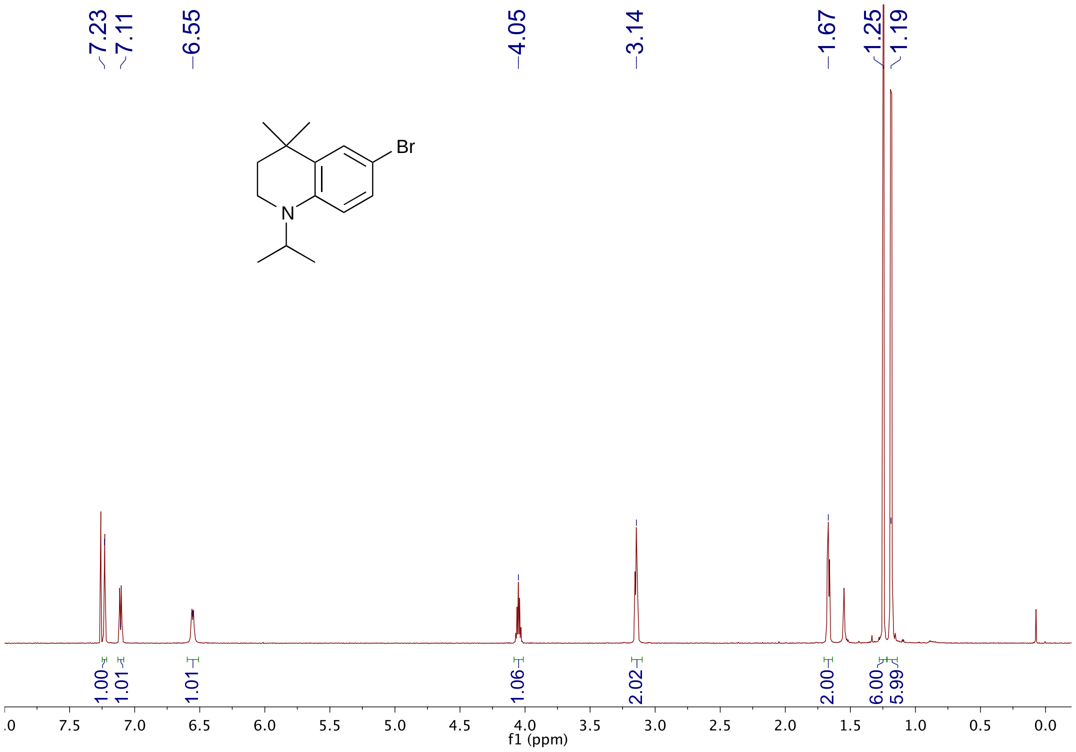

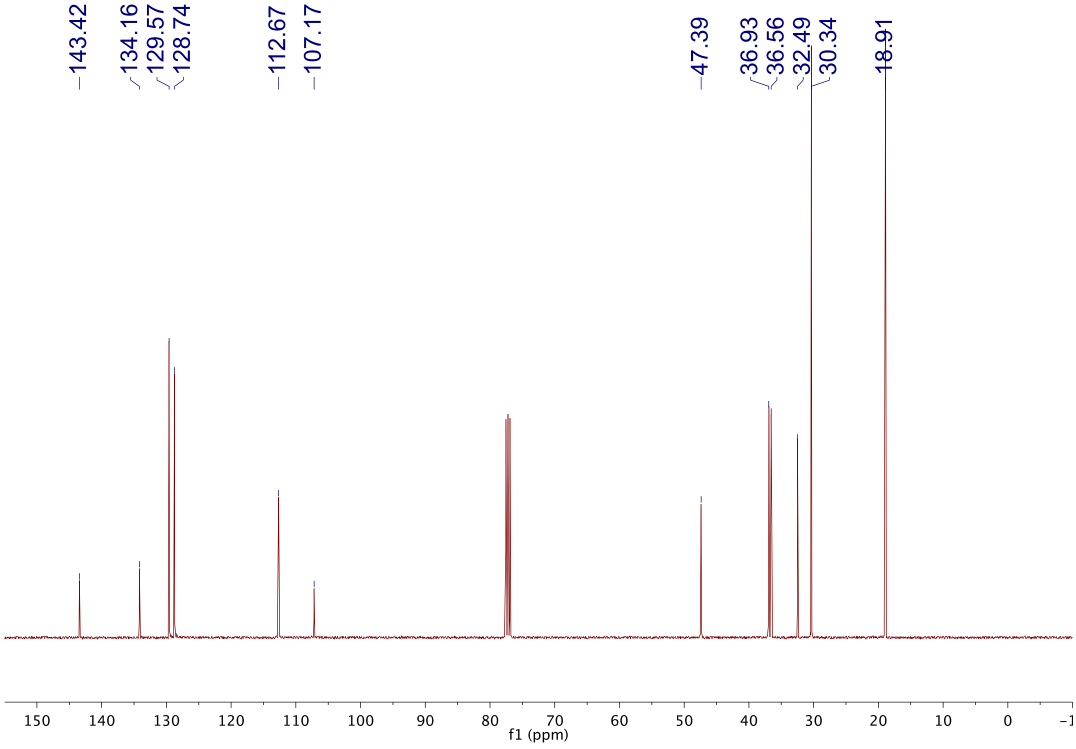

Methyl 4-(tetramethyl-1,3,2-dioxaborolan-2-yl)benzoate (27)

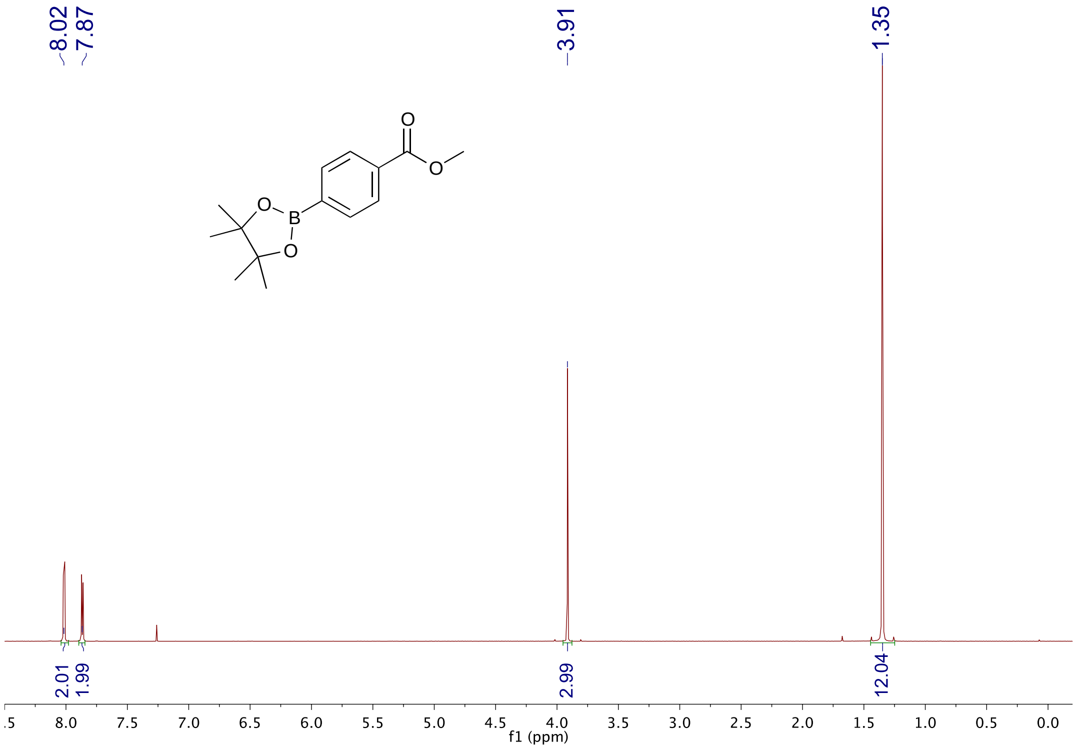

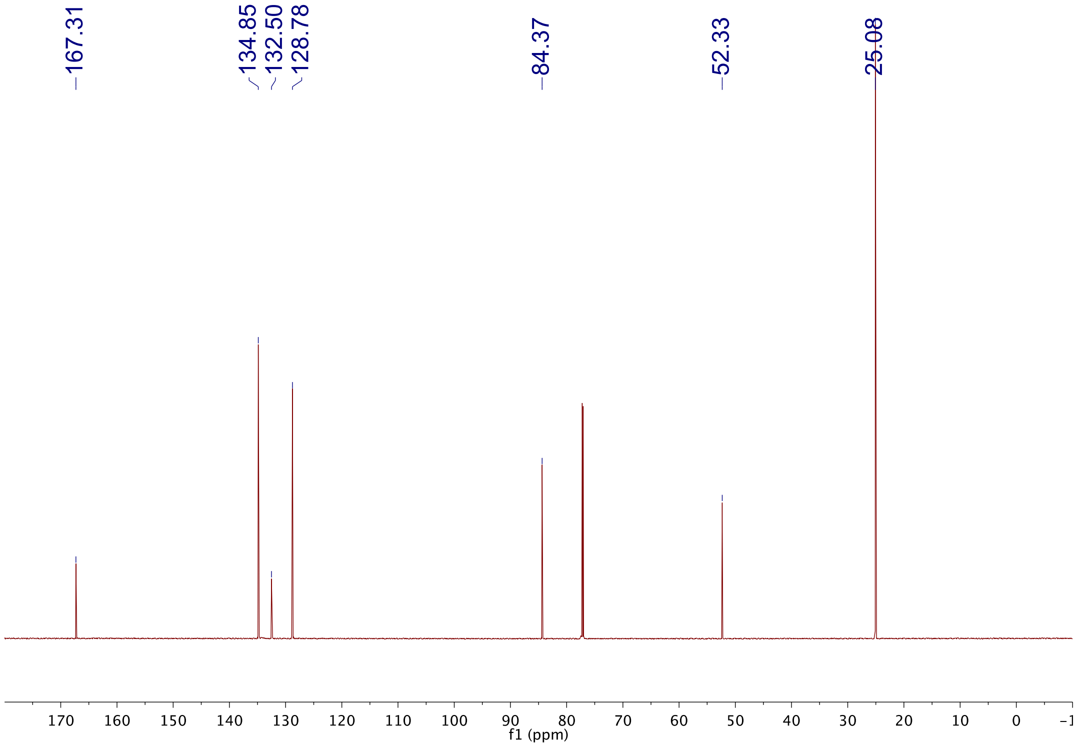

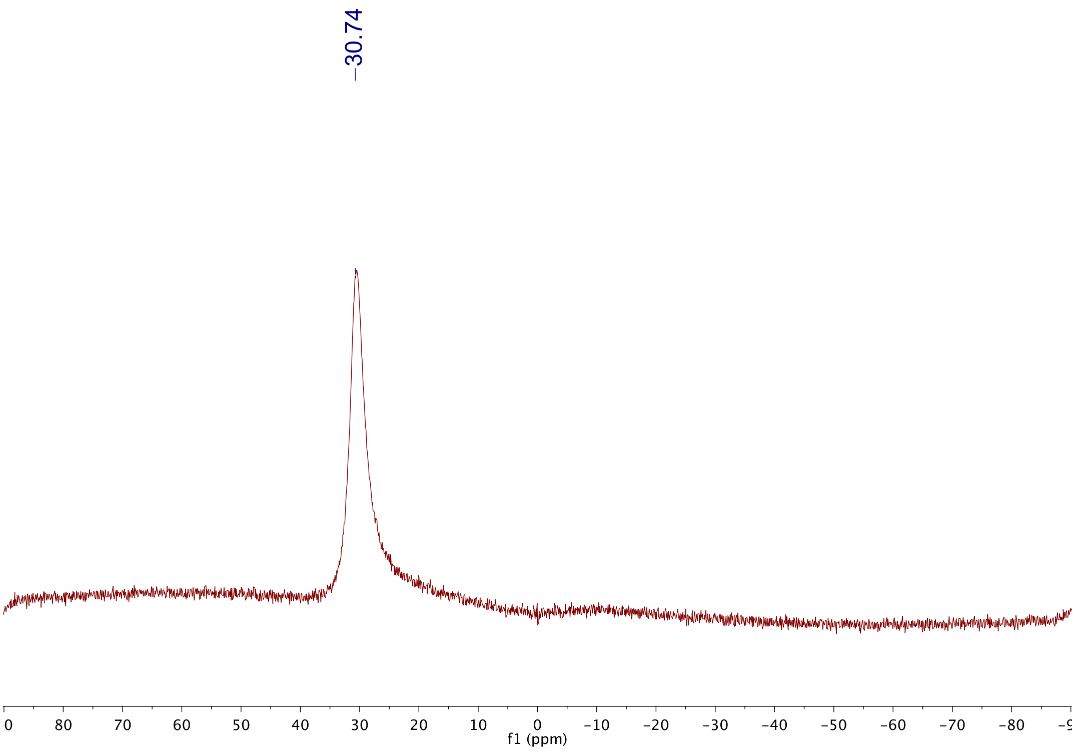

30.74
0
80
70
60
50
40
30
20
10
0
-10
-20
-30
-40
-50
-60
-70
-80
f1 (ppm)

Methyl 4-[4,4-dimethyl-1-(propan-2-yl)-1,2,3,4-tetrahydroquinolin-6-yl]benzoate (28)

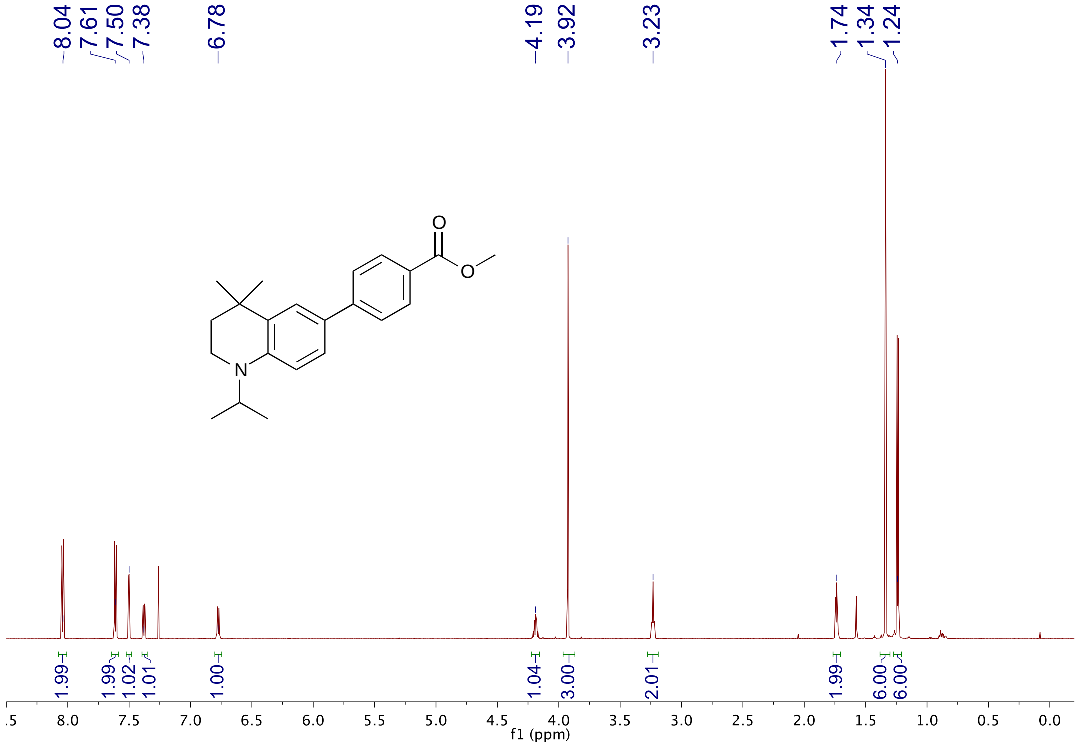

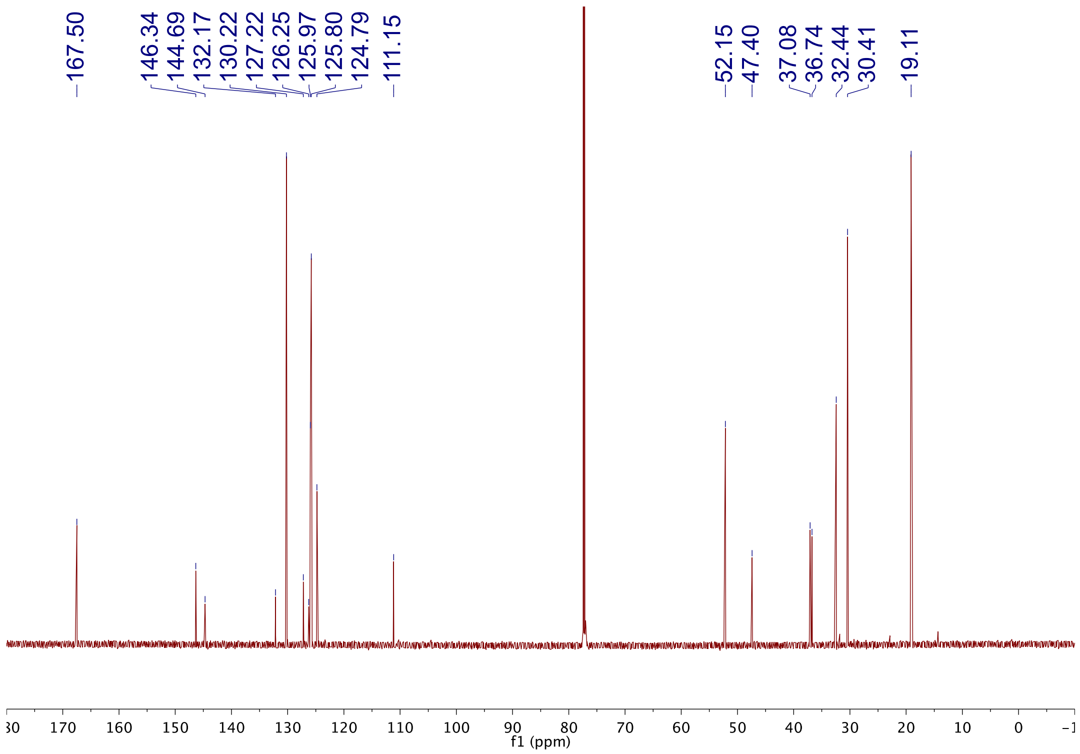

4,4-Dimethyl-1-(propan-2-yl)-6-(tetramethyl-1,3,2-dioxaborolan-2-yl)-1,2,3,4-tetrahydroquinoline (29)

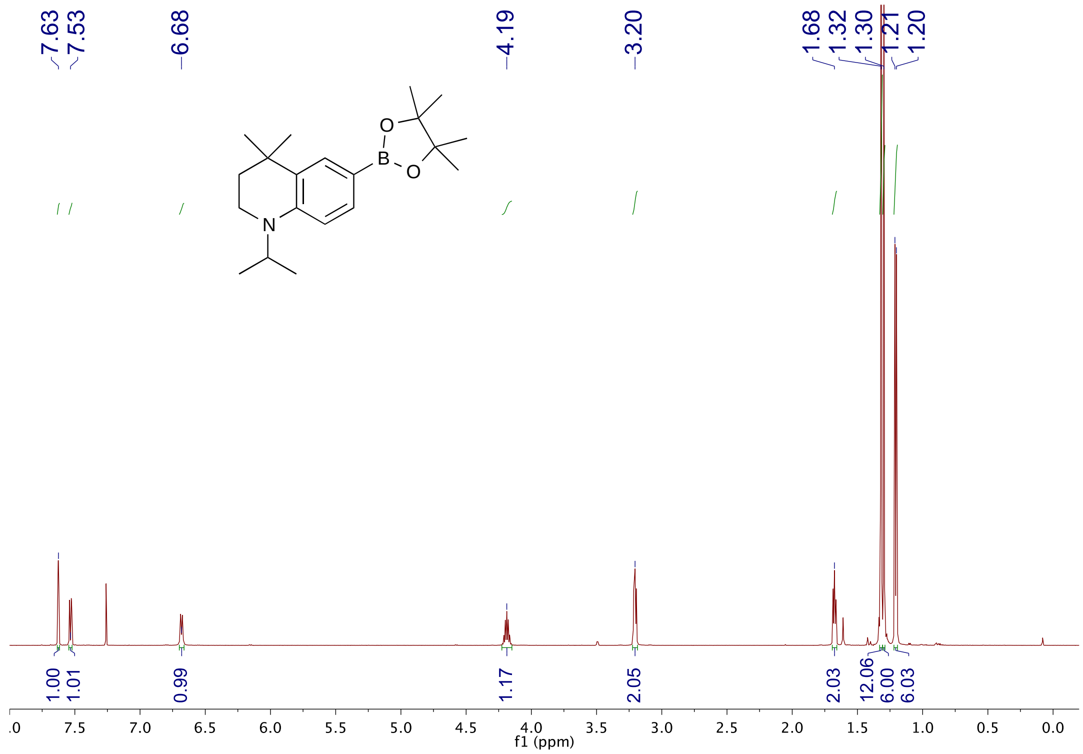

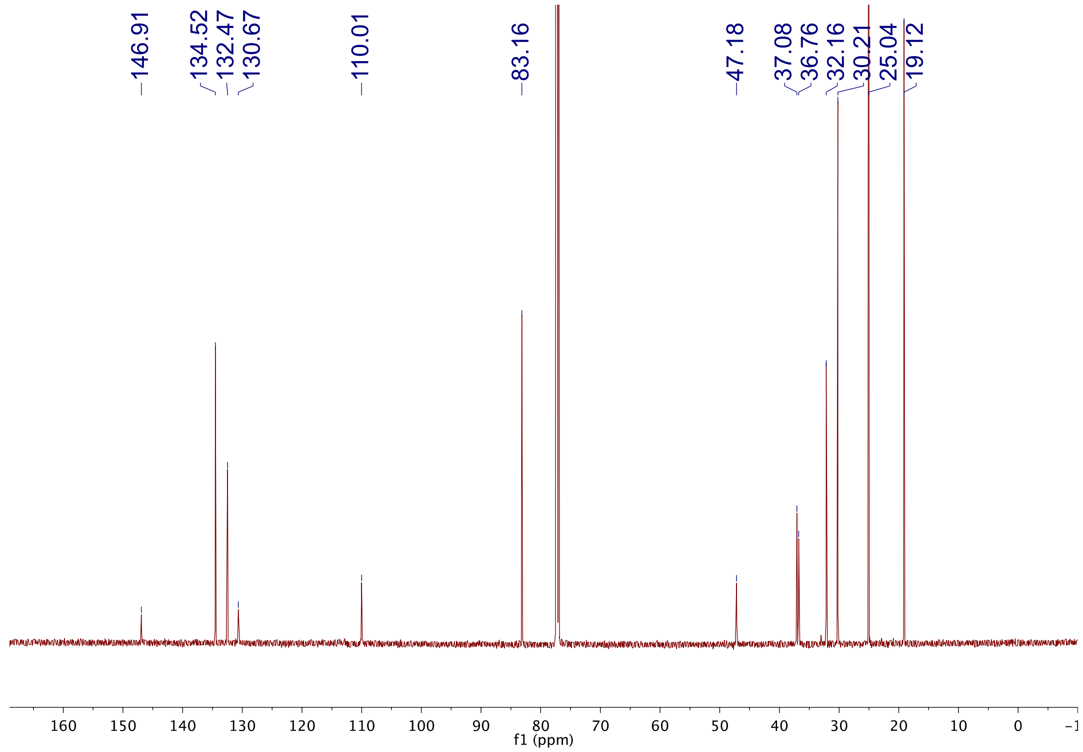

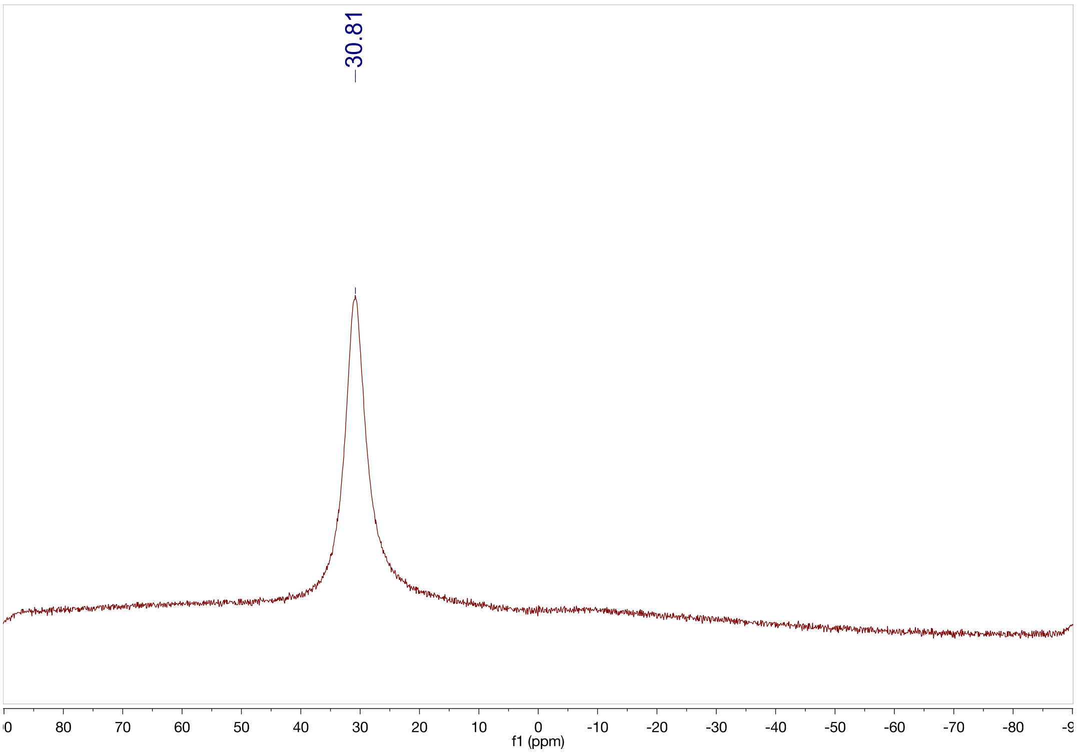

—30.81
80
70
60
50
40
30
20
10
0
-10
-20
-30
-40
-50
-60
-70
-80
f1 (ppm)

4,4-Dimethyl-1-(propan-2-yl)-6-(tetramethyl-1,3,2-dioxaborolan-2-yl)-1,4-dihydroquinoline (30)

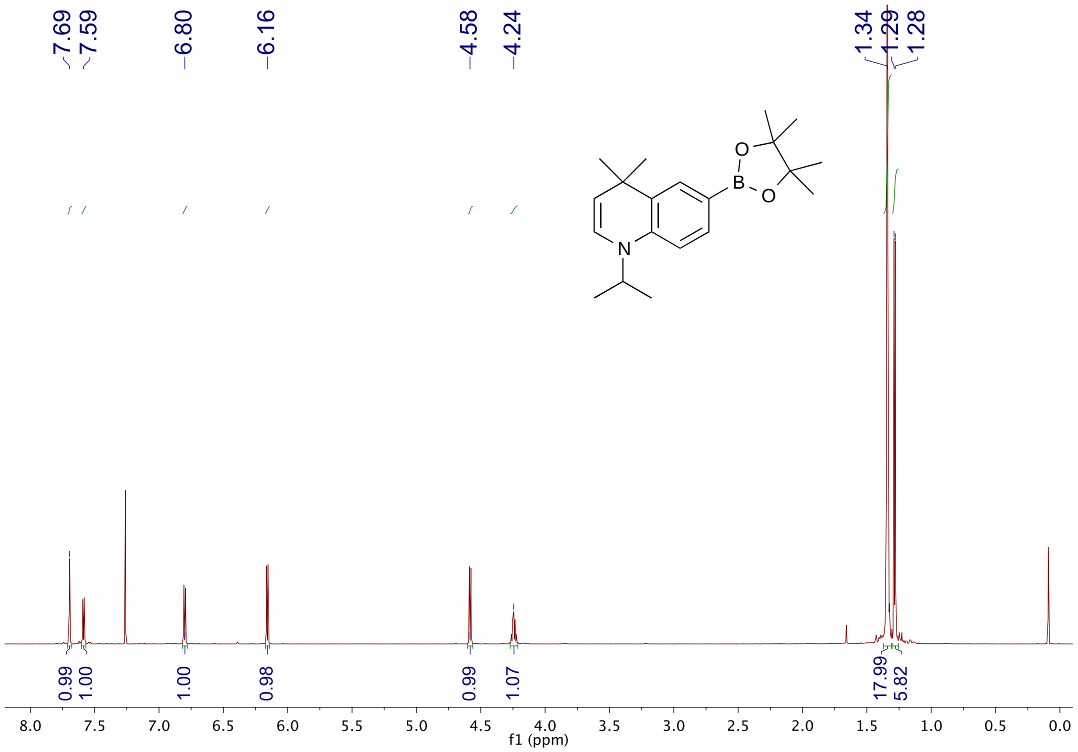

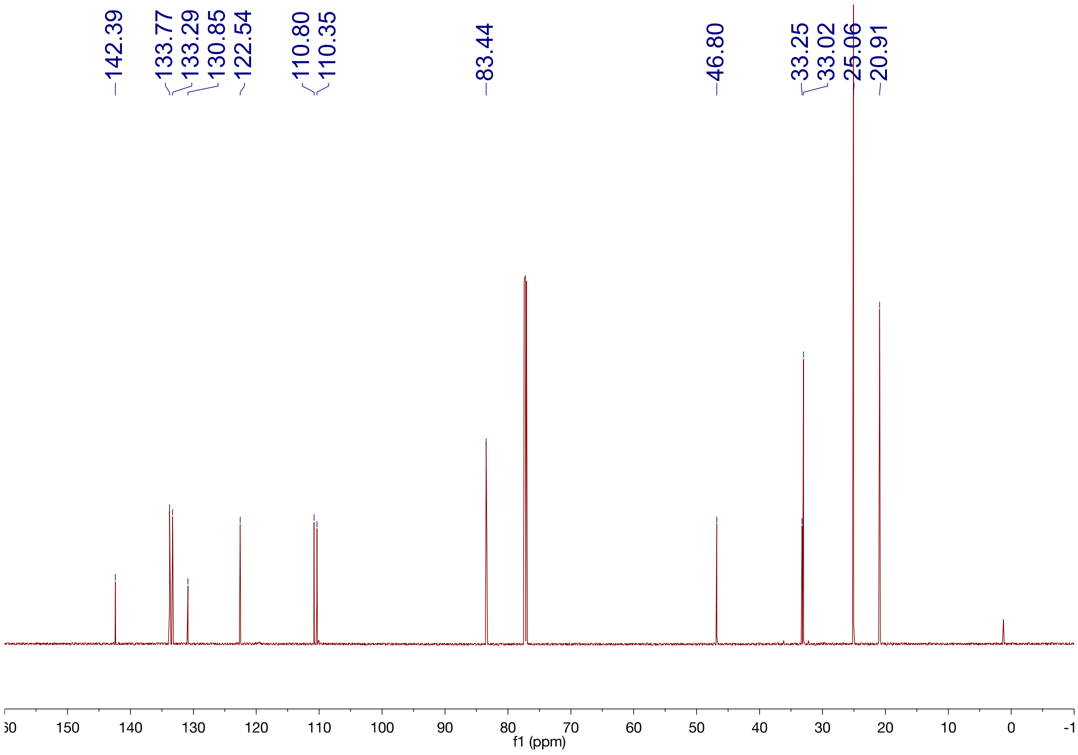

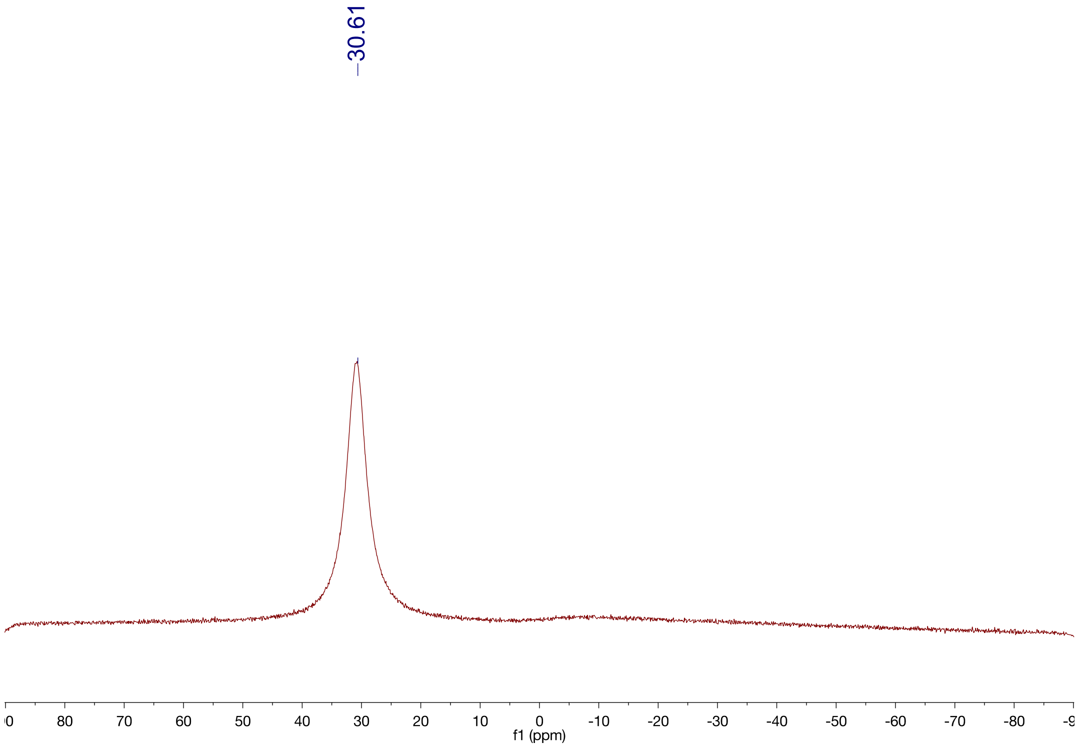

30.61
80
70
60
50
40
30
20
10
0
-10
-20
-30
-40
-50
-60
-70
-80
f1 (ppm)

5. X-ray crystallography

6-Iodo-4,4,8-trimethyl-1,2,3,4-tetrahydroquinolin-2-one (13)

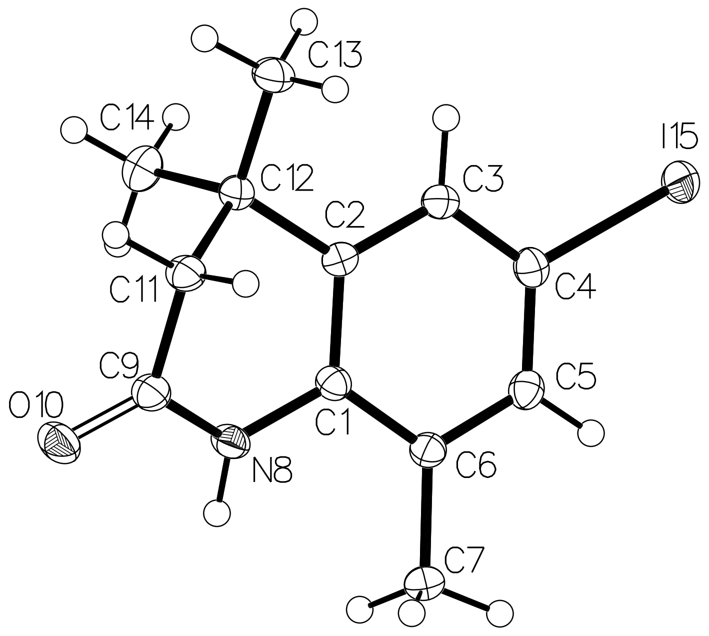

Figure S9: X-ray molecular structure of compound 13. Thermal ellipsoids are drawn at the 50% probability level. Selected bond distances (Å): C(4)-I(15) 2.101, C(1)-N(8) 1.402, C(9)-N(8) 1.352, C(9)-O(10) 1.229, C(9)-C(11) 1.505.

6-Iodo-4,4,8-trimethyl-1,2,3,4-tetrahydroquinoline (14)

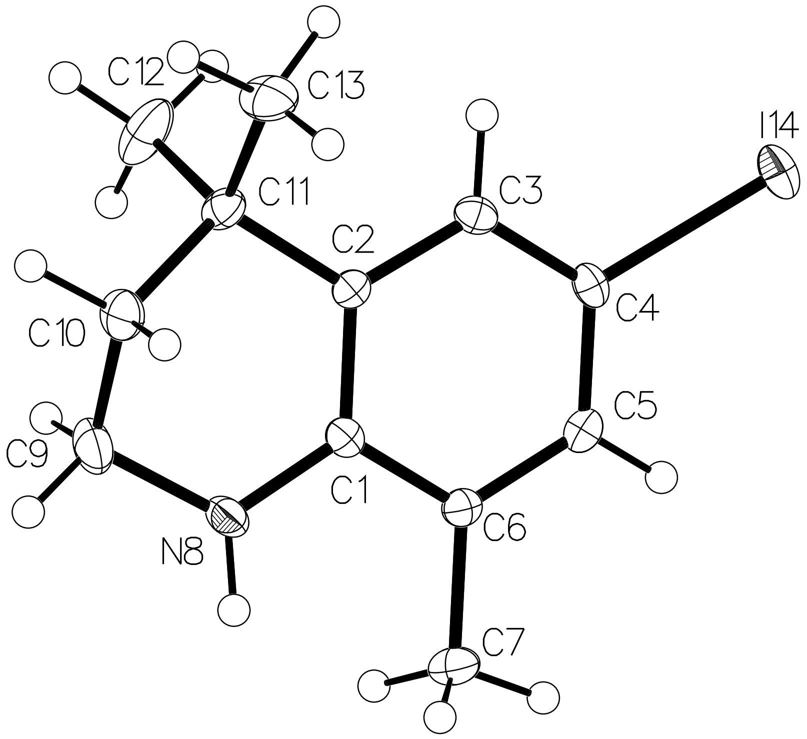

Figure S10: X-ray molecular structure of compound 14. Anisotropic displacement ellipsoids are drawn at the 50% probability level. Selected bond distances (Å): C(4)-I(14) 2.103, C(1)-N(8) 1.384, C(9)-N(8) 1.446, C(9)-C(10) 1.516. RMSD between the two molecules in the asymmetric unit (Å): 0.345 (without inversion), 0.990 (with inversion).

6-Iodo-4,4,8-trimethyl-2-(propan-2-yloxy)-3,4-dihydroquinoline (15b)

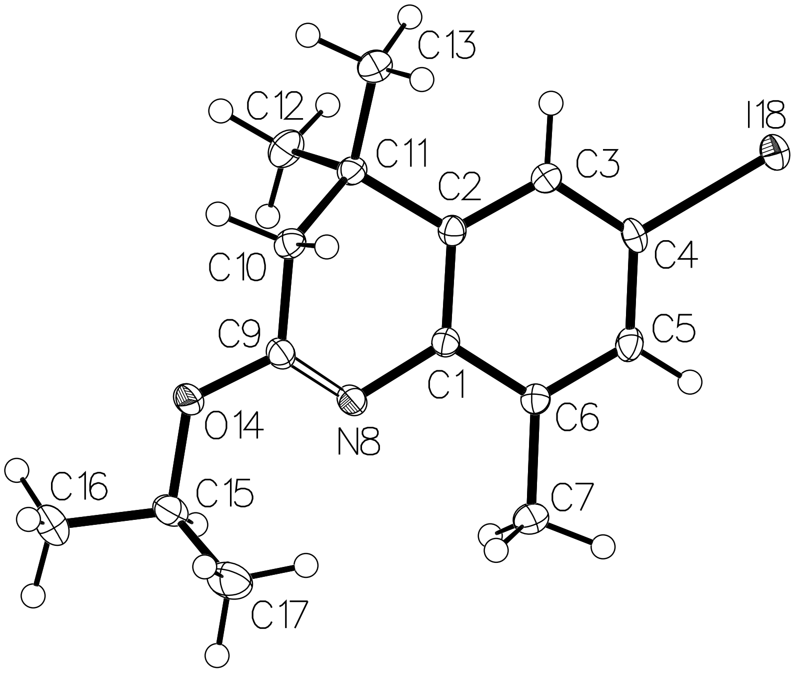

Figure S11: X-ray molecular structure of compound 15b. Thermal ellipsoids are drawn at the 50% probability level. Selected bond distances (Å): C(4)-I(18) 2.100, C(1)-N(8) 1.412, C(9)-N(8) 1.285, C(9)-O(14) 1.334, C(15)-O(14) 1.456.

6-Iodo-4,4-dimethyl-1,2,3,4-tetrahydroquinolin-2-one (19)

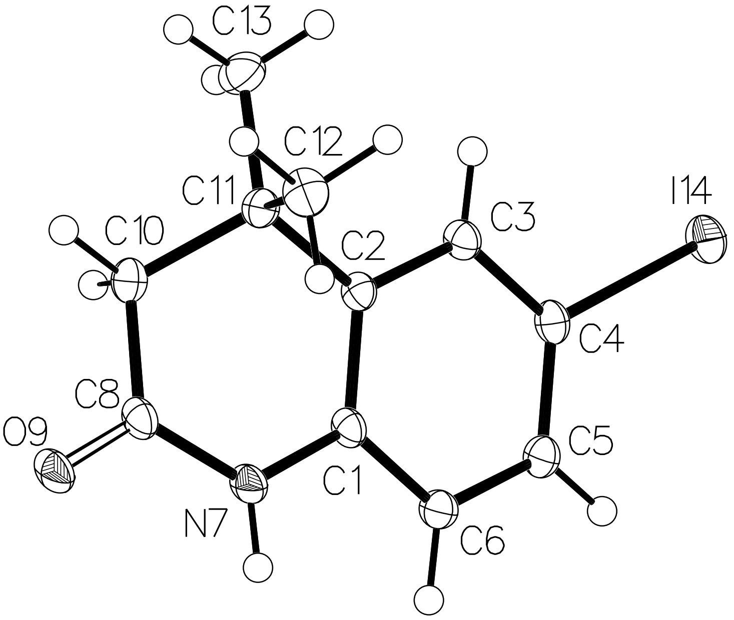

Figure S12: X-ray molecular structure of compound 19. Thermal ellipsoids are drawn at the 50% probability level. Selected bond distances (Å): C(4)-I(14) 2.098, C(1)-N(7) 1.404, C(8)-N(7) 1.356, C(8)-O(9) 1.237, C(8)-C(10) 1.503.

4,4-Dimethyl-1-(propan-2-yl)-6-(tetramethyl-1,3,2-dioxaborolan-2-yl)-1,2,3,4-tetrahydroquinoline (29)

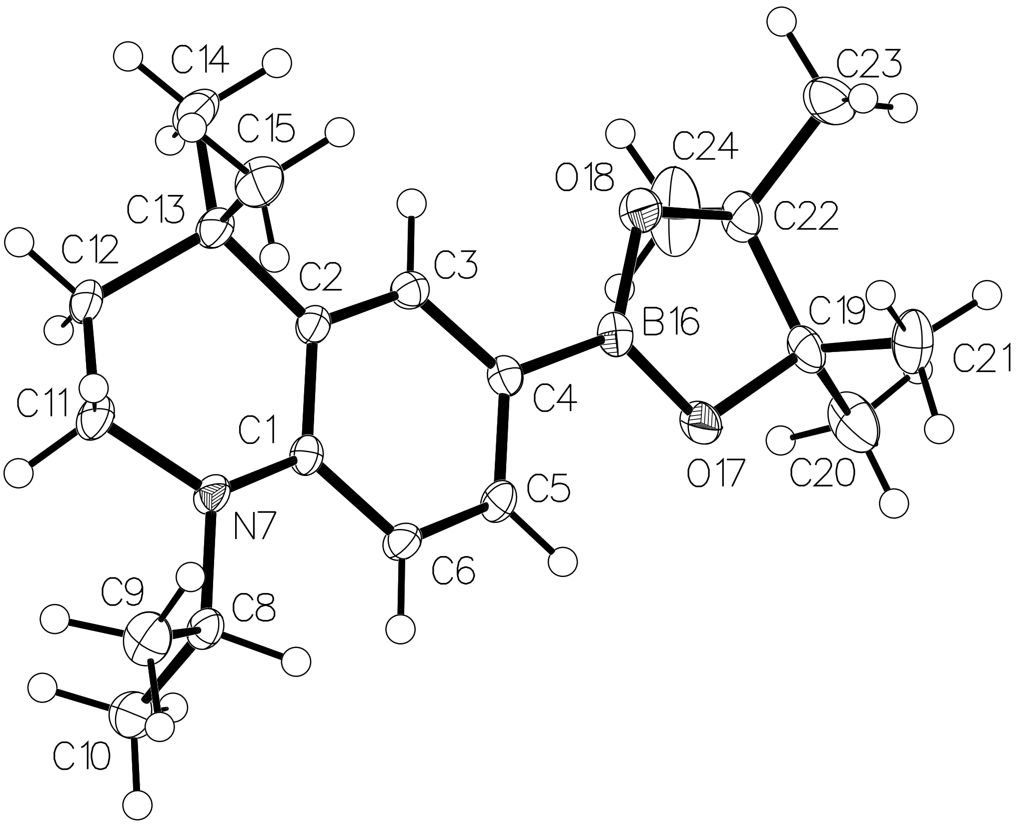

Figure S13: X-ray molecular structure of compound 29 (one of two molecules in the asymmetric unit). Thermal ellipsoids are drawn at the 50% probability level. Selected bond distances (Å): C(4)-B(16) 1.545, C(1)-N(7) 1.377, C(8)-N(7) 1.467, N(7)-C(11) 1.458. RMSD between the two molecules in the asymmetric unit (Å): 1.281 (without inversion), 0.215 (with inversion).

4,4-Dimethyl-1-(propan-2-yl)-6-(tetramethyl-1,3,2-dioxaborolan-2-yl)-1,4-dihydroquinoline (30)

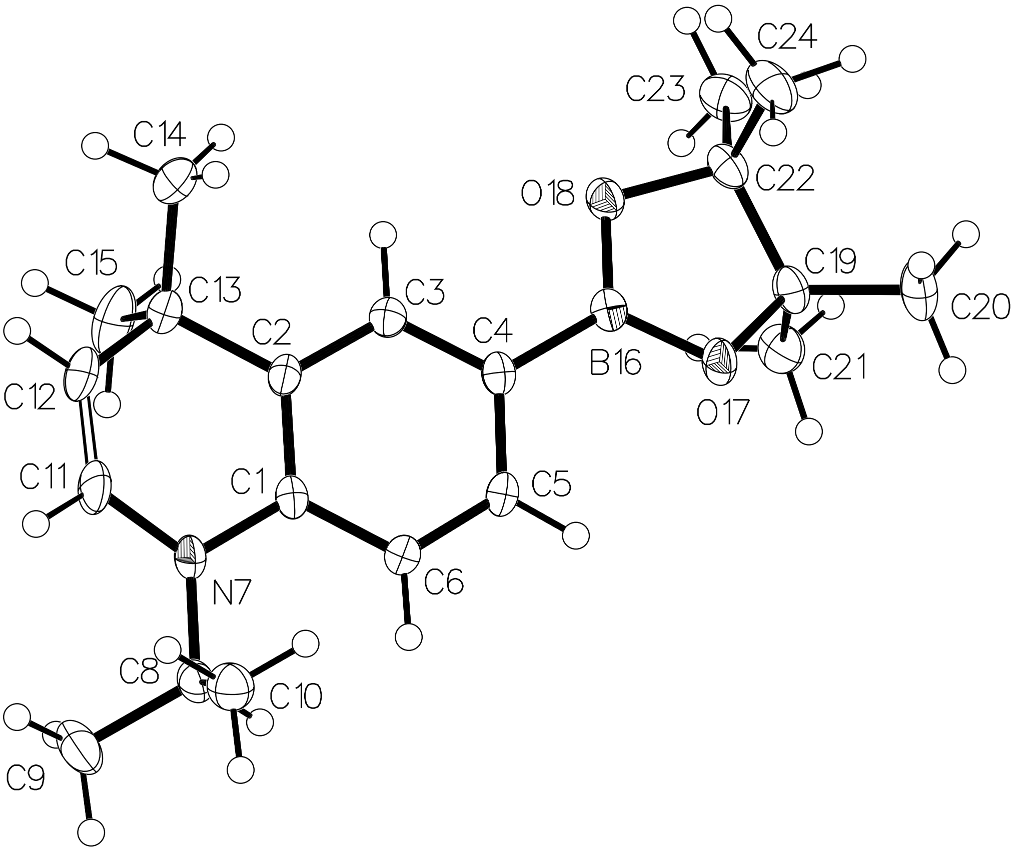

Figure S14: X-ray molecular structure of compound 30 (one of two molecules in the asymmetric unit). Thermal ellipsoids are drawn at the 50% probability level. Selected bond distances (Å): C(4)-B(16) 1.546, C(1)-N(7) 1.394, C(8)-N(7) 1.467, N(7)-C(11) 1.398. RMSD between the two molecules in the asymmetric unit (Å): 1.350 (without inversion), 0.252 (with inversion).

Product number	**13**	**14**	**15b**	**19**	**29**	**30**
Empirical formula	$C_{12}H_{14}INO$	$C_{12}H_{16}IN$	$C_{15}H_{20}INO$	$C_{11}H_{12}INO$	$C_{20}H_{32}BNO_2$	$C_{20}H_{30}BNO_2$
Formula weight	315.14	301.16	357.22	301.12	329.27	327.26
Temperature/K	120	120	120	120	120	120
Crystal system	monoclinic	monoclinic	monoclinic	monoclinic	monoclinic	monoclinic
Space group	$P2_1/c$	$P2_1/c$	$P2_1/n$	C2/c	$P2_1/c$	$P2_1/c$
a/Å	10.5231(9)	18.0060(8)	8.3580(8)	18.7230(4)	12.0435(6)	12.2051(5)
b/Å	12.4625(11)	9.8997(4)	15.7426(15)	8.16224(10)	11.5154(6)	11.3054(5)
c/Å	9.6181(8)	13.7872(6)	11.7029(11)	17.8772(4)	28.6939(14)	28.6657(12)
α/°	90	90	90	90	90	90
β/°	107.523(2)	107.361(2)	93.446(2)	126.024(3)	96.039(2)	95.011(2)
γ/°	90	90	90	90	90	90
Volume/$Å^3$	1202.83(18)	2345.66(18)	1537.0(3)	2209.57(11)	3957.4(3)	3940.3(3)
Z	4	8	4	8	8	8
$\rho_{calc}g/cm^3$	1.74	1.706	1.544	1.810	1.105	1.103
μ/mm^{-1}	2.637	2.694	2.073	2.866	0.069	0.069
F(000)	616	1184	712	1168.0	1440	1424
Crystal size/mm^3	0.317 × 0.304 × 0.190	0.266 × 0.165 × 0.160	0.183 × 0.158 × 0.081	0.521 × 0.365 × 0.212	0.312 × 0.309 × 0.204	0.216 × 0.188 × 0.082
Radiation	MoKα (λ = 0.71073)	MoKα (λ = 0.71073)	MoKα (λ = 0.71073)	MoKα (λ = 0.71073)	MoKα (λ = 0.71073)	MoKα (λ = 0.71073)
2Θ range for data collection/°	4.058 to 65.308	4.74 to 63.142	5.176 to 65.28	5.004 to 75.642	3.814 to 54.998	4.586 to 58.996
Index ranges	-15 ≤ h ≤ 15, -18 ≤ k ≤ 18, -14 ≤ l ≤ 14	-26 ≤ h ≤ 26, -14 ≤ k ≤ 14, -20 ≤ l ≤ 20	-12 ≤ h ≤ 12, -23 ≤ k ≤ 23, -17 ≤ l ≤ 17	-32 ≤ h ≤ 31, -13 ≤ k ≤ 13, -30 ≤ l ≤ 30	-15 ≤ h ≤ 15, -14 ≤ k ≤ 14, -37 ≤ l ≤ 37	-16 ≤ h ≤ 16, -15 ≤ k ≤ 15, -39 ≤ l ≤ 39
Reflections collected	20137	54918	24942	90313	70443	81825
Independent reflections	4392 [R_{int} = 0.0659, R_{sigma} = 0.0479]	7857 [R_{int} = 0.0311, R_{sigma} = 0.0196]	5530 [R_{int} = 0.0719, R_{sigma} = 0.0544]	5817 [R_{int} = 0.0350, R_{sigma} = 0.0129]	9094 [R_{int} = 0.0652, R_{sigma} = 0.0454]	10946 [R_{int} = 0.0548, R_{sigma} = 0.0422]
Data/restraints/parameters	4392/0/139	7857/0/259	5530/0/168	5817/0/129	9094/0/449	10946/0/449
Goodness-of-fit on F^2	1.04	1.081	1.05	1.088	1.039	1.034
Final R indexes [I>=2σ (I)]	R1 = 0.0532, wR2 = 0.1382	R_1 = 0.0279, wR_2 = 0.0590	R_1 = 0.0591, wR_2 = 0.1448	R_1 = 0.0190, wR_2 = 0.0470	R_1 = 0.0600, wR_2 = 0.1337	R_1 = 0.0547, wR_2 = 0.1250
Final R indexes [all data]	R1 = 0.0570, wR2 = 0.1426	R_1 = 0.0374, wR_2 = 0.0632	R_1 = 0.0664, wR_2 = 0.1508	R_1 = 0.0233, wR_2 = 0.0486	R_1 = 0.0919, wR_2 = 0.1484	R_1 = 0.0890, wR_2 = 0.1412
Largest diff. peak/hole / e $Å^{-3}$	2.72/-0.91	1.72/-2.80	2.73/-0.85	0.57/-0.46	0.51/-0.26	0.39/-0.19

Table S1: Crystallographic data for compounds 13, 14, 15b, 19, 29 and 30. CCDC (1433617-1433622) contains the full supplementary crystallographic data for this paper. The data can be obtained free of charge from The Cambridge Crystallographic Data Centre via www.ccdc.cam.ac.uk/getstructures [11–15].

6. References

(1) Rueping, M.; Theissmann, T.; Stoeckel, M.; Antonchick, A. P. *Org. Biomol. Chem.* **2011**, *9*, 6844–6850.

(2) Katritzky, A. R.; Rachwal, B.; Rachwal, S. *J. Org. Chem.* **1996**, *60*, 2588–2596.

(3) Compain, G.; Bonneau, C.; Martin-Mingot, A.; Thibaudeau, S. *J. Org. Chem.* **2013**, *78*, 4463–4472.

(4) Yang, B. H.; Buchwald, S. L. *Org. Lett.* **1999**, *1*, 35–37.

(5) Phippen, C. B. W.; Beattie, J. K.; McErlean, C. S. P. *Chem. Commun.* **2010**, *46* (43), 8234–8236.

(6) Jean-Francois, P.; Stephenson, C. R. J.; Defieber, C.; Carreira, E. M. *Org. Lett.* **2005**, *7* (6), 3821–3824.

(7) Conley, R. T.; Knopka, W. N. *J. Org. Chem.* **1964**, *29*, 496–497.

(8) Zhang, L.; Qureshi, Z.; Sonaglia, L.; Lautens, M. *Angew. Chem. Int. Ed.* **2014**, *53*, 13850–13853.

(9) Liu, B.; Fan, Y.; Gao, Y.; Sun, C.; Xu, C.; Zhu, J. *J. Am. Chem. Soc.* **2013**, *135*, 468–473.

(10) Yamamoto, T.; Morita, T.; Takagi, J.; Yamakawa, T. *Org. Lett.* **2011**, *13* (21), 5766–5769.

(11) Dolomanov, O. V.; Bourhis, L. J.; Gildea, R. J.; Howard, J. A. K.; Puschmann, H. *J. Appl. Crystallogr.* **2009**, *42* (2), 339–341.

(12) Sheldrick, G. M. *Acta Cryst.* **2008**, *A64*, 112–122.

(13) Sheldrick, G. M. *Acta Cryst.* **2015**, *A71* (1), 3–8.

(14) Sheldrick, G. M. *Acta Cryst.* **2015**, *C71* (1), 3–8.

(15) APEX2; SAINT; SADABS. *Bruker AXS Inc., Madison, Wisconsin, USA* **2014**.
