# Supplementary material for: Practical synthetic strategies towards lipophilic 6-iodotetrahydroquinolines and -dihydroquinolines
Source: Beilstein J Org Chem. 2016 Aug 16;12:1851–62. doi: 10.3762/bjoc.12.174 (PMC5082455; doi:10.3762/bjoc.12.174)

checkCIF/PLATON report

Structure factors have been supplied for datablock(s) ho1

THIS REPORT IS FOR GUIDANCE ONLY. IF USED AS PART OF A REVIEW PROCEDURE FOR PUBLICATION, IT SHOULD NOT REPLACE THE EXPERTISE OF AN EXPERIENCED CRYSTALLOGRAPHIC REFEREE.

No syntax errors found. CIF dictionary Interpreting this report

Datablock: ho1

```
Bond precision:  C-C = 0.0030 A          Wavelength=0.71073

Cell:           a=18.0060(8)    b=9.8997(4)         c=13.7872(6)
                alpha=90        beta=107.361(2)     gamma=90
Temperature:    120 K

                Calculated                Reported
Volume          2345.66(18)               2345.66(18)
Space group     P 21/c                    P 1 21/c 1
Hall group      -P 2ybc                   -P 2ybc
Moiety formula  C12 H16 I N               C12 H16 I N
Sum formula     C12 H16 I N               C12 H16 I N
Mr              301.16                    301.16
Dx,g cm-3       1.706                     1.706
Z               8                         8
Mu (mm-1)       2.694                     2.694
F000            1184.0                    1184.0
F000'           1180.54
h,k,lmax        26,14,20                  26,14,20
Nref            7859                      7857
Tmin,Tmax       0.592,0.650               0.931,1.000
Tmin'           0.484

Correction method= # Reported T Limits: Tmin=0.931 Tmax=1.000
AbsCorr = MULTI-SCAN

Data completeness= 1.000          Theta(max)= 31.571

R(reflections)= 0.0279( 6697)     wR2(reflections)= 0.0632( 7857)

S = 1.082                    Npar= 259
```

The following ALERTS were generated. Each ALERT has the format
test-name_ALERT_alert-type_alert-level.
Click on the hyperlinks for more details of the test.

Alert level C

```
PLAT420_ALERT_2_C D-H Without Acceptor        N8     -   H8      ..     Please Check
PLAT420_ALERT_2_C D-H Without Acceptor        N22    -   H22     ..     Please Check
PLAT971_ALERT_2_C Check Calcd Residual Density  0.69A From     I28       1.65 eA-3
PLAT972_ALERT_2_C Check Calcd Residual Density  0.81A From     I28      -2.43 eA-3
PLAT972_ALERT_2_C Check Calcd Residual Density  0.66A From     I14      -1.66 eA-3
PLAT975_ALERT_2_C Check Calcd Residual Density  0.91A From      N8       0.50 eA-3
```

Alert level G

```
PLAT007_ALERT_5_G Number of Unrefined Donor-H Atoms .............          2 Report
PLAT380_ALERT_4_G Incorrectly? Oriented X(sp2)-Methyl Moiety .....        C7 Check
PLAT380_ALERT_4_G Incorrectly? Oriented X(sp2)-Methyl Moiety .....       C21 Check
PLAT910_ALERT_3_G Missing # of FCF Reflection(s) Below Th(Min) ...         1 Report
```

0 **ALERT level A** = Most likely a serious problem - resolve or explain
0 **ALERT level B** = A potentially serious problem, consider carefully
6 **ALERT level C** = Check. Ensure it is not caused by an omission or oversight
4 **ALERT level G** = General information/check it is not something unexpected

0 ALERT type 1 CIF construction/syntax error, inconsistent or missing data
6 ALERT type 2 Indicator that the structure model may be wrong or deficient
1 ALERT type 3 Indicator that the structure quality may be low
2 ALERT type 4 Improvement, methodology, query or suggestion
1 ALERT type 5 Informative message, check

It is advisable to attempt to resolve as many as possible of the alerts in all categories. Often the minor alerts point to easily fixed oversights, errors and omissions in your CIF or refinement strategy, so attention to these fine details can be worthwhile. In order to resolve some of the more serious problems it may be necessary to carry out additional measurements or structure refinements. However, the purpose of your study may justify the reported deviations and the more serious of these should normally be commented upon in the discussion or experimental section of a paper or in the "special_details" fields of the CIF. checkCIF was carefully designed to identify outliers and unusual parameters, but every test has its limitations and alerts that are not important in a particular case may appear. Conversely, the absence of alerts does not guarantee there are no aspects of the results needing attention. It is up to the individual to critically assess their own results and, if necessary, seek expert advice.

Publication of your CIF in IUCr journals

A basic structural check has been run on your CIF. These basic checks will be run on all CIFs submitted for publication in IUCr journals (*Acta Crystallographica*, *Journal of Applied Crystallography*, *Journal of Synchrotron Radiation*); however, if you intend to submit to *Acta Crystallographica Section C* or *E*, you should make sure that full publication checks are run on the final version of your CIF prior to submission.

Publication of your CIF in other journals

Please refer to the *Notes for Authors* of the relevant journal for any special instructions relating to CIF submission.

PLATON version of 21/06/2015; check.def file version of 21/06/2015

Datablock ho1 - ellipsoid plot

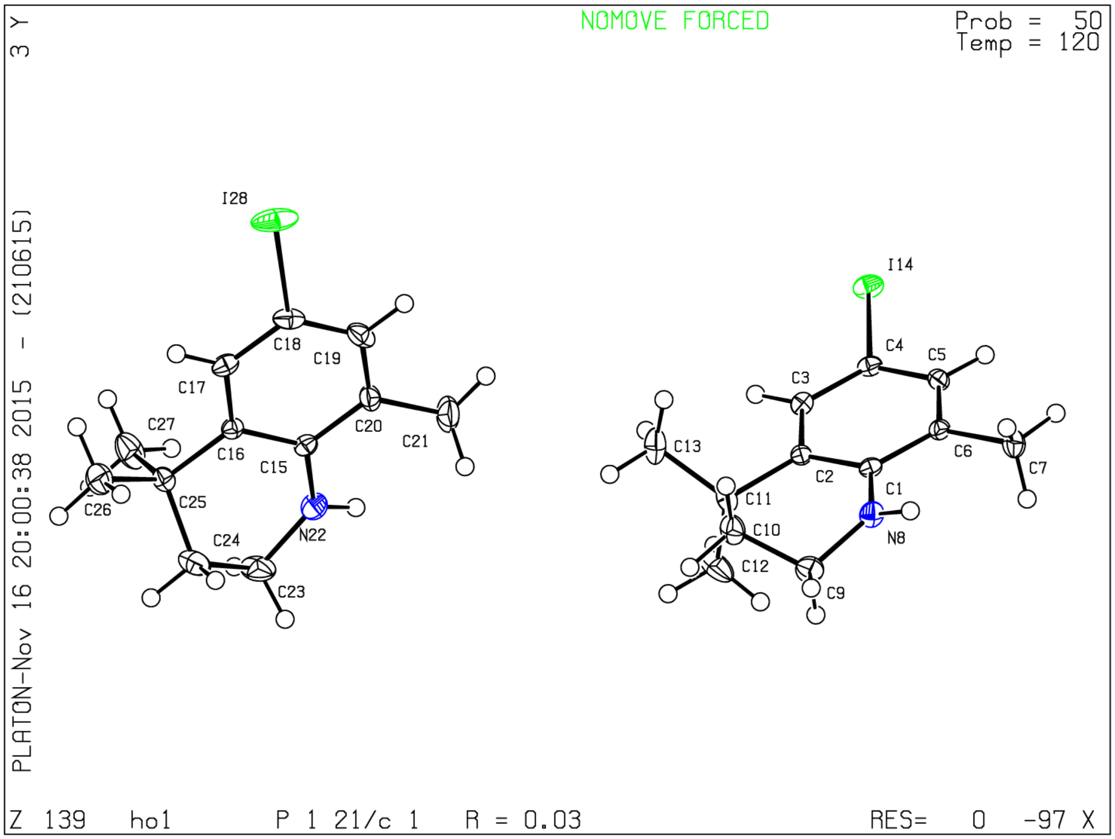

Supplement: File 2 — Crystallographic Information Files of compounds 13, 14, 15b, 19, 29, and 30. [file Beilstein_J_Org_Chem-12-1851-s002.zip › 14_1433619_cifreport.pdf]
