# Supplementary material for: Practical synthetic strategies towards lipophilic 6-iodotetrahydroquinolines and -dihydroquinolines
Source: Beilstein J Org Chem. 2016 Aug 16;12:1851–62. doi: 10.3762/bjoc.12.174 (PMC5082455; doi:10.3762/bjoc.12.174)

checkCIF/PLATON report

Structure factors have been supplied for datablock(s) mo_dcoisopropylface

THIS REPORT IS FOR GUIDANCE ONLY. IF USED AS PART OF A REVIEW PROCEDURE FOR PUBLICATION, IT SHOULD NOT REPLACE THE EXPERTISE OF AN EXPERIENCED CRYSTALLOGRAPHIC REFEREE.

No syntax errors found. CIF dictionary Interpreting this report

Datablock: mo_dcoisopropylface

Bond precision: C-C = 0.0031 A Wavelength=0.71073

Cell:	a=8.3580(8)	b=15.7426(15)	c=11.7029(11)
	alpha=90	beta=93.446(2)	gamma=90
Temperature:	120 K		

	Calculated	Reported
Volume	1537.0(3)	1537.0(3)
Space group	P 21/n	P 1 21/n 1
Hall group	-P 2yn	-P 2yn
Moiety formula	C15 H20 I N O	C15 H20 I N O
Sum formula	C15 H20 I N O	C15 H20 I N O
Mr	357.22	357.22
Dx,g cm-3	1.544	1.544
Z	4	4
Mu (mm-1)	2.073	2.073
F000	712.0	712.0
F000’	710.33	
h,k,lmax	12,23,17	12,23,17
Nref	5623	5530
Tmin,Tmax	0.691,0.845	0.630,0.896
Tmin’	0.678	

Correction method= # Reported T Limits: Tmin=0.630 Tmax=0.896
AbsCorr = NUMERICAL

Data completeness= 0.983 Theta(max)= 32.640

R(reflections)= 0.0591(4789) wR2(reflections)= 0.1508(5530)

S = 1.050 Npar= 168

The following ALERTS were generated. Each ALERT has the format
test-name_ALERT_alert-type_alert-level.
Click on the hyperlinks for more details of the test.

Alert level B

```
PLAT971_ALERT_2_B Check Calcd Residual Density  0.05A From     I18        2.90 eA-3
PLAT971_ALERT_2_B Check Calcd Residual Density  0.53A From     I18        2.65 eA-3
PLAT971_ALERT_2_B Check Calcd Residual Density  0.61A From     I18        2.63 eA-3
```

Alert level C

```
PLAT094_ALERT_2_C Ratio of Maximum / Minimum Residual Density ....        3.23 Report
PLAT911_ALERT_3_C Missing # FCF Refl Between THmin & STh/L=  0.600           3 Report
PLAT971_ALERT_2_C Check Calcd Residual Density  0.63A From     I18        2.48 eA-3
PLAT971_ALERT_2_C Check Calcd Residual Density  0.57A From     I18        2.48 eA-3
PLAT971_ALERT_2_C Check Calcd Residual Density  0.65A From     I18        2.33 eA-3
```

Alert level G

```
PLAT072_ALERT_2_G SHELXL First  Parameter in WGHT Unusually Large.        0.11 Report
PLAT431_ALERT_2_G Short Inter HL..A  Contact I18    ..  N8       ..       3.22 Ang.
PLAT910_ALERT_3_G Missing # of FCF Reflection(s) Below Th(Min) ...           1 Report
PLAT912_ALERT_4_G Missing # of FCF Reflections Above STh/L=  0.600          90 Note
```

0 **ALERT level A** = Most likely a serious problem - resolve or explain
3 **ALERT level B** = A potentially serious problem, consider carefully
5 **ALERT level C** = Check. Ensure it is not caused by an omission or oversight
4 **ALERT level G** = General information/check it is not something unexpected

0 ALERT type 1 CIF construction/syntax error, inconsistent or missing data
9 ALERT type 2 Indicator that the structure model may be wrong or deficient
2 ALERT type 3 Indicator that the structure quality may be low
1 ALERT type 4 Improvement, methodology, query or suggestion
0 ALERT type 5 Informative message, check

It is advisable to attempt to resolve as many as possible of the alerts in all categories. Often the minor alerts point to easily fixed oversights, errors and omissions in your CIF or refinement strategy, so attention to these fine details can be worthwhile. In order to resolve some of the more serious problems it may be necessary to carry out additional measurements or structure refinements. However, the purpose of your study may justify the reported deviations and the more serious of these should normally be commented upon in the discussion or experimental section of a paper or in the "special_details" fields of the CIF. checkCIF was carefully designed to identify outliers and unusual parameters, but every test has its limitations and alerts that are not important in a particular case may appear. Conversely, the absence of alerts does not guarantee there are no aspects of the results needing attention. It is up to the individual to critically assess their own results and, if necessary, seek expert advice.

Publication of your CIF in IUCr journals

A basic structural check has been run on your CIF. These basic checks will be run on all CIFs submitted for publication in IUCr journals (*Acta Crystallographica*, *Journal of Applied Crystallography*, *Journal of Synchrotron Radiation*); however, if you intend to submit to *Acta Crystallographica Section C* or *E*, you should make sure that full publication checks are run on the final version of your CIF prior to submission.

Publication of your CIF in other journals

Please refer to the *Notes for Authors* of the relevant journal for any special instructions relating to CIF submission.

PLATON version of 21/06/2015; check.def file version of 21/06/2015

Datablock mo_dcoisopropylface - ellipsoid plot

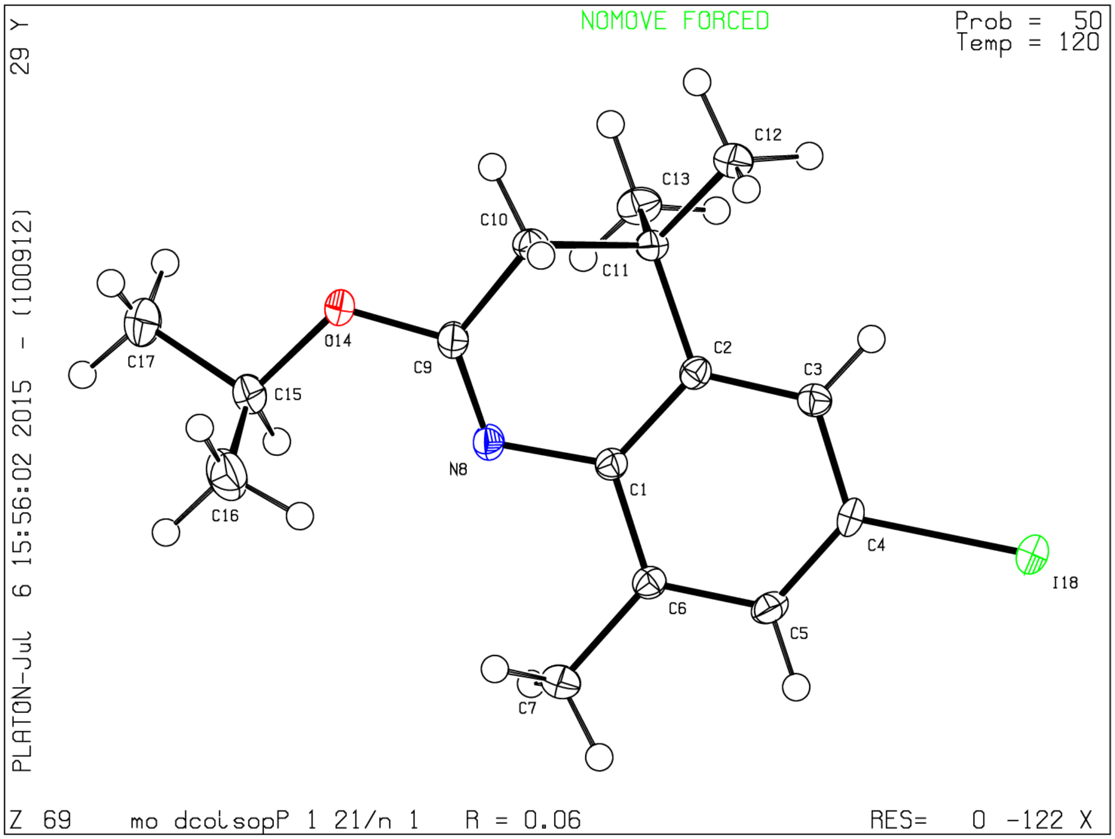

Supplement: File 2 — Crystallographic Information Files of compounds 13, 14, 15b, 19, 29, and 30. [file Beilstein_J_Org_Chem-12-1851-s002.zip › 15b_1433618_cifreport.pdf]
