# Supplementary material for: Practical synthetic strategies towards lipophilic 6-iodotetrahydroquinolines and -dihydroquinolines
Source: Beilstein J Org Chem. 2016 Aug 16;12:1851–62. doi: 10.3762/bjoc.12.174 (PMC5082455; doi:10.3762/bjoc.12.174)

checkCIF/PLATON report

Structure factors have been supplied for datablock(s) dc248

THIS REPORT IS FOR GUIDANCE ONLY. IF USED AS PART OF A REVIEW PROCEDURE FOR PUBLICATION, IT SHOULD NOT REPLACE THE EXPERTISE OF AN EXPERIENCED CRYSTALLOGRAPHIC REFEREE.

No syntax errors found.   CIF dictionary   Interpreting this report

Datablock: dc248

```
Bond precision:  C-C = 0.0016 A          Wavelength=0.71073

Cell:           a=18.7230(4)    b=8.16224(10)      c=17.8772(4)
                alpha=90        beta=126.024(3)    gamma=90
Temperature:    120 K

                Calculated                Reported
Volume          2209.58(11)               2209.57(11)
Space group     C 2/c                     C 1 2/c 1
Hall group      -C 2yc                    -C 2yc
Moiety formula  C11 H12 I N O             C11 H12 I N O
Sum formula     C11 H12 I N O             C11 H12 I N O
Mr              301.12                    301.12
Dx,g cm-3       1.810                     1.810
Z               8                         8
Mu (mm-1)       2.866                     2.866
F000            1168.0                    1168.0
F000'           1164.61
h,k,lmax        32,14,30                  32,13,30
Nref            5942                      5817
Tmin,Tmax       0.296,0.545               0.375,0.609
Tmin'           0.216

Correction method= # Reported T Limits: Tmin=0.375 Tmax=0.609
AbsCorr = ANALYTICAL

Data completeness= 0.979          Theta(max)= 37.821

R(reflections)= 0.0190( 5216)     wR2(reflections)= 0.0489( 5817)

S = 1.093                   Npar= 163
```

The following ALERTS were generated. Each ALERT has the format
test-name_ALERT_alert-type_alert-level.
Click on the hyperlinks for more details of the test.

Alert level G

```
PLAT128_ALERT_4_G Alternate Setting for Input Space Group     C2/c    I2/c    Note
PLAT912_ALERT_4_G Missing # of FCF Reflections Above STh/L=  0.600        114 Note
PLAT953_ALERT_1_G Reported (CIF) and Actual (FCF) Hmax Differ by .          1 Units
```

```
   0 ALERT level A = Most likely a serious problem - resolve or explain
   0 ALERT level B = A potentially serious problem, consider carefully
   0 ALERT level C = Check. Ensure it is not caused by an omission or oversight
   3 ALERT level G = General information/check it is not something unexpected

   1 ALERT type 1 CIF construction/syntax error, inconsistent or missing data
   0 ALERT type 2 Indicator that the structure model may be wrong or deficient
   0 ALERT type 3 Indicator that the structure quality may be low
   2 ALERT type 4 Improvement, methodology, query or suggestion
   0 ALERT type 5 Informative message, check
```

It is advisable to attempt to resolve as many as possible of the alerts in all categories. Often the minor alerts point to easily fixed oversights, errors and omissions in your CIF or refinement strategy, so attention to these fine details can be worthwhile. In order to resolve some of the more serious problems it may be necessary to carry out additional measurements or structure refinements. However, the purpose of your study may justify the reported deviations and the more serious of these should normally be commented upon in the discussion or experimental section of a paper or in the "special_details" fields of the CIF. checkCIF was carefully designed to identify outliers and unusual parameters, but every test has its limitations and alerts that are not important in a particular case may appear. Conversely, the absence of alerts does not guarantee there are no aspects of the results needing attention. It is up to the individual to critically assess their own results and, if necessary, seek expert advice.

Publication of your CIF in IUCr journals

A basic structural check has been run on your CIF. These basic checks will be run on all CIFs submitted for publication in IUCr journals (*Acta Crystallographica*, *Journal of Applied Crystallography*, *Journal of Synchrotron Radiation*); however, if you intend to submit to *Acta Crystallographica Section C* or *E*, you should make sure that full publication checks are run on the final version of your CIF prior to submission.

Publication of your CIF in other journals

Please refer to the *Notes for Authors* of the relevant journal for any special instructions relating to CIF submission.

PLATON version of 21/04/2015; check.def file version of 09/03/2015

Datablock dc248 - ellipsoid plot

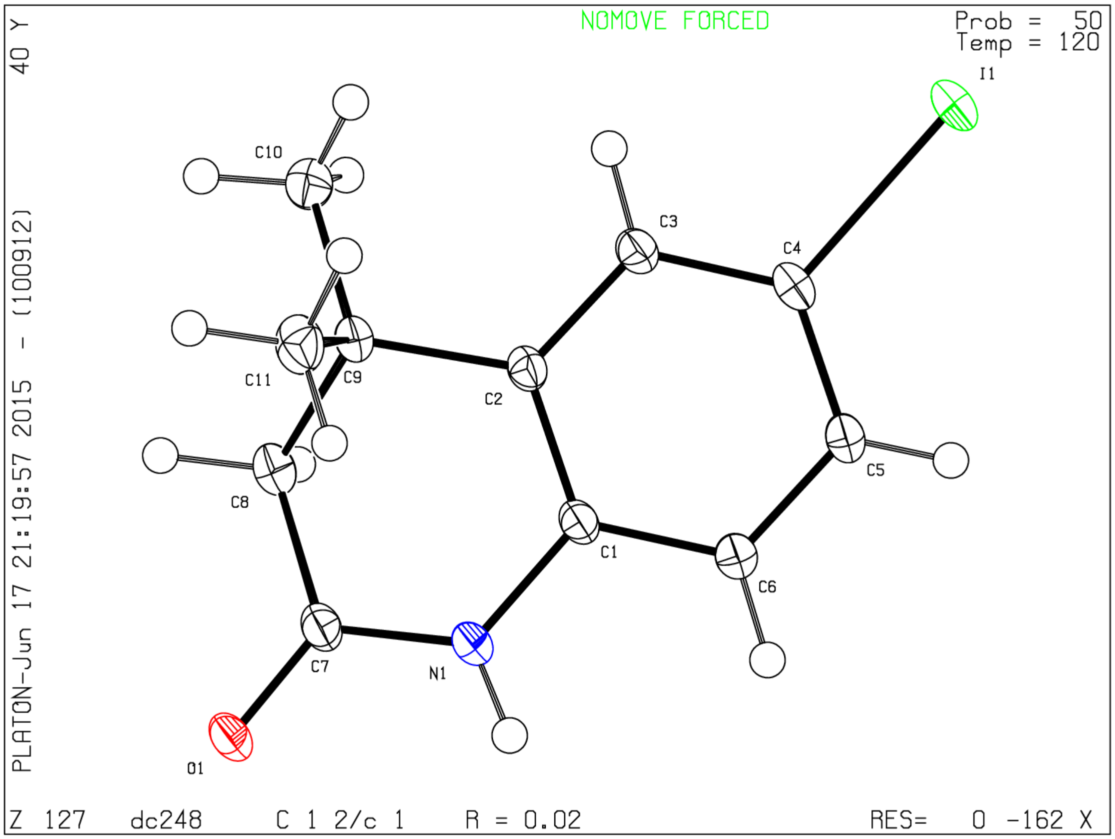

Supplement: File 2 — Crystallographic Information Files of compounds 13, 14, 15b, 19, 29, and 30. [file Beilstein_J_Org_Chem-12-1851-s002.zip › 19_1433618_cifreport.pdf]
